# Non-communicable disease burden through adhering to Dutch dietary guidelines: a modeling study to estimate future reductions

**DOI:** 10.1016/j.eclinm.2025.103170

**Published:** 2025-03-27

**Authors:** Ming-Jie Duan, Maartje P. Poelman, Sander Biesbroek

**Affiliations:** a Division of Human Nutrition and Health, Wageningen University & Research, Wageningen, the Netherlands; b Chair Group Consumption and Healthy Lifestyles, Wageningen University & Research, Wageningen, the Netherlands

**Keywords:** Non-communicable diseases, Disease burden, Health projection, Food consumption, Dietary guidelines, Modeling study

## Abstract

**Background:**

Non-optimal food consumption is a major modifiable risk factor for non-communicable diseases (NCDs). We aimed to estimate the potential reductions in NCD burden that could be achieved in 2050 by adhering to eleven components of the Dutch dietary guidelines in the Netherlands.

**Methods:**

Counterfactual scenarios, in which the entire Dutch population adhered to components of the dietary guidelines, were implemented to estimate future reductions in NCD burden using the DYNAMO-HIA software. Input data included nationally-representative food consumption data (Dutch National Food Consumption Survey 2012–2016), relative risks of food consumption on NCDs, Dutch NCD epidemiology, and the Dutch population structure.

**Findings:**

Adhering to processed meat (0 g/d) and fruit (≥200 g/d) recommendations showed the largest reductions. By 2050, eliminating processed meat consumption could reduce per 100,000 population cases of coronary heart disease by 18.5% (95% Uncertainty Interval [UI]: 3.9%, 36.2%) in women and 23.8% (95% UI: 5.7%, 44.1%) in men, type 2 diabetes by 18.6% (95% UI: 11.6%, 27.4%) in women and 24.5% (95% UI: 15.3%, 35.4%) in men, and colorectal cancer by 8.7% (95% UI: 5.1%, 13.0%) in women and 10.4% (95% UI: 5.0%, 16.4%) in men. Adhering to fruit recommendation could reduce stroke cases by 17.7% (95% UI: 11.1%, 21.9%) in women and 19.1% (95% UI: 12.2%, 23.4%) in men, and lung cancer cases by 8.2% (95% UI: 4.4%, 12.8%) in women and 7.2% (95% UI: 3.4%, 12.0%) in men. Eliminating processed meat could increase life expectancy at age 50 by 0.310 (95% UI: 0.181, 0.453) years for women and 0.724 (95% UI: 0.398, 1.092) years for men, and disease-free life expectancy by 1.554 (95% UI: 1.032, 2.189) years for women and 2.494 (95% UI: 1.627, 3.504) years for men.

**Interpretation:**

Our model suggests that adhering to Dutch dietary guidelines could reduce future NCD burden in the Netherlands. Reducing processed meat and increasing fruit consumption should be priority prevention targets.

**Funding:**

This project has received funding from the Dutch Science Agenda (NWA) program ‘Transition Towards a Sustainable Food System’ funded by the 10.13039/501100003246Dutch Research Council (NWO): NWA.1235.18.201.

**Research in context**

**Evidence before this study**

We searched PubMed (until December 1, 2024) for studies on estimating non-communicable disease (NCD) burden related to food consumption. We used the search terms: ((Population health [MeSH Terms]) OR (life expectancy [MeSH Terms]) OR (disease burden∗[Title/Abstract])) AND ((diet∗[Title/Abstract]) OR (diet [MeSH Terms]) OR (Diet, Food, and Nutrition [MeSH Terms]) OR (Diet, Healthy [MeSH Terms]) OR (dietary patterns [MeSH Terms]) OR (Food [MeSH Terms]) OR (Nutrition Policy [MeSH Terms]) OR (dietary guidelines)) AND ((coronary heart disease [MeSH Terms]) OR (colorectal cancer [MeSH Terms]) OR (type 2 diabetes mellitus [MeSH Terms]) OR (stroke [MeSH Terms]) OR (cardiovascular disease [MeSH Terms]) OR (lung cancer [MeSH Terms])) with no language or time restrictions.

Previous studies have shown that non-optimal food consumption—both *past* and *current*—is a major contributor to the burden of NCDs. However, less is known about the extent to which adherence to healthy dietary guidelines may reduce the *future* burden of NCDs. While many studies have focused on individual NCDs and mortality, a comprehensive assessment of the overall disease burden and life expectancy remains limited.

**Added value of this study**

This study is the first to assess the potential future reductions in NCD burden and gains in life expectancy in the Netherlands achievable through adherence to the Dutch dietary guidelines. Our analysis provides projections for future population health, suggesting that adhering to these guidelines could reduce cases of coronary heart disease, stroke, type 2 diabetes, colorectal cancer, and lung cancer by 2050. Adherence to these guidelines could also increase both total and disease-free life expectancy. Reducing processed meat and increasing fruit consumption could yield the largest future population health gains.

**Implications of all the available evidence**

Adhering to dietary guidelines, particularly processed meat and fruit, could reduce the future burden of NCDs and improve population health. Taken together, all evidence supports the development of forward-thinking, evidence-based health policies to effectively address non-optimal diets by adhering to dietary guidelines as a fundamental strategy for NCD prevention at the population level.

## Introduction

Non-optimal food consumption is a major modifiable risk factor for the development of non-communicable diseases (NCDs). Insufficient consumption of fruit, vegetables, whole grains, legumes, nuts/seeds, fish, and tea, as well as excessive consumption of red meat, processed meat, and sugary beverages, have been associated with increased risks of coronary heart disease, stroke, type 2 diabetes, lung cancer, and colorectal cancer.^1^, ^2^, ^3^ The 2015 Dutch food-based dietary guidelines provide recommendations on the optimal consumption levels for these food groups.^1^ These recommendations are largely based on evidence from prospective observational studies. However, it remains unclear how the relative risks (RRs) derived from observational studies translate to the potential reductions in disease burden that could be achieved by adhering to these dietary guidelines.

In the Netherlands, the persistently high NCD burden has been clearly outlined as one of the major future public health challenges.^4^ It is estimated that in 2040, approximately 1.5 million people in the Netherlands will have diabetes and 1.1 million will have coronary heart disease, with cancer and cardiovascular diseases projected to be the leading causes of death.^5^ To tackle these challenges, it is essential to have a thorough understanding of how optimizing food consumption can help reduce this NCD burden and its future impact. This knowledge will support the identification of critical dietary targets, enabling evidence-based prioritization for prevention resource allocation and policy development. The Global Burden of Disease study (GBD), along with several other studies, has quantified the past and current NCD burden attributed to suboptimal diets.^2^^,^^6^, ^7^, ^8^ However, a comprehensive assessment of the potential future long-term reductions in NCD burden and gains in population health that could be achieved through dietary improvements is still lacking.

Therefore, in this study, we aimed to quantify the potential reductions in NCD burden and gains in population health that could be achieved by adhering to components of the Dutch dietary guidelines in the Netherlands over a 30-year period by 2050. We assumed that changes in food consumption levels to optimal levels defined by dietary guidelines would lower population NCD risk, thus reducing NCD incidence, prevalence, and mortality, which would ultimately decrease the overall NCD burden. We estimated the effects of each food group separately, aiming to identify priority policy targets for population dietary changes. The outcome measures include preventable NCD cases, as well as gains in life expectancy and disease-free life expectancy.

## Methods

### Food groups, optimal consumption level, and food consumption data

The health effects of a total of eleven food groups were separately assessed, including fruit, vegetables, legumes, whole grains, nuts/seeds, red meat, processed meat, fish, dairy products, sugary beverages, and tea. The 2015 Dutch food-based dietary guidelines and the Wheel of Five (the visualized version of Dutch dietary guidelines) provided the recommended optimal consumption levels for each food group,^1^^,^^9^ which are summarized in Table 1 and Supplementary Text S1 in more detail. The 2015 Dutch dietary guidelines also provided recommendations for coffee, fats/oils, alcoholic beverages, and salt. However, their health effects were not assessed; detailed reasons for this can be found in Supplementary Text S2.

**Table 1:** Consumption levels of food groups and adherence to the Dutch dietary guidelines.^a^

	Recommended consumption, g/d^b^	Women		Men	
		Consumption, g/d	Met recommendations, %	Consumption, g/d	Met recommendations, %
Fruit	≥200	103.3 (34.5, 202.0)	25.3	82.5 (0, 180.9)	21.3
Vegetables	≥200	131.7 (75.3, 201.5)	25.5	131.7 (81.4, 204.0)	25.8
Whole grains	≥90	84.5 (46.5, 125.0)	45.4	105 (52.5, 175.0)	59.6
Nuts/seeds	≥15	0 (0, 4.9)	15.5	0 (0, 7.7)	19.6
Legumes^c^	≥10	0 (0, 0)	12.0	0 (0, 0)	10.5
Fish	≥15	0 (0, 15.0)	25.0	0 (0, 15.2)	25.2
Red meat	≤45	14.5 (0, 44.2)	75.7	25 (0, 63.7)	65.7
Processed meat	0	28.9 (7.7, 59.0)	18.6	44.3 (17.0, 86.5)	13.0
Dairy products	300–450	276.5 (146.7, 429.2)	23.3	314.8 (163.5, 504.3)	21.4
Tea	≥600 (≥3 cups)	206.3 (0, 516.7)	20.3	0 (0, 281.3)	10.6
Sugary beverages	0	103 (0, 249.7)	35.4	154.5 (0, 375.0)	29.8

a Data were derived from the Dutch National Food Consumption Survey 2012–2016. All adult participants (19–79 years) were included: men *n* = 1043, women *n* = 1035. Consumption levels were presented as median (interquartile).

b Recommended consumption levels for food groups were based on the 2015 Dutch food-based dietary guidelines and the Wheel of Five (visual representation of the Dutch dietary guidelines).

c Range of consumption for legumes: men 0–198.3 g/d, women 0–409.4 g/d.

The food consumption data was obtained from the Dutch National Food Consumption Survey (DNFCS) 2012–2016.^10^ The survey sample was representative for the population in the Netherlands concerning age, education level, region of residence, and diet across a calendar year (season and days of the week). Participants were invited via an invitation letter, an information leaflet, and a reply card. Questionnaires were subsequently sent to those who agreed to participate. Dietary consumption was assessed with two 24 h dietary recalls on two non-consecutive days. Standardized interviews were conducted by trained dieticians using the GloboDiet software (IARC, Lyon, France). In this study, consumption data of all adult participants were included in the analysis (*n* = 2078, 19–79 years). The categorization of food products is shown in Supplementary Table S1. The overall design and rationale of the DNFCS have been described in detail elsewhere.^10^

### Effect size of food consumption on NCD endpoints

Effects of food consumption on five NCDs were assessed, including coronary heart disease, stroke, type 2 diabetes, colorectal cancer, and lung cancer. There is abundant evidence supporting the potential causal associations between specific food groups and certain NCD endpoints (Supplementary Table S2).^1^, ^2^, ^3^ The quality and strength of the epidemiological evidence for each food-NCD pair have been systematically evaluated by the GBD study and the 2015 Dutch food-based dietary guidelines.^1^^,^^2^ While food groups assessed are not always associated with all five NCDs, those NCDs not associated with the food group under assessment were also considered in the analysis, since changes in the burden of one disease may influence the burden of the others. To ensure the analysis was based on the best available evidence, RR estimates for each food-NCD pair were obtained from the GBD study.^2^ In cases where RR estimates were not available from the GBD study, a literature search was conducted on PubMed (dated July 1, 2024) to identify the most up-to-date meta-analyses. Dose–response RR estimates obtained from these meta-analyses were subsequently used in the analysis. The detailed literature search terms and identified meta-analyses are provided in Supplementary Text S3. For type 2 diabetes, coronary heart disease, and stroke, age-specific RRs of food on these three NCDs were used for the analysis, taking into account the associations between diet and metabolic risk factors, as well as the age-dependent associations between metabolic risk factors and these three NCDs.^11^ The GBD method for calculating the age-specific RRs was followed (see Supplementary Text S4 for a detailed description of this method).^2^^,^^11^ The RRs for food-NCD pairs are summarized in Supplementary Table S2.

### Estimating reductions in NCD burden: a counterfactual scenario approach

**Fig. 1:**
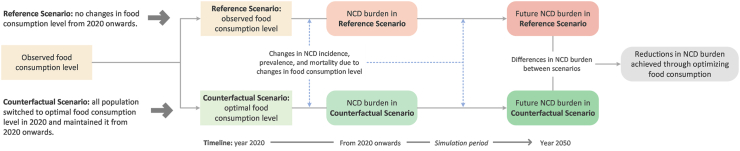
Graphical illustration of the counterfactual scenario approach for assessing the effects of optimizing food consumption on reducing non-communicable disease (NCD) burden.

A counterfactual scenario approach was applied using the DYNAMO-HIA (DYNAmic MOdeling for Health Impact Assessment) software (version 2.0.8; RIVM, Bilthoven, The Netherlands; www.dynamo-hia.eu/)^12^, ^13^, ^14^, ^15^ to assess the future reductions in NCD burden if the entire population in the Netherlands adhered to dietary guidelines over a 30-year period, starting from 2020. The health effects of each food group were assessed individually. In a reference “business-as-usual” scenario, the consumption level for the food group assessed was the observed consumption level from the population. In a counterfactual alternative scenario, the entire population were assumed to shift to the optimal consumption level for that food group according to dietary guidelines at the start of the simulation year 2020, therefore eliminating any health burden attributed to this particular food group. For other food groups, consumption levels were not specified and assumed to remain unchanged throughout the simulation period. Assuming that there were no further changes in the consumption level of the food group under assessment in both scenarios over time, the differences in NCD burden between the two scenarios throughout the simulation period thus represent the projected reductions in NCD burden that could be achieved by adhering to dietary guidelines.^12^, ^13^, ^14^, ^15^ Fig. 1 presents a graphical illustration of the counterfactual scenario approach applied in the current analysis.

DYNAMO-HIA is generic software used to quantify the future population health of intended health programs, policies, or interventions. It can be applied to various risk factors, diseases, and population settings.^12^^,^^16^^,^^17^ The technical core of DYNAMO-HIA is a Markov-type model that applies a multi-state partial-simulation model to simulate a real-life population. A more detailed description of the technical core of DYNAMO-HIA is available in Supplementary Text S5 and elsewhere.^12^, ^13^, ^14^, ^15^ DYNAMO-HIA is freely available online (www.dynamo-hia.eu/).

### Statistics

In this study, DYNAMO-HIA calculated the effects of presumed changes in the consumption level of a food group (exposure) on disease incidence, prevalence, and disease-related mortality, using the known RR estimates of the food group on disease incidence, along with the data on disease epidemiology and population structure. The entire population in the Netherlands was modeled (up to 95 years), considering changes in the population structure (including newborns and deaths but without migration). Each individual in the population was assigned to a food consumption level category (risk-factor state) specific to their age and sex group, based on the observed food consumption level for the reference scenario and the optimal food consumption level for the counterfactual scenario. Throughout the simulation period, the food consumption level category of each individual was updated yearly, based on the age- and sex-specific transition probabilities between food consumption level categories. In both the reference and counterfactual scenarios, transition probabilities were assigned to keep the age- and sex-specific food consumption level of the population (risk factor prevalence) constant. The trajectory of the risk-factor states throughout the simulation period of each individual forms a unique risk-factor biography.^12^^,^^13^ The simulation of the large number of risk-factor biographies of the entire population was enabled by the micro-simulation module of DYNAMO-HIA.

In the macro-simulation module, separate life tables were constructed for each risk-factor biography, accounting for competing risks and multi-morbidity. The relative risks of an individual to develop a NCD were determined by the current age- and sex-specific food consumption level (risk-factor state). Three health states were specified: NCD-free, having NCDs, and death. For each NCD, the incidence—transitioning from NCD-free to having a NCD—was calculated by multiplying the incidence at baseline by the age-specific RRs of the food consumption level and disease status. The transition from NCD-free to death was equivalent to the other-cause mortality at baseline of the NCD-free population, calculated as the excess mortality rate of the NCD modeled subtract from the age- and sex-specific total mortality rate. The transition from having a NCD to death was the sum of the excess mortality of the NCD modeled and the other-cause mortality of the NCD-free population at baseline. Remission of NCDs was not modeled. For coronary heart disease and stroke, acutely fatal cases were specified as the fraction of incident cases resulting in immediate death. For each risk-factor biography, life tables were calculated separately for each birth cohort of all individuals born in the same calendar year. For those born before 2020 (the start of the simulation year), NCD prevalence was specified using existing NCD epidemiology data for the population in the Netherlands. For newborns, risk-factor biographies were simulated, and corresponding disease life tables were subsequently calculated. The NCD burden estimates of the whole population were obtained by aggregating the life tables for all risk-factor biographies.^12^^,^^13^

To quantify reductions in NCD burden, calculations were made for preventable NCD cases, as well as gains in total and disease-free life expectancy achieved by adhering to dietary guidelines from the aggregated life tables. Disease-free life expectancy is defined as life expectancy free of any of the modeled NCDs. Input of this analysis included the observed and optimal consumption level of food groups, RRs of food consumption level on NCD endpoints, NCD epidemiology, and data on population structure in the Netherlands. All data were stratified by age and sex. A summary of the input data used in this analysis is available in Supplementary Text S6.

### Data on NCD epidemiology and population structure in the Netherlands

Data on NCD epidemiology in the Netherlands, including incidence, prevalence, and disease-related mortality, were collected and processed by the DYNAMO-HIA consortium from various sources.^13^ This data is openly available from the DYNAMO-HIA website (www.dynamo-hia.eu/en/reference-data). Data for coronary heart disease, stroke, and type 2 diabetes from 2011 were obtained from the Netherlands Information Network of General Practice.^13^^,^^18^^,^^19^ These data were further linked to the national mortality registry from the Statistics Netherlands (CBS) to derive data on disease-related mortality (opendata.cbs.nl). For coronary heart disease and stroke, data from hospital records (1995–2010) were also used to identify acute incident cases. Mortality for coronary heart disease and stroke was split into short-term (less than 1 year) and long-term mortality, with acute mortality derived by subtracting long-term mortality from mortality over one year.^19^ Data on lung cancer and colorectal cancer (1989–2011) were obtained from the Netherlands Cancer Registry (www.ikcnet.nl). Poisson regression models (including time trends) were used to estimate age- and sex-specific incidence rates and mortality in 2011.^19^ Data on the 2020 population structure, including population size, newborns, and mortality for each age and sex, were obtained from CBS. The aforementioned NCD epidemiology data from 2011 were applied to the 2020 Dutch population in the analysis. We consider it unlikely that substantial changes in NCD epidemiology occurred due to alterations in risk factors, treatment, or disease diagnosis in the population during this time period between 2011 and 2020.

### Uncertainty analysis

To assess the uncertainties of the model estimates concerning the associations between food groups and NCD endpoints, Monte Carlo simulations were conducted. The calculations were repeated 100 times for each food group, using a single draw of the RR estimates from their respective 95% confidence intervals (Supplementary Table S2). Based on the results of these 100 calculations, the 95% uncertainty intervals (UIs) were calculated for all the estimated outcome measures.^12^

### Ethics

The Medical Ethical Committee of the University Medical Center Utrecht, the Netherlands, concluded that the Dutch National Food Consumption Survey 2012–2016 was exempt from ethics approval due to its non-invasive measurements, in accordance with the 'Medical Research Involving Human Subjects Act’ (WMO) (reference number 12–359/C). Informed consent from participants was waived.

### Role of funding source

The funder had no role in any part of this research.

## Results

Table 1 shows the food consumption levels and the prevalence of adhering to each component of the Dutch dietary guidelines in the Netherlands. For all food groups assessed, majority of the population did not meet the recommended optimal consumption levels from the dietary guidelines. For both women and men, the lowest adherence was observed for legumes (≥10 g/d: 12.0% women, 10.5% men), tea (≥600 g/d: 20.3% women, 10.6% men), processed meat (0 g/d: 18.6% women, 13.0% men), and nuts/seeds (≥15 g/d: 15.5% women, 19.6% men). The highest adherence was observed for red meat (≤45 g/d: 75.7% women, 65.7% men) and whole grains (≥90 g/d: 45.4% women, 59.6% men).

**Fig. 2:**
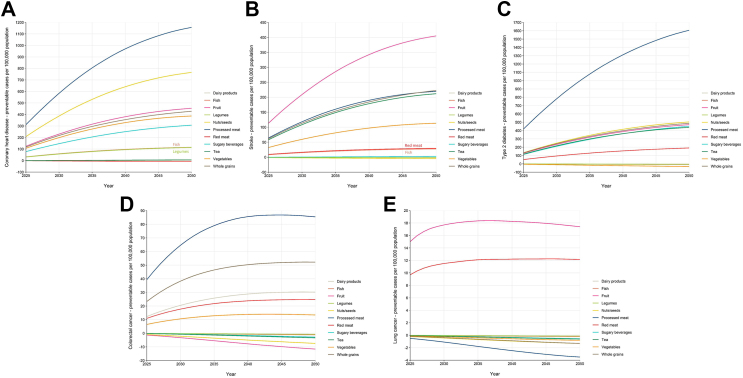
Projected number of preventable non-communicable disease cases per 100,000 population over a 30-year period (by 2050) if the entire population adhered to each component of the Dutch dietary guidelines **(a)** Coronary heart disease. **(b)** Stroke. **(c)** Type 2 diabetes. **(d)** Colorectal cancer. **(e)** Lung cancer.

Table 2 and Fig. 2 present the projected reductions in NCD cases in the Netherlands over a 30-year period (by 2050) that could be achieved if the entire population adhered to the components of dietary guidelines. Adhering to recommendations for processed meat and fruit consumption is projected to have the most significant impact on preventing NCDs. Specifically, limiting processed meat consumption to 0 g/d could result in the largest reduction in coronary heart disease cases, with estimated decreases of 859 cases (95% UI: 180, 1676), equivalent to 18.5% (95% UI: 3.9%, 36.2%), per 100,000 women, and 1457 cases (95% UI: 349, 2700) or 23.8% (95% UI: 5.7%, 44.1%) per 100,000 men. Limiting processed meat consumption could also lead to the largest reduction in type 2 diabetes cases, with potential decreases of 1279 cases (95% UI: 798, 1876) or 18.6% (95% UI: 11.6%, 27.4%) per 100,000 women, and 1934 cases (95% UI: 1209, 2796) or 24.5% (95% UI: 15.3%; 35.4%) per 100,000 men; as well as in colorectal cancer cases, estimated to be 69 cases (95% UI: 40, 103) or 8.7% (95% UI: 5.1%, 13.0%) per 100,000 women, and 102 cases (95% UI: 50, 161) or 10.4% (95% UI: 5.0%, 16.4%) per 100,000 men. Increasing fruit consumption to more than 200 g/d could result in the biggest reduction in stroke cases, with potential decreases of 382 cases (95% UI: 240, 473) or 17.7% (95% UI: 11.1%, 21.9%) per 100,000 women, and 428 cases (95% UI: 273, 525) or 19.1% (95% UI: 12.2%, 23.4%) per 100,000 men. Increasing fruit consumption could also lead to the largest reduction in lung cancer cases, estimated at 15 cases (95% UI: 8, 23) or 8.2% (95% UI: 4.4%, 12.8%) per 100,000 women, and 20 cases (95% UI: 9, 33) or 7.2% (95% UI: 3.4%, 12.0%) per 100,000 men. Supplementary Table S3 shows the estimated preventable NCD cases per 100,000 population by 2030 and 2040. The estimated total numbers of preventable NCD cases by 2050 can be found in Supplementary Table S4.

**Table 2:** Projected number and percentage of preventable non-communicable disease (NCD) cases per 100,000 women or men (and 95% uncertainty intervals) in 2050 if the entire population adhered to components of the Dutch dietary guidelines.

Food groups/NCDs	Women		Men	
	Cases preventable	Percentage, %	Cases preventable	Percentage, %
**Fruit**				
Coronary heart disease	398 (85, 676)	8.5 (1.8, 14.5)	511 (110, 865)	8.3 (1.8, 14.1)
Stroke	382 (240, 473)	17.7 (11.1, 21.9)	428 (273, 525)	19.1 (12.2, 23.4)
Type 2 diabetes	443 (68, 753)	6.4 (1.0, 10.9)	499 (58, 868)	6.3 (0.7, 11.0)
Colorectal cancer	−6 (−8, −4)	−0.8 (−1.0, −0.5)	−17 (−22, −11)	−1.7 (−2.2, −1.1)
Lung cancer	15 (8, 23)	8.2 (4.4, 12.8)	20 (9, 33)	7.2 (3.4, 12.0)
**Vegetables**				
Coronary heart disease	338 (35, 532)	7.2 (0.7, 11.4)	436 (62, 677)	7.1 (1.0, 11.0)
Stroke	108 (44, 169)	5.0 (2.1, 7.8)	118 (53, 181)	5.3 (2.4, 8.0)
Type 2 diabetes	−19 (−28, −5)	−0.3 (−0.4, −0.1)	−43 (−65, −8)	−0.5 (−0.8, −0.1)
Colorectal cancer	14 (2, 25)	1.7 (0.3, 3.2)	13 (−1, 28)	1.3 (−0.1, 2.9)
Lung cancer	0 (0, 0)	−0.1 (−0.2, −0.1)	−1 (−2, 0)	−0.5 (−0.7, −0.1)
**Whole grains**				
Coronary heart disease	413 (235, 547)	8.9 (5.1, 11.8)	443 (256, 587)	7.2 (4.2, 9.6)
Stroke	228 (186, 269)	10.6 (8.7, 12.5)	217 (177, 253)	9.7 (7.9, 11.3)
Type 2 diabetes	502 (245, 693)	7.3 (3.6, 10.1)	474 (222, 661)	6.0 (2.8, 8.4)
Colorectal cancer	54 (41, 73)	6.8 (5.2, 9.3)	51 (38, 73)	5.2 (3.8, 7.3)
Lung cancer	0 (−1, 0)	−0.2 (−0.3, −0.2)	−2 (−3, −2)	−0.8 (−0.9, −0.6)
**Nuts/seeds**				
Coronary heart disease	698 (164, 1169)	15.0 (3.5, 25.0)	833 (224, 1381)	13.6 (3.7, 22.6)
Stroke	0 (−8, 8)	0 (−0.4, 0.4)	−10 (−26, 7)	−0.4 (−1.1, 0.3)
Type 2 diabetes	497 (272, 696)	7.2 (3.9, 10.1)	511 (260, 717)	6.5 (3.3, 9.1)
Colorectal cancer	−4 (−6, −1)	−0.5 (−0.7, −0.1)	−11 (−19, −2)	−1.1 (−1.9, −0.2)
Lung cancer	0 (0, 0)	−0.2 (−0.2, −0.1)	−2 (−4, 0)	−0.8 (−1.3, −0.2)
**Legumes**				
Coronary heart disease	97 (37, 156)	2.1 (0.8, 3.3)	130 (55, 203)	2.1 (0.9, 3.3)
Stroke	−1 (−2, 0)	0 (−0.1, 0)	−2 (−4, 0)	−0.1 (−0.2, 0)
Type 2 diabetes	−3 (−5, 0)	0 (−0.1, 0)	−8 (−15, −1)	−0.1 (−0.2, 0)
Colorectal cancer	0 (−1, 0)	0 (−0.1, 0)	−1 (−2, 0)	−0.1 (−0.2, 0)
Lung cancer	0 (0, 0)	0 (0, 0)	0 (0, 0)	−0.1 (−0.2, 0)
**Fish**				
Coronary heart disease	101 (73, 133)	2.2 (1.6, 2.8)	129 (95, 168)	2.1 (1.6, 2.7)
Stroke	27 (9, 47)	1.2 (0.4, 2.2)	28 (9, 49)	1.3 (0.4, 2.2)
Type 2 diabetes	−5 (−8, −3)	−0.1 (−0.1, 0)	−12 (−17, −9)	−0.2 (−0.2, −0.1)
Colorectal cancer	−1 (−1, 0)	−0.1 (−0.1, 0)	−2 (−2, −1)	−0.2 (−0.3, −0.1)
Lung cancer	0 (0, 0)	0 (0, 0)	0 (−1, 0)	−0.1 (−0.2, −0.1)
**Red meat**				
Coronary heart disease	−2 (−8, 3)	0 (−0.2, 0.1)	−10 (−24, 1)	−0.2 (−0.4, 0)
Stroke	24 (−5, 50)	1.1 (−0.2, 2.3)	35 (−8, 74)	1.6 (−0.3, 3.3)
Type 2 diabetes	141 (32, 249)	2.0 (0.5, 3.6)	241 (59, 420)	3.0 (0.7, 5.3)
Colorectal cancer	19 (7, 33)	2.4 (0.8, 4.2)	31 (10, 56)	3.1 (1.0, 5.7)
Lung cancer	8 (2, 15)	4.3 (0.9, 8.0)	17 (3, 31)	5.9 (1.2, 11.1)
**Processed meat**				
Coronary heart disease	859 (180, 1676)	18.5 (3.9, 36.2)	1457 (349, 2700)	23.8 (5.7, 44.1)
Stroke	184 (67, 289)	8.5 (3.1, 13.4)	256 (96, 407)	11.4 (4.3, 18.1)
Type 2 diabetes	1279 (798, 1876)	18.6 (11.6, 27.4)	1934 (1209, 2796)	24.5 (15.3, 35.4)
Colorectal cancer	69 (40, 103)	8.7 (5.1, 13.0)	102 (50, 161)	10.4 (5.0, 16.4)
Lung cancer	−1 (−1, 0)	−0.4 (−0.5, −0.2)	−6 (−9, −3)	−2.2 (−3.3, −1.2)
**Dairy products**				
Coronary heart disease	−2 (−3, −1)	0 (−0.1, 0)	−4 (−5, −3)	−0.1 (−0.1, 0)
Stroke	−1 (−2, −1)	−0.1 (−0.1, 0)	−2 (−2, −1)	−0.1 (−0.1, 0)
Type 2 diabetes	−3 (−4, −2)	0 (−0.1, 0)	−4 (−5, −3)	0 (−0.1, 0)
Colorectal cancer	28 (18, 38)	3.6 (2.3, 4.8)	32 (21, 44)	3.3 (2.1, 4.4)
Lung cancer	0 (0, 0)	0 (0, 0)	0 (0, 0)	−0.1 (−0.1, 0)
**Tea**				
Coronary heart disease	7 (−2, 16)	0.1 (0, 0.3)	5 (−9, 21)	0.1 (−0.1, 0.3)
Stroke	187 (86, 274)	8.7 (4.0, 12.7)	237 (119, 336)	10.5 (5.3, 15.0)
Type 2 diabetes	381 (167, 614)	5.5 (2.4, 8.9)	496 (236, 791)	6.3 (3.0, 10.0)
Colorectal cancer	−2 (−3, −1)	−0.2 (−0.4, −0.1)	−5 (−7, −2)	−0.5 (−0.7, −0.2)
Lung cancer	0 (0, 0)	−0.1 (−0.1, −0.1)	−1 (−1, −1)	−0.4 (−0.5, −0.2)
**Sugary beverages**				
Coronary heart disease	214 (−16, 422)	4.6 (−0.3, 9.1)	401 (−2, 755)	6.5 (0, 12.3)
Stroke	3 (−1, 6)	0.1 (0, 0.3)	2 (−7, 11)	0.1 (−0.3, 0.5)
Type 2 diabetes	339 (212, 452)	4.9 (3.1, 6.6)	565 (359, 752)	7.1 (4.5, 9.5)
Colorectal cancer	−1 (−2, 0)	−0.1 (−0.3, 0)	−4 (−8, 0)	−0.4 (−0.8, 0)
Lung cancer	0 (0, 0)	−0.1 (−0.1, 0)	−1 (−2, 0)	−0.3 (−0.6, 0)

Table 3 shows the projected gains in life expectancy and disease-free life expectancy at birth, age 50, and age 70 in 2050, which could be achieved by adhering to dietary guidelines. Eliminating the consumption of processed meat could yield the largest gains in total life expectancy at all ages; for example, at age 50, 0.310 (95% UI: 0.181, 0.453) years, or approximately 4 months, for women, and 0.724 (95% UI: 0.398, 1.092) years, or approximately 9 months, for men. It could also lead to the largest gains in disease-free life expectancy at all ages; for example, at age 50, 1.554 (95% UI: 1.032, 2.189) years, equivalent to 1 year and 7 months, for women, and 2.494 (95% UI: 1.627, 3.504) years, equivalent to 2 years and 6 months, for men. The second largest gains in life expectancy and disease-free life expectancy were found for fruit, followed by whole grains and nuts/seeds. In 2050 for 50-year-olds, increasing fruit consumption could increase total life expectancy by 0.263 (95% UI: 0.183, 0.325) years for women and 0.407 (95% UI: 0.269, 0.527) years for men, as well as disease-free life expectancy by 0.876 (95% UI: 0.548, 1.182) years for women and 1.038 (95% UI: 0.653, 1.419) years for men. The smallest gains in life expectancy were found for dairy products, followed by legumes and fish. The time trends for the projected gains in life expectancy and disease-free life expectancy over the 30-year simulation period up to 2050 are shown in Supplementary Figure S1. As simulation time increases, the gains in life expectancy and disease-free life expectancy increase.

**Table 3:** Projected gains in life expectancy and disease-free life expectancy in years (and 95% uncertainty intervals) at birth, age 50, and age 70 in 2050 if the entire population adhered to components of the Dutch dietary guidelines.

Food groups	Age	Gains in total life expectancy		Gains in disease-free life expectancy	
		Men	Women	Men	Women
Fruit	0	0.431 (0.292, 0.551)	0.286 (0.204, 0.350)	1.142 (0.736, 1.537)	0.960 (0.616, 1.278)
	50	0.407 (0.269, 0.527)	0.263 (0.183, 0.325)	1.038 (0.653, 1.419)	0.876 (0.548, 1.182)
	70	0.296 (0.177, 0.389)	0.165 (0.103, 0.212)	0.581 (0.326, 0.834)	0.497 (0.279, 0.705)
Vegetables	0	0.164 (0.049, 0.236)	0.095 (0.037, 0.129)	0.411 (0.140, 0.574)	0.325 (0.115, 0.456)
	50	0.153 (0.040, 0.224)	0.088 (0.031, 0.121)	0.379 (0.115, 0.536)	0.302 (0.096, 0.429)
	70	0.113 (0.020, 0.173)	0.065 (0.016, 0.095)	0.221 (0.043, 0.329)	0.192 (0.040, 0.286)
Whole grains	0	0.271 (0.210, 0.327)	0.203 (0.166, 0.239)	0.860 (0.674, 1.041)	0.868 (0.679, 1.051)
	50	0.255 (0.194, 0.310)	0.188 (0.152, 0.223)	0.779 (0.604, 0.952)	0.790 (0.610, 0.964)
	70	0.187 (0.135, 0.234)	0.139 (0.105, 0.170)	0.431 (0.319, 0.541)	0.475 (0.354, 0.597)
Nuts/seeds	0	0.272 (0.075, 0.436)	0.142 (0.045, 0.218)	0.951 (0.472, 1.303)	0.814 (0.411, 1.110)
	50	0.263 (0.067, 0.426)	0.139 (0.042, 0.215)	0.880 (0.412, 1.220)	0.765 (0.371, 1.056)
	70	0.214 (0.044, 0.354)	0.113 (0.030, 0.179)	0.523 (0.195, 0.765)	0.490 (0.194, 0.707)
Legumes	0	0.031 (0.008, 0.053)	0.014 (0.004, 0.024)	0.091 (0.039, 0.140)	0.066 (0.027, 0.103)
	50	0.029 (0.007, 0.051)	0.013 (0.003, 0.023)	0.083 (0.033, 0.131)	0.061 (0.023, 0.098)
	70	0.021 (0.002, 0.040)	0.010 (0.001, 0.019)	0.045 (0.010, 0.078)	0.036 (0.008, 0.063)
Fish	0	0.044 (0.032, 0.060)	0.024 (0.015, 0.034)	0.111 (0.087, 0.142)	0.087 (0.068, 0.114)
	50	0.042 (0.030, 0.058)	0.023 (0.014, 0.032)	0.104 (0.081, 0.135)	0.083 (0.064, 0.108)
	70	0.032 (0.022, 0.045)	0.017 (0.010, 0.026)	0.062 (0.046, 0.083)	0.054 (0.039, 0.073)
Red meat	0	0.125 (0.079, 0.181)	0.068 (0.042, 0.101)	0.272 (0.175, 0.408)	0.166 (0.106, 0.253)
	50	0.123 (0.077, 0.179)	0.066 (0.041, 0.098)	0.251 (0.160, 0.379)	0.158 (0.101, 0.242)
	70	0.085 (0.053, 0.127)	0.038 (0.024, 0.057)	0.142 (0.087, 0.220)	0.092 (0.052, 0.145)
Processed meat	0	0.742 (0.415, 1.112)	0.321 (0.190, 0.464)	2.685 (1.795, 3.720)	1.677 (1.142, 2.326)
	50	0.724 (0.398, 1.092)	0.310 (0.181, 0.453)	2.494 (1.627, 3.504)	1.554 (1.032, 2.189)
	70	0.559 (0.284, 0.869)	0.247 (0.133, 0.374)	1.430 (0.847, 2.106)	0.987 (0.594, 1.463)
Dairy products	0	0.019 (0.012, 0.026)	0.018 (0.012, 0.024)	0.025 (0.016, 0.034)	0.026 (0.017, 0.035)
	50	0.019 (0.012, 0.025)	0.018 (0.011, 0.024)	0.024 (0.016, 0.033)	0.025 (0.016, 0.034)
	70	0.013 (0.008, 0.017)	0.011 (0.007, 0.015)	0.018 (0.012, 0.024)	0.017 (0.011, 0.023)
Tea	0	0.128 (0.073, 0.178)	0.081 (0.044, 0.117)	0.504 (0.309, 0.682)	0.404 (0.235, 0.554)
	50	0.121 (0.066, 0.170)	0.075 (0.039, 0.110)	0.457 (0.272, 0.625)	0.371 (0.210, 0.513)
	70	0.094 (0.046, 0.136)	0.056 (0.025, 0.084)	0.252 (0.129, 0.361)	0.222 (0.108, 0.321)
Sugary beverages	0	0.131 (0.023, 0.221)	0.049 (0.012, 0.081)	0.665 (0.357, 0.941)	0.380 (0.208, 0.540)
	50	0.123 (0.019, 0.210)	0.046 (0.011, 0.077)	0.581 (0.296, 0.838)	0.335 (0.173, 0.485)
	70	0.087 (0.009, 0.153)	0.035 (0.006, 0.061)	0.276 (0.112, 0.423)	0.179 (0.072, 0.276)

## Discussion

Our results show that, compared with current food consumption levels, adhering to components of the Dutch dietary guidelines could substantially reduce cases of coronary heart disease, stroke, type 2 diabetes, colorectal cancer, and lung cancer in the Netherlands by 2050. This adherence could also increase total and disease-free life expectancy for the Dutch population. Among the food groups assessed, eliminating processed meat and increasing fruit consumption are projected to yield the largest reduction in NCD burden.

Previous studies have assessed the burden of NCDs across populations attributed to non-optimal food consumption.^2^^,^^6^, ^7^, ^8^ The GBD study has shown that low consumption of whole grains and fruit, and high consumption of processed meat are the leading dietary risk factors for NCD burden in high socio-demographic index countries.^2^^,^^8^ Another study by *Schwingshackl* et al. has estimated that in the Netherlands, sugary beverages, processed meat, red meat, and nuts/seeds were the main contributors to NCD burden. These four food groups accounted for approximately 30%, 35%, and 15% of total cases of coronary heart disease, type 2 diabetes, and colorectal cancer, respectively.^6^ Our results consolidate this evidence that non-optimal food consumption, especially processed meat and fruit, is among the major modifiable risk factors to prevent future NCDs in the Dutch population.

There is currently a lack of comprehensive assessment of the potential future long-term reductions in the NCD burden and gains in population health that could be achieved through dietary improvements. Our results, therefore, further extend the existing evidence. Specifically, eliminating processed meat consumption (0 g/d) could prevent the highest number of cases of coronary heart disease, type 2 diabetes, and colorectal cancer, and is projected to increase life expectancy by 0.310 (women) and 0.724 (men) years at age 50 in 2050. Increasing fruit consumption to more than 200 g/d could also result in substantial gains in future population health, followed by increasing the consumption of nuts/seeds to more than 15 g/d and whole grains to more than 90 g/d. Adhering to consumption recommendations for legumes (≥10 g/d) and dairy products (300–450 g/d) is projected to have the smallest effects in reducing NCD burden. While the consumption levels of the study population for all food groups assessed were not optimal, the small effects observed for legumes may be due to their moderate effect sizes on disease outcomes and the low recommended consumption levels in the dietary guidelines.^1^^,^^2^ Regarding dairy products, most of the Dutch population has already reached or exceeded the optimal consumption level, which may explain the limited effects. Moreover, the potential reductions in NCD burden achievable by eliminating the consumption of sugary beverages may be under-estimated, as the mediation effects via body weight was not accounted for, and not all health conditions (such as caries) were considered.^20^

Our results show that optimizing the consumption of certain food groups could lead to a small increase in the cases of some diseases (such as processed meat for lung cancer). This is mainly because eliminating one dietary risk factor—and thus reducing the burden of NCDs associated with it—would naturally reduce mortality and lead to a larger population; however, this may not directly affect the incidence of other NCDs not associated with the dietary risk factor removed. Consequently, cases of these other NCDs could naturally increase in a larger population with increased life expectancy.^12^ Nevertheless, as shown in Table 2 and Supplementary Table S4, such negative effects are minimal, especially considering that our analysis was conducted under an extreme scenario where the detrimental exposure of the food group under assessment was fully eliminated (according to dietary guidelines). Furthermore, it should be noted that the estimated effects across food groups in this study are not additive. The combined effects between food groups were not assessed, because the DYNAMO-HIA model is limited to analyzing one single risk factor. Additionally, when calculating disease burden, the RR estimates were obtained from meta-analyses of prospective observational studies. Whilst these studies controlled for major confounding factors (such as age, sex, energy intake, body mass index, and lifestyle factors), there remains a possibility of residual confounding. Potential sources of confounding include other dietary factors that are highly correlated with the food group assessed, as well as interactive effects between food groups. These factors may influence the epidemiological estimation of the RRs for food consumption on NCD endpoints, potentially leading to over- or under-estimation of the reductions in NCD burden related to the food group assessed.^2^^,^^21^ Importantly, the estimates of disease burden are derived from the population attributable fractions using RRs, which quantify the proportion of incident cases in the population attributed to a specific risk factor. While a disease can be prevented by eliminating multiple different risk factors, this explains the well-known fact that population attributable fractions can sum to more than 100%.^19^ Hence, the estimated reductions in disease burden for each food group may not be summed to represent the combined effects across food groups. Our results should be interpreted as estimates of the population health gains that could be achieved by eliminating the non-optimal consumption of a specific food group. A healthy diet is multidimensional and not limited to a single food group.^1^ Nevertheless, in our simulation process, consumption levels of other food groups were not specified and assumed to remain unchanged. Future studies are warranted to explore the effects of adhering to different healthy dietary patterns on reducing the population future NCD burden.

In the Netherlands, the persistently high burden of NCDs requires forward-thinking, evidence-based prioritization of prevention resources and health policies.^4^ Our results clearly show that eliminating the consumption of processed meat could lead to the largest reduction in NCD burden and the largest gains in life expectancy in future. Adhering to consumption recommendations for fruit, whole grains, and nuts/seeds could also considerably reduce NCD burden, with a similar level of impact. These food groups may be priority targets in strategies and policies to mitigate NCD burden and to improve population health.

Optimizing food consumption at the population level has been a longstanding public health challenge. Traditional strategies that mainly target individual behaviors by increasing awareness and knowledge have shown limited success. It is increasingly recognized that improving diet requires addressing a wide range of interconnected elements within the food system that go beyond the scope of conventional public health approaches.^22^, ^23^, ^24^, ^25^, ^26^ Measures such as imposing a meat tax, reducing fruit and vegetable taxes, and providing fruit and vegetable vouchers may be potential food system measures to optimize food consumption in the Netherlands and, consequently, reduce the population-level NCD burden.^25^, ^26^, ^27^, ^28^, ^29^, ^30^ Policies addressing food production and distribution should also be targeted.^25^^,^^31^ Moreover, current food consumption patterns impose a significant environmental impact.^32^ Reducing meat consumption and increasing consumption of plant-based protein sources (such as nuts/seeds and legumes) may synergistically improve population health and lessen environmental burdens.^32^^,^^33^ Given the substantial NCD burden related to food consumption and the limited success of current prevention strategies, joint actions addressing both public health and food system sectors are urgently needed. Policymakers should clearly recognize the significant health benefits for the population, along with the broader societal and environmental gains, that may result from optimizing food consumption.

This study is the first to comprehensively assess the potential long-term reductions in NCD burden and gains in population health that could be achieved by optimizing the consumption of eleven food groups according to dietary guidelines. By using nationally representative food consumption data, detailed demographic information, and data on NCD epidemiology in the Netherlands, the analysis is based on an accurate characterization of the population.

However, there are also several limitations that should be noted. First, we did not consider the potential influence of energy intake on population health. Adjusting for energy means that food consumption is defined as risks in terms of the share of the total diet but not as the absolute levels of exposure.^2^ Changes in the consumption level of one food group thus should be compensated by changes in the consumption of other food groups, while RR estimates concerning such types of substitution is generally unavailable. Second, we did not assess the direct effects of food consumption on mortality and survival in people with NCDs. We assumed that the impact of food consumption on mortality was fully realized by the presence of NCDs, taking into account comorbidities and competing risks between NCDs.^12^^,^^13^ It is biologically implausible that food consumption may directly influence mortality without the occurrence of NCDs. In this study, we focused on assessing the impact of food consumption on five NCDs, which are the leading causes of morbidity and mortality in the Netherlands.^4^^,^^5^ However, evidence also suggests that food consumption may be associated with other NCDs, such as chronic kidney diseases and certain types of cancer.^34^^,^^35^ Third, the time lag between changes of food consumption and their effects on health was not considered. Short-term changes in food consumption likely have weaker effects than long-term changes, and individual responses to dietary changes may vary.^36^ The RR estimates used in this analysis were obtained from meta-analyses of various observational cohort studies with differing follow-up durations and these studies generally lacked information on changes in food consumption before the baseline assessment.^2^ Fourth, the effects of food consumption on NCD burden were only investigated in adults, considering the effects of food consumption on NCD endpoints modeled in this study in children and adolescents have not been well established. Fifth, the input data used for estimates in this study cover different time periods. Using up-to-date input data could improve the quality of our estimates, but these data are not available. Finally, we did not consider the cohort effects on future changes in food consumption levels in the population. Future changes in environmental factors and advancements in medical treatment may add further uncertainties to our estimates.^37^

Building upon previous research that contributed to estimating the current NCD burden attributed to suboptimal food consumption,^2^^,^^6^ our study extends this evidence by providing projections that demonstrate how adhering to components of the Dutch dietary guidelines could substantially reduce the future NCD burden and increase life expectancy in the Dutch population by 2050. Specifically eliminating the consumption of processed meat, and increasing the consumption of fruit, nuts/seeds, and whole grains could yield the largest gains in population health, making them priority prevention targets. Taken together, these findings collectively underscore the importance of improving diets by adhering to dietary guidelines as a fundamental strategy for NCD prevention at the population level. Joint actions addressing both public health and food system sectors are needed for tackling a broad range of underlying factors to promote healthy food consumption.

## Contributors

MJD, MPP, and SB designed and conceptualized the study. MJD analyzed the data and drafted the manuscript. All authors contributed to the interpretation of data. MPP and SB contributed to the discussion and critically reviewed/edited the manuscript. All authors have full access to all the data and have verified the underlying data in the study. All authors have read and approved the final content of the manuscript and have final responsibility for the decision to submit for publication.

## Data sharing statement

Data from the Dutch National Food Consumption Survey 2012–2016 can be requested from the National Institute for Public Health and the Environment, the Netherlands (https://www.rivm.nl/en/dutch-national-food-consumption-survey/data-on-request). DYNAMO-HIA software and Dutch non-communicable disease epidemiology data are publicly available at https://www.dynamo-hia.eu/.

## Declaration of interests

Maartje P Poelman received expert consultation fee from the Dutch National Institute for Public Health and the Environment (RIVM) for the evaluation of the National Prevention Agreement. Maartje P Poelman was a workgroup member (unpaid) in shaping the national knowledge agenda (theme healthy living environment) of the Dutch Heart Foundation.
