# Supplementary material for: Non-communicable disease burden through adhering to Dutch dietary guidelines: a modeling study to estimate future reductions
Source: eClinicalMedicine. 2025 Mar 27;82:103170. doi: 10.1016/j.eclinm.2025.103170 (PMC11995777; doi:10.1016/j.eclinm.2025.103170)

**Non-Communicable Disease Burden through Adhering to Dutch Dietary Guidelines: a modeling study to estimate future reductions**

**Supplementary Files**

Ming-Jie Duan^1,a^, Maartje P Poelman^2^, Sander Biesbroek^1^

^1^ Division of Human Nutrition and Health, Wageningen University & Research, Wageningen, The Netherlands

^2^ Chair Group Consumption and Healthy Lifestyles, Wageningen University & Research, Wageningen, The Netherlands

^a^ Corresponding author, [frederick.duan@wur.nl](mailto:frederick.duan@wur.nl), Stippeneng 4, 6708 WE Wageningen, The Netherlands.

| **List of Supplementary Files** |  |
| --- | --- |
|  |  |
| **Supplementary Texts** |  |
| **Supplementary Text S1.** Recommended optimal consumption level for food groups according to the 2015 Dutch dietary guidelines | 3 |
| **Supplementary Text S2.** Explanations of excluding coffee, fats/oils, alcoholic beverages, and salt from the analysis | 5 |
| **Supplementary Text S3.** Search terms on PubMed to identify dose-response relative risk estimates from meta-analysis, dated July 1, 2024 | 6 |
| **Supplementary Text S4.** Calculation of age-specific relative risks of for associations between food groups and non-communicable diseases | 8 |
| **Supplementary Text S5.** Description of the technical core of DYNAMO-HIA model | 10 |
| **Supplementary Text S6.** Summary of input data used in this study using DYNAMO-HIA | 13 |
|  |  |
| **Supplementary Tables** |  |
| **Supplementary Table S1.** Categorization of food items in the Dutch National Food Consumption Survey | 14 |
| **Supplementary Table S2.** Summary of evidence and relative risk (RR) estimates for associations between food groups and non-communicable diseases | 15 |
| **Supplementary Table S3.** Projected number and percentage of preventable NCD cases per 100,000 women or men (and 95% Uncertainty Intervals [UIs]) in 2030 (a) and 2040 (b) if the entire population adhered to Dutch dietary guidelines | 28 |
| **Supplementary Table S4.** Projected number of total preventable NCD cases (and 95% Uncertainty Intervals [UIs]) by 2050 if the entire population adhered to Dutch dietary guidelines | 32 |
|  |  |
| **Supplementary Figures** |  |
| **Supplementary Figure S1.** Projected gains in life expectancy and disease-free life expectancy (and 95% Uncertainty Intervals [UIs]) at birth, and at ages 50 and 70 over a 30-year period (by 2050) if the entire population adhered to Dutch dietary guidelines | 35 |

**Supplementary Text S1.** Recommended optimal consumption level for food groups according to the 2015 Dutch dietary guidelines^1^

**1. Fruit and vegetables**

(1) Guideline: ≥ 200 g/day. Eat at least 200 grams of vegetables and at least 200 grams of fruit daily.

**2. Whole grains**

(1) Guideline: ≥ 90 g/day. Eat at least 90 grams of brown bread, whole-meal bread or other whole-grain products daily. Replace refined cereal products by whole-grain products.

(2) Consideration: the second sub-component of replacing refined grains by whole grains was not considered in the analysis. No quantitative recommendations or information were available about the consumption levels of refined grains associated with adverse health effects.^2^

**3. Nuts/seeds**

(1) Guideline: ≥ 15 g/day. Eat at least 15 grams of unsalted nuts daily.

**4. Legumes**

(1) Guideline: eat legumes weekly.

(2) Optimization: ≥ 10 g/day. In the development of the Dutch Healthy Diet Index (DHD15-index), one portion of legumes (60g) was divided by 7 and rounded to 10 g/day to obtain the daily recommended consumption level.^2^

**5. Fish**

(1) Guideline: eat one serving of fish, preferably oily fish weekly.

(2) Optimization: ≥ 15 g/day. In the development of the DHD15-index, one portion of fish (100g) was divided by 7 and rounded to obtain the daily recommended consumption level.^2^ The second sub-component of preferably eating oily fish was not considered in the analysis.

**6. Red meat and processed meat**

(1) Guideline: limit the consumption of red meat, particularly processed meat.

(2) Optimization: ≤ 45 g/day for red meat and 0 g/day for processed meat. In the Wheel of Five (Schijf van Vijf, visualized version of the Dutch dietary guidelines), the Netherlands Nutrition Center recommended to consume less than 300 grams of red meat weekly, which was approximated to 45 g/day.^2,3^ In DHD15-index, no consumption of processed meat was considered the optimal consumption level.^2^

**7. Dairy products**

(1) Guideline: take a few portions of dairy products daily, including milk or yogurt.

(2) Optimization: 300-450 g/day. In DHD15-index, two or three portions daily with a portion size of 150 grams (Netherlands Nutrition Center) was used, which led to the recommended consumption level of 300-450 g/day.^2^

**8. Tea**

(1) Guideline: drink three cups of tea daily.

(2) Optimization: ≥ 600 g/day. One cup of tea was approximated to 200 grams.

**9. Sugary beverages**

(1) Guideline: minimize the consumption of sugar-containing beverages.

(2) Optimization: 0 g/day. In DHD15-index, no consumption was considered the optimal consumption level.^2^

[1] Kromhout, D., Spaaij, C. J. K., de Goede, J., & Weggemans, R. M. (2016). The 2015 Dutch food-based dietary guidelines. European Journal of Clinical Nutrition, 70(8), 869-878.

[2] Looman, M., Feskens, E. J., de Rijk, M., et al. (2017). Development and evaluation of the Dutch Healthy Diet index 2015. Public Health Nutrition, 20(13), 2289-2299.

[3] Voedingscentrum. Gezond en duurzaam eten met de Schijf van Vijf. https://www.voedingscentrum.nl/nl/gezond-eten-met-de-schijf-van-vijf.aspx

**Supplementary Text S2.** Explanations of excluding coffee, fats/oils, alcoholic beverages, and salt from the analysis

**1. Coffee**

(1) Guideline: replace unfiltered coffee by filtered coffee.^1^

(2) Reason for exclusion: consumption data do not distinguish between filtered and unfiltered coffee.^2^

**2. Fats/oils**

(1) Guideline: replace butter, hard margarines and cooking fats with soft margarines, liquid cooking fats and vegetable oils.^1^

(2) Reasons for exclusion: no quantitative recommendation was given. There is a lack of relative risk estimates on replacing butter and hard margarines by soft/liquid cooking fats related to NCD outcomes.

**3. Alcoholic beverages**

(1) Guideline: do not drink alcohol or do not drink more than one glass daily.^1^

(2) Reason for exclusion: despite clear evidence supporting the detrimental effects of excessive alcohol consumption on health, the effects of moderate alcohol consumption on NCD outcomes remain inconclusive.^3,4^

**4. Salt**

(1) Guideline: limit salt intake to 6 g daily.^1^

(2) Reason for exclusion: there is no consumption data available on salt use.^2^

[1] Kromhout, D., Spaaij, C. J. K., de Goede, J., & Weggemans, R. M. (2016). The 2015 Dutch food-based dietary guidelines. European Journal of Clinical Nutrition, 70(8), 869-878.

[2] Van Rossum, C. T. M., Buurma, E. J. M., Vennemann, F. B. C., et al. (2017). Voedselconsumptie in 2012-2014 vergeleken met de Richtlijnen goede voeding 2015.

[3] Llamosas-Falcón, L., Rehm, J., Bright, S., et al. (2023). The Relationship Between Alcohol Consumption, BMI, and Type 2 Diabetes: A Systematic Review and Dose-Response Meta-analysis. Diabetes Care, 46(11), 2076-2083.

[4] Yoon, S. J., Jung, J. G., Lee, S., et al. (2020). The protective effect of alcohol consumption on the incidence of cardiovascular diseases: is it real? A systematic review and meta-analysis of studies conducted in community settings. BMC Public Health, 20, 1-9.

**Supplementary Text S3.** Search terms on PubMed to identify dose-response relative risk estimates from meta-analysis, dated July 1 2024

**1. Colorectal cancer**

(1) Vegetables: (meta*[Title]) AND ((vegetables[MeSH Terms]) OR (vegetable*[Title/Abstract])) AND ((cancer, colorectal[MeSH Terms]) OR (colorectal*[Title]))

Identified newest meta-analysis with dose-response RR estimates: Vieira, A. R., Abar, L., Chan, D. S. M., et al. (2017). Foods and beverages and colorectal cancer risk: a systematic review and meta-analysis of cohort studies, an update of the evidence of the WCRF-AICR Continuous Update Project. Annals of Oncology, 28(8), 1788-1802.

(2) Whole grains: (meta*[Title]) AND ((grains[MeSH Terms]) OR (grain*[Title/Abstract])) AND ((cancer, colorectal[MeSH Terms]) OR (colorectal*[Title]))

Identified newest meta-analysis with dose-response RR estimates: Vieira, A. R., Abar, L., Chan, D. S. M., et al. (2017). Foods and beverages and colorectal cancer risk: a systematic review and meta-analysis of cohort studies, an update of the evidence of the WCRF-AICR Continuous Update Project. Annals of Oncology, 28(8), 1788-1802.

(3) Dairy products: ((meta*[Title]) AND ((dairy products[MeSH Terms]) OR (dairy product[MeSH Terms]) OR (dairy*[Title/Abstract])) AND ((cancer, colorectal[MeSH Terms]) OR (colorectal*[Title])))

Identified newest meta-analysis with dose-response RR estimates: Jin, S., Kim, Y., & Je, Y. (2020). Dairy consumption and risks of colorectal cancer incidence and mortality: a meta-analysis of prospective cohort studies. Cancer Epidemiology, Biomarkers & Prevention, 29(11), 2309-2322.

**2. Stroke**

(1) Processed meat and red meat: ((meta*[Title]) AND ((meat[MeSH Terms]) OR (meat*[Title/Abstract])) AND ((stroke[MeSH Terms]) OR (stroke*[Title])))

Identified newest meta-analysis with dose-response RR estimates: Shi, W., Huang, X., Schooling, C. M., & Zhao, J. V. (2023). Red meat consumption, cardiovascular diseases, and diabetes: a systematic review and meta-analysis. European Heart Journal, 44(28), 2626-2635.

(2) Tea: ((meta*[Title]) AND ((tea[MeSH Terms]) OR (tea*[Title/Abstract]) OR (beverage[MeSH Terms])) AND ((stroke[MeSH Terms]) OR (stroke*[Title])))

Identified newest meta-analysis with dose-response RR estimates: Chung, M., Zhao, N., Wang, D., et al. (2020). Dose–response relation between tea consumption and risk of cardiovascular disease and all-cause mortality: a systematic review and meta-analysis of population-based studies. Advances in Nutrition, 11(4), 790-814.

(3) Fish: ((meta*[Title]) AND ((fish[MeSH Terms]) OR (fish*[Title/Abstract]) OR (disease, fish[MeSH Terms])) AND ((stroke[MeSH Terms]) OR (stroke*[Title])))

Identified newest meta-analysis with dose-response RR estimates: Zhao, W., Tang, H., Yang, X., et al. (2019). Fish consumption and stroke risk: a meta-analysis of prospective cohort studies. Journal of Stroke and Cerebrovascular Diseases, 28(3), 604-611.

**3. Lung cancer**

(1) Red meat: ((meta*[Title]) AND ((meat[MeSH Terms]) OR (meat*[Title/Abstract])) AND ((lung cancer[MeSH Terms]) OR (lung cancer*[Title/Abstract])))

Identified newest meta-analysis with dose-response RR estimates: Farvid, M. S., Sidahmed, E., Spence, N. D., et al. (2021). Consumption of red meat and processed meat and cancer incidence: a systematic review and meta-analysis of prospective studies. European Journal of Epidemiology, 36, 937-951.

**4. Coronary heart disease**

(1) Fish: ((meta*[Title]) AND ((fish[MeSH Terms]) OR (fish*[Title/Abstract]) OR (disease, fish[MeSH Terms])) AND ((coronary heart disease[MeSH Terms]) OR (disease, ischemic heart[MeSH Terms]) OR (heart*[Title])))

Identified newest meta-analysis with dose-response RR estimates: Zhang, B., Xiong, K., Cai, J., & Ma, A. (2020). Fish consumption and coronary heart disease: a meta-analysis. Nutrients, 12(8), 2278.

**5. Type 2 diabetes**

(1) Tea: ((meta*[Title]) AND ((tea[MeSH Terms]) OR (tea*[Title/Abstract]) OR (beverage[MeSH Terms])) AND ((diabetes[MeSH Terms]) OR (diabetes*[Title])))

Identified newest meta-analysis with dose-response RR estimates: Yang, W. S., Wang, W. Y., Fan, W. Y., Deng, Q., & Wang, X. (2014). Tea consumption and risk of type 2 diabetes: a dose–response meta-analysis of cohort studies. British Journal of Nutrition, 111(8), 1329-1339.

**Supplementary Text S4.** Calculation of age-specific relative risks of for associations between food groups and non-communicable diseases

In this study, the age-specific relative risks (RRs) were directly obtained from the GBD study when available. If RR estimates were not available from the GBD study, the RRs were obtained from the most up-to-date meta-analyses (see **Supplementary Text S3**). The age-specific RRs were then calculated using the established GBD methods, which considers the well-established age trends in the RRs of metabolic risk factors for cardiometabolic NCD outcomes (including coronary heart disease, stroke, and type 2 diabetes).^1,2^ In brief, the GBD study conducted a literature review to identify the most important metabolic mediators for each food group that could potentially influence the effects of food consumption on non-communicable disease (NCD) endpoints. The GBD study used the age trend in the RRs of these mediators and the NCD endpoints to estimate the age-specific RRs for each food group on NCD endpoints. The process involved the following steps, as described in the GBD study^1^:

1. The GBD study obtained the age-specific RRs for each mediator and NCD endpoint from a meta-analysis of pooled cohort studies.^2^

2. For each food group and NCD endpoint, the median age at event reported in the meta-analysis (used to obtain the RRs for food group-NCD endpoint) was estimated. The RR reported in the meta-analysis for the food group-NCD endpoint was assigned to the age group that included the median age at event.

3. The percentage change in the RRs between the age at event and other age groups was estimated for all relevant metabolic mediators, and the average of these estimates was calculated.

4. The average percentage change in the RRs for all relevant metabolic mediators was applied to the RR of the food group at the age at event, which served as the reference, to estimate the age-specific RRs for other age groups.

The identified metabolic mediators to estimate the age trend of effects of food consumption on cardiometabolic NCD outcomes by GBD study are summarized below^1^:

1. Processed meat: BMI, fasting plasma glucose, and systolic blood pressure.

2. Red meat: BMI and fasting plasma glucose.

3. Fish (seafood): BMI and systolic blood pressure.

4. Fruit: BMI, total serum cholesterol, fasting plasma glucose, and systolic blood pressure.

5. Vegetables: BMI, total serum cholesterol, fasting plasma glucose, and systolic blood pressure.

6. Legumes: BMI, total serum cholesterol, fasting plasma glucose, and systolic blood pressure.

7. Whole grains: BMI, total serum cholesterol, and fasting plasma glucose.

8. Nuts/seeds: BMI, total serum cholesterol, fasting plasma glucose, and systolic blood pressure.

In addition to the GBD summary, for Tea: BMI, fasting plasma glucose, total serum cholesterol, and systolic blood pressure.

[1] Afshin, A., Sur, P. J., Fay, K. A., et al. (2019). Health effects of dietary risks in 195 countries, 1990–2017: a systematic analysis for the Global Burden of Disease Study 2017. The Lancet, 393(10184), 1958-1972.

[2] Singh, G. M., Danaei, G., Farzadfar, F., et al. (2013). The age-specific quantitative effects of metabolic risk factors on cardiovascular diseases and diabetes: a pooled analysis. PLOS ONE, 8(7), e65174.

**Supplementary Text S5.** Description of the technical core of DYNAMO-HIA model

The following description of the technical core of DYNAMO-HIA model is obtained from the publications by Lhachimi et al [1] and Boshuizen et al [2]. These two publications provide an exhaustive level of detail on the technical aspects of DYNAMO-HIA.

DYNAMO-HIA is a health impact assessment tool that estimates how changes in risk factors, driven by programs, projects, or policies, affect multiple diseases and overall population health. It assumes that interventions lead to changes in risk-factor prevalence, which then influence disease incidence and related mortality. Using a counterfactual scenario approach, DYNAMO-HIA compares two scenarios: a reference scenario where risk factors remain constant and one or more alternative counterfactual scenarios where risk factors follow a presumed change. By projecting population health for both scenarios, the difference in population health over time between the reference and the counterfactual scenario thus represents the impact of changes in risk factors on future population health.

DYNAMO-HIA models a real-life population, divided by age (in one-year categories up to 95 years) and sex, without accounting for migration. The model works dynamically in one-year steps, projecting both reference and counterfactual scenarios over time. DYNAMO-HIA has explicit risk-factor states. Each individual in the simulation is assigned a specific risk-factor category, which is updated yearly based on age- and sex-specific probabilities. These risk-factor categories determine a person’s relative risk of developing a disease or dying. This approach ensures accurate and unbiased simulation estimates.

DYNAMO-HIA is a Markov-type model based on a multi-state model. It combines a stochastic micro-simulation to project risk-factor trajectories with a deterministic macro approach to calculate disease life tables. The micro-simulation module simulates a large number of unique risk-factor biographies, while the macro module constructs separate disease life tables for risk-factor biography, considering competing risks and multiple morbidity. These biography-specific life tables are updated for each birth cohort and for those already alive in the starting year, based on input data. The overall population outcomes are then obtained by aggregating the individual biography/diseases life tables. The total mortality is divided into mortality due to modeled diseases and mortality from other causes.

The figure below, as obtained from the publication by Lhachimi et al [1] illustrates disease clusters within disease life tables. More specifically, each disease cluster includes one or more non-communicable chronic diseases. Within a cluster, intermediate diseases can be defined, where one disease increases the risk of developing another (e.g., diabetes increasing the risk of coronary heart disease). For each disease, acutely fatal or curable fractions can also be specified. Disease clusters are assumed to act independently of one another in the model.


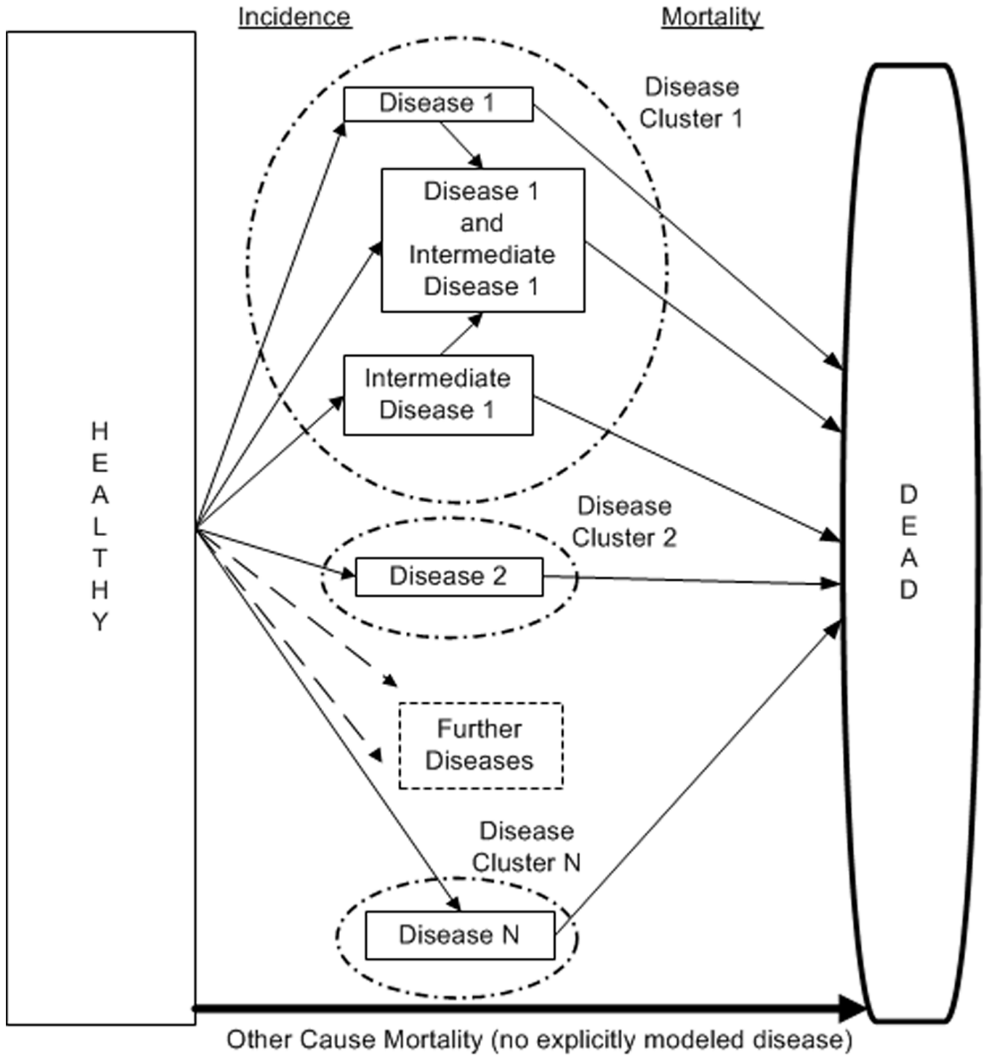


*This figure is obtained from the publication by Lhachimi et al [1].

The figure below, as obtained from the publication by Lhachimi et al [1] illustrates stylized cohort life tables with only one disease, three different biographies (normal weight, overweight, and obese as an example), and five timesteps. A disease life table is created for each risk-factor biography. The incidence of a disease is calculated as the baseline incidence multiplied by the relative risk of the individual’s risk-factor and disease status (in the case of an intermediate disease). The transition from healthy to death is calculated as the baseline other-cause mortality of the healthy multiplied by the relative risk of the given risk-factor status on other-cause mortality. The transition from diseased to death is calculated as the sum of the disease excess mortality and baseline other-cause mortality of the healthy, multiplied by the relative risk in the given-risk factor status. Remission is not explicitly modeled. Partially acutely fatal diseases are accounted for by specifying the fraction of incident cases that have immediate death.


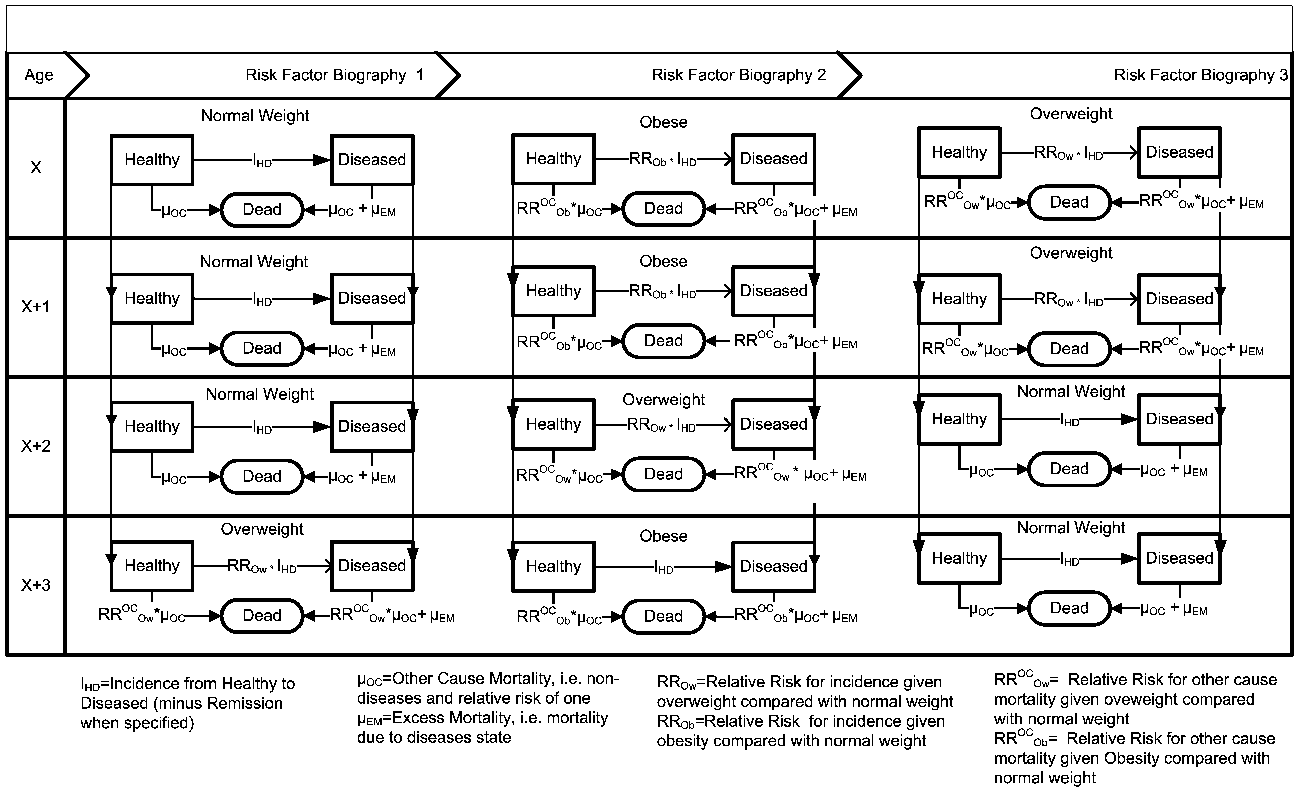


*This figure is obtained from the publication by Lhachimi et al [1].

In the publication by Boshuizen et al. [2], detailed calculation formulas are provided, including epidemiological models, a transition probability matrix for clusters of independent diseases, and methods for estimation using marginal data on risk factor and disease epidemiology for the initial state occupancy, as well as the parameters for the transition rates between states.

[1] Lhachimi, S. K., Nusselder, W. J., Smit, H. A., et al. (2012). DYNAMO-HIA–a dynamic modeling tool for generic health impact assessments. PLOS ONE, 7(5), e33317.

[2] Boshuizen, H. C., Lhachimi, S. K., van Baal, P. H., et al. (2012). The DYNAMO-HIA model: an efficient implementation of a risk factor/chronic disease Markov model for use in Health Impact Assessment (HIA). Demography, 49, 1259-1283.

**Supplementary Text S6.** Summary of input data used in this study using DYNAMO-HIA

1. Observed food consumption level: Dutch National Food Consumption Survey 2012-2016, which can be requested from the National Institute for Public Health and the Environment, the Netherlands (www.rivm.nl/en/dutch-national-food-consumption-survey/data-on-request).

2. Optimal food consumption level: the 2015 Dutch food-based dietary guidelines.^1^

3. Relative risk estimates of food consumption level on non-communicable disease (NCD) endpoints: GBD study and meta-analyses, see **Supplementary Table S2**.

4. NCD epidemiology data in the Netherlands: Data for coronary heart disease, stroke, and type 2 diabetes from 2011 were obtained from the Netherlands Information Network of General Practice. These data were further linked to the national mortality registry from the Statistics Netherlands (CBS) to derive data on disease-related mortality (opendata.cbs.nl). For coronary heart disease and stroke, data from hospital records (1995-2010) were also used to identify acute incident cases. Mortality for coronary heart disease and stroke was split into short-term (less than 1 year) and long-term mortality, with acute mortality derived by subtracting long-term mortality from mortality over one year. Data on lung cancer and colorectal cancer (1989-2011) were obtained from the Netherlands Cancer Registry (www.ikcnet.nl). Poisson regression models (including time trends) were used to estimate age- and sex-specific incidence rates and mortality in 2011.^2-4^ These data were processed by the DYNAMO-HIA consortium from the RIVM (Dutch National Institute for Public Health and the Environment), which is openly available from the DYNAMO-HIA website (www.dynamo-hia.eu/en/reference-data).

5. Dutch population data: CBS open data (opendata.cbs.nl).

[1] Kromhout, D., Spaaij, C. J. K., de Goede, J., & Weggemans, R. M. (2016). The 2015 Dutch food-based dietary guidelines. European Journal of Clinical Nutrition, 70(8), 869-878.

[2] Boshuizen, H. C., Lhachimi, S. K., van Baal, P. H., et al. (2012). The DYNAMO-HIA model: an efficient implementation of a risk factor/chronic disease Markov model for use in Health Impact Assessment (HIA). Demography, 49, 1259-1283.

[3] Van Oostrom, S. H., Picavet, H. S. J., Van Gelder, B. M., et al. (2012). Multimorbidity and comorbidity in the Dutch population–data from general practices. BMC Public Health, 12, 1-9.

[4] Boshuizen, H. C., Nusselder, W. J., Plasmans, M. H., et al. (2017). Taking multi-morbidity into account when attributing DALYs to risk factors: comparing dynamic modeling with the GBD2010 calculation method. BMC Public Health, 17, 1-13.

**Supplementary Table S1.** Categorization of food items in the Dutch National Food Consumption Survey

| **Food groups** | **Food items included** |
| --- | --- |
| Fruit | All types of fresh fruit (excluding dried fruit), e.g., apples, bananas, cranberries, figs, grapes, kiwi fruit, mangos, orange, papayas, pears, plums, strawberries. |
| Vegetables | All types of vegetables, e.g., asparagus, Brussels sprouts, carrots, cucumber, endives, eggplants, lettuce, leek, kale, onions, parsnips, sweet peppers, spinach, tomatoes, broad beans, sugar peas, mixed vegetables, mixed salad, . |
| Whole grains | Brown bread, whole grain bread, brown baguette, barleys, rye bread, cereal porridge/mix, whole grain crispbread, buckwheat, bulgar, cornmeal, whole grain flour, millet, muesli, oatmeal, whole grain pasta, brown rice, rye, whole grain wraps, quinoa, etc. |
| Nuts/seeds | All types of unsalted nuts/seeds, e.g., almonds, hazelnuts, cashew nuts, peanuts, Brazilian nuts, Macadamia’s, pine nuts, walnuts, sesame seeds. |
| Legumes | Black beans, brown beans, kidney beans, soy beans, mung beans, white beans, chickpeas, lentils, marrowfat peas, split peas, tofu, tempeh, etc. (Green peas, sugar peas, broad beans, and butter beans belong to the green vegetables.) |
| Fish | All types of fish and seafood, e.g., anchovies, cod, crabs, haddocks, lobsters, mackerels, mussels, pangasius, prawns, salmon, tilapia, shrimps, squids. |
| Red meat | All types of red meat, e.g., beef, horse meat, lambs/mutton, pork, veal. |
| Processed meat | Bacons, smoked beef, chicken nuggets, ham, burgers, liver pate, sausages, luncheon meat, fricandeau, shoarma, salami, sandwich meat, pork rolls (slavink), minced meat, Schnitzels, frikandel, viandel, beef tartare, meat balls, etc. |
| Dairy products | All types of dairy products, e.g., milk, yogurt (natural, fruit, yogurt drink, etc.), butter milk, fromage, cream, all types of cheese, custard, ice creams, dairy-based porridges, milk from powder, dairy-based desserts, sour cream, milkshake. |
| Tea | All types of tea (excluding ice tea), e.g., (flavored) black tea, (flavored) green tea, herbal tea. |
| Sugary beverages | All types of sugary beverages, e.g., cola, lemonade, sport drinks, whey drinks, juice, fruit drinks, ice tea, energy drink, alcohol-free beers. |

**Supplementary Table S2.** Summary of evidence and relative risk (RR) estimates for associations between food groups and non-communicable diseases (NCDs)

| **Dietary factors** | **Optimal consumption level (g/day)** | **NCD outcomes^a^** | **Source of RR** |
| --- | --- | --- | --- |
| Fruits (a) | ≥ 200 | Coronary heart disease | Afshin A, et al. (2019)^1^ |
|  |  | Stroke |  |
|  |  | Type 2 diabetes |  |
|  |  | Lung cancer |  |
| Vegetables (b) | ≥ 200 | Coronary heart disease | Afshin A, et al. (2019)^1^ |
|  |  | Stroke |  |
|  |  | Colorectal cancer | Vieira AR, et al. (2017)^2^ |
| Legumes (c) | ≥ 10 | Coronary heart disease | Afshin A, et al. (2019)^1^ |
| Whole grains (d) | ≥ 90 | Coronary heart disease | Afshin A, et al. (2019)^1^ |
|  |  | Stroke |  |
|  |  | Type 2 diabetes |  |
|  |  | Colorectal cancer | Vieira AR, et al. (2017)^2^ |
| Nuts/seeds (e) | ≥ 15 | Coronary heart disease | Afshin A, et al. (2019)^1^ |
|  |  | Type 2 diabetes |  |
| Dairy products (f) | 300-450 | Colorectal cancer | Jin S, et al. (2020)^3^ |
| Processed meat (g) | 0 | Coronary heart disease | Afshin A, et al. (2019)^1^ |
|  |  | Type 2 diabetes |  |
|  |  | Colorectal cancer |  |
|  |  | Stroke | Shi W, et al. (2023)^4^ |
| Red meat (h) | ≤ 45 | Type 2 diabetes | Afshin A, et al. (2019)^1^ |
|  |  | Colorectal cancer |  |
|  |  | Stroke | Shi W, et al. (2023)^4^ |
|  |  | Lung cancer | Farvid MS, et al. (2021)^5^ |
| Sugary beverages (i) | 0 | Coronary heart disease | Afshin A, et al. (2019)^1^ |
|  |  | Type 2 diabetes |  |
| Fish (j) | ≥ 15 | Coronary heart disease | Zhang B, et al. (2020)^6^ |
|  |  | Stroke | Zhao W, et al. (2019)^7^ |
| Tea (k) | ≥ 600 | Type 2 diabetes | Yang WS, et al. (2014)^8^ |
|  |  | Stroke | Chung M, et al. (2020)^9^ |

^a^ When RR estimates for total stroke is not available, estimates for ischemic stroke was used.

[1] Afshin, A., Sur, P. J., Fay, K. A., et al. (2019). Health effects of dietary risks in 195 countries, 1990–2017: a systematic analysis for the Global Burden of Disease Study 2017. The Lancet, 393(10184), 1958-1972. https://doi.org/10.1016/S0140-6736(19)30041-8

[2] Vieira, A. R., Abar, L., Chan, D. S. M., et al. (2017). Foods and beverages and colorectal cancer risk: a systematic review and meta-analysis of cohort studies, an update of the evidence of the WCRF-AICR Continuous Update Project. Annals of Oncology, 28(8), 1788-1802. https://doi.org/10.1093/annonc/mdx171

[3] Jin, S., Kim, Y., & Je, Y. (2020). Dairy consumption and risks of colorectal cancer incidence and mortality: a meta-analysis of prospective cohort studies. Cancer Epidemiology, Biomarkers & Prevention, 29(11), 2309-2322. https://doi.org/10.1158/1055-9965.EPI-20-0127

[4] Shi, W., Huang, X., Schooling, C. M., & Zhao, J. V. (2023). Red meat consumption, cardiovascular diseases, and diabetes: a systematic review and meta-analysis. European Heart Journal, 44(28), 2626-2635. https://doi.org/10.1093/eurheartj/ehad336

[5] Farvid, M. S., Sidahmed, E., Spence, N. D., Mante Angua, K., Rosner, B. A., & Barnett, J. B. (2021). Consumption of red meat and processed meat and cancer incidence: a systematic review and meta-analysis of prospective studies. European Journal of Epidemiology, 36, 937-951. https://doi.org/10.1007/s10654-021-00741-9

[6] Zhang, B., Xiong, K., Cai, J., & Ma, A. (2020). Fish consumption and coronary heart disease: a meta-analysis. Nutrients, 12(8), 2278. https://doi.org/10.3390/nu12082278

[7] Zhao, W., Tang, H., Yang, X., et al. (2019). Fish consumption and stroke risk: a meta-analysis of prospective cohort studies. Journal of Stroke and Cerebrovascular Diseases, 28(3), 604-611. https://doi.org/10.1016/j.jstrokecerebrovasdis.2018.10.036

[8] Yang, W. S., Wang, W. Y., Fan, W. Y., Deng, Q., & Wang, X. (2014). Tea consumption and risk of type 2 diabetes: a dose–response meta-analysis of cohort studies. British Journal of Nutrition, 111(8), 1329-1339. https://doi.org/10.1017/S0007114513003887

[9] Chung, M., Zhao, N., Wang, D., et al. (2020). Dose–response relation between tea consumption and risk of cardiovascular disease and all-cause mortality: a systematic review and meta-analysis of population-based studies. Advances in Nutrition, 11(4), 790-814. https://doi.org/10.1093/advances/nmaa010

**(a)** Fruit, relative risks per age groups

| Age groups (years) | Coronary heart disease | Stroke | Type 2 diabetes | Lung cancer |
| --- | --- | --- | --- | --- |
| 25-29 | 1.254 (1.083,1.442) | 2.024 (1.465,2.818) | 1.125 (1.027,1.238) | 1.076 (1.031,1.123) |
| 30-34 | 1.209 (1.070,1.361) | 1.834 (1.390,2.444) | 1.122 (1.026,1.232) | 1.076 (1.031,1.123) |
| 35-39 | 1.159 (1.054,1.271) | 1.621 (1.301,2.043) | 1.119 (1.026,1.226) | 1.076 (1.031,1.123) |
| 40-44 | 1.131 (1.045,1.221) | 1.480 (1.239,1.787) | 1.113 (1.024,1.214) | 1.076 (1.031,1.123) |
| 45-49 | 1.125 (1.043,1.211) | 1.403 (1.204,1.653) | 1.102 (1.022,1.194) | 1.076 (1.031,1.123) |
| 50-54 | 1.114 (1.039,1.193) | 1.333 (1.171,1.533) | 1.093 (1.020,1.176) | 1.076 (1.031,1.123) |
| 55-59 | 1.099 (1.034,1.167) | 1.272 (1.142,1.432) | 1.085 (1.019,1.160) | 1.076 (1.031,1.123) |
| 60-64 | 1.087 (1.030,1.146) | 1.222 (1.116,1.348) | 1.076 (1.017,1.143) | 1.076 (1.031,1.123) |
| 65-69 | 1.078 (1.027,1.130) | 1.181 (1.096,1.283) | 1.068 (1.015,1.128) | 1.076 (1.031,1.123) |
| 70-74 | 1.070 (1.025,1.117) | 1.145 (1.078,1.225) | 1.061 (1.014,1.114) | 1.076 (1.031,1.123) |
| 75-79 | 1.064 (1.022,1.106) | 1.114 (1.061,1.175) | 1.052 (1.012,1.098) | 1.076 (1.031,1.123) |
| 80-84 | 1.057 (1.020,1.095) | 1.054 (1.029,1.082) | 1.036 (1.008,1.066) | 1.076 (1.031,1.123) |
| 85-89 | 1.057 (1.020,1.095) | 1.054 (1.029,1.082) | 1.036 (1.008,1.066) | 1.076 (1.031,1.123) |
| >90 | 1.057 (1.020,1.095) | 1.054 (1.029,1.082) | 1.036 (1.008,1.066) | 1.076 (1.031,1.123) |

**Unit of RR:** per 100g/day

**Source of RR:** Afshin, A., Sur, P. J., Fay, K. A., et al. (2019). Health effects of dietary risks in 195 countries, 1990–2017: a systematic analysis for the Global Burden of Disease Study 2017. The Lancet, 393(10184), 1958-1972. https://doi.org/10.1016/S0140-6736(19)30041-8

**(b)** Vegetables, relative risks per age groups

| Age groups (years) | Coronary heart disease | Stroke | Colorectal cancer |
| --- | --- | --- | --- |
| 25-29 | 1.249 (1.089,1.446) | 1.249 (1.049,1.463) | 1.020 (1.010,1.042) |
| 30-34 | 1.205 (1.074,1.362) | 1.211 (1.042,1.388) | 1.020 (1.010,1.042) |
| 35-39 | 1.154 (1.057,1.269) | 1.165 (1.033,1.300) | 1.020 (1.010,1.042) |
| 40-44 | 1.126 (1.047,1.219) | 1.132 (1.027,1.238) | 1.020 (1.010,1.042) |
| 45-49 | 1.121 (1.045,1.210) | 1.113 (1.023,1.203) | 1.020 (1.010,1.042) |
| 50-54 | 1.111 (1.042,1.193) | 1.095 (1.020,1.170) | 1.020 (1.010,1.042) |
| 55-59 | 1.098 (1.037,1.168) | 1.079 (1.017,1.141) | 1.020 (1.010,1.042) |
| 60-64 | 1.086 (1.032,1.148) | 1.065 (1.014,1.116) | 1.020 (1.010,1.042) |
| 65-69 | 1.077 (1.029,1.133) | 1.054 (1.012,1.096) | 1.020 (1.010,1.042) |
| 70-74 | 1.070 (1.027,1.120) | 1.044 (1.009,1.077) | 1.020 (1.010,1.042) |
| 75-79 | 1.064 (1.024,1.109) | 1.035 (1.007,1.061) | 1.020 (1.010,1.042) |
| 80-84 | 1.057 (1.022,1.097) | 1.017 (1.004,1.029) | 1.020 (1.010,1.042) |
| 85-89 | 1.057 (1.022,1.097) | 1.017 (1.004,1.029) | 1.020 (1.010,1.042) |
| >90 | 1.057 (1.022,1.097) | 1.017 (1.004,1.029) | 1.020 (1.010,1.042) |

**Unit of RR:** per 100g/day

**Source of RR:** (1) coronary heart disease and stroke: Afshin, A., Sur, P. J., Fay, K. A., et al. (2019). Health effects of dietary risks in 195 countries, 1990–2017: a systematic analysis for the Global Burden of Disease Study 2017. The Lancet, 393(10184), 1958-1972. https://doi.org/10.1016/S0140-6736(19)30041-8; (2) colorectal cancer: Vieira, A. R., Abar, L., Chan, D. S. M., et al. (2017). Foods and beverages and colorectal cancer risk: a systematic review and meta-analysis of cohort studies, an update of the evidence of the WCRF-AICR Continuous Update Project. Annals of Oncology, 28(8), 1788-1802. https://doi.org/10.1093/annonc/mdx171

**(c)** Legumes, relative risks per age groups

| Age groups (years) | Coronary heart disease |
| --- | --- |
| 25-29 | 1.499 (1.180,1.890) |
| 30-34 | 1.453 (1.166,1.801) |
| 35-39 | 1.388 (1.144,1.677) |
| 40-44 | 1.332 (1.125,1.573) |
| 45-49 | 1.287 (1.110,1.490) |
| 50-54 | 1.237 (1.092,1.401) |
| 55-59 | 1.181 (1.071,1.303) |
| 60-64 | 1.139 (1.055,1.230) |
| 65-69 | 1.111 (1.045,1.183) |
| 70-74 | 1.089 (1.036,1.146) |
| 75-79 | 1.074 (1.030,1.120) |
| 80-84 | 1.101 (1.041,1.165) |
| 85-89 | 1.101 (1.041,1.165) |
| >90 | 1.101 (1.041,1.165) |

**Unit of RR:** per 50g/day

**Source of RR:** Afshin, A., Sur, P. J., Fay, K. A., et al. (2019). Health effects of dietary risks in 195 countries, 1990–2017: a systematic analysis for the Global Burden of Disease Study 2017. The Lancet, 393(10184), 1958-1972. https://doi.org/10.1016/S0140-6736(19)30041-8

**(d)** Whole grains, relative risks per age groups

| Age groups (years) | Coronary heart disease | Stroke | Type 2 diabetes | Colorectal cancer |
| --- | --- | --- | --- | --- |
| 25-29 | 1.478 (1.274,1.722) | 2.075 (1.669,2.517) | 1.231 (1.125,1.349) | 1.109 (1.067,1.140) |
| 30-34 | 1.387 (1.225,1.578) | 1.863 (1.548,2.199) | 1.226 (1.122,1.341) | 1.109 (1.067,1.140) |
| 35-39 | 1.285 (1.168,1.418) | 1.624 (1.406,1.849) | 1.220 (1.119,1.331) | 1.109 (1.067,1.140) |
| 40-44 | 1.228 (1.136,1.333) | 1.466 (1.309,1.625) | 1.208 (1.113,1.313) | 1.109 (1.067,1.140) |
| 45-49 | 1.216 (1.129,1.313) | 1.380 (1.255,1.505) | 1.189 (1.103,1.283) | 1.109 (1.067,1.140) |
| 50-54 | 1.194 (1.117,1.281) | 1.304 (1.206,1.401) | 1.172 (1.094,1.256) | 1.109 (1.067,1.140) |
| 55-59 | 1.165 (1.100,1.238) | 1.241 (1.165,1.316) | 1.156 (1.085,1.232) | 1.109 (1.067,1.140) |
| 60-64 | 1.141 (1.086,1.203) | 1.189 (1.130,1.247) | 1.139 (1.077,1.207) | 1.109 (1.067,1.140) |
| 65-69 | 1.125 (1.076,1.179) | 1.150 (1.104,1.195) | 1.125 (1.069,1.184) | 1.109 (1.067,1.140) |
| 70-74 | 1.112 (1.068,1.160) | 1.117 (1.081,1.151) | 1.111 (1.061,1.163) | 1.109 (1.067,1.140) |
| 75-79 | 1.102 (1.062,1.145) | 1.090 (1.063,1.116) | 1.095 (1.053,1.140) | 1.109 (1.067,1.140) |
| 80-84 | 1.097 (1.059,1.138) | 1.041 (1.029,1.053) | 1.064 (1.036,1.094) | 1.109 (1.067,1.140) |
| 85-89 | 1.097 (1.059,1.138) | 1.041 (1.029,1.053) | 1.064 (1.036,1.094) | 1.109 (1.067,1.140) |
| >90 | 1.097 (1.059,1.138) | 1.041 (1.029,1.053) | 1.064 (1.036,1.094) | 1.109 (1.067,1.140) |

**Unit of RR:** per 50g/day. The recommended consumption level is ≥ 90g/day. In analysis, the relative risks of consumption between 90-100 g/day were approximated to the relative risks of consumption ≥ 100g/day.

**Source of RR:** (1) coronary heart disease, stroke, and type 2 diabetes: Afshin, A., Sur, P. J., Fay, K. A., et al. (2019). Health effects of dietary risks in 195 countries, 1990–2017: a systematic analysis for the Global Burden of Disease Study 2017. The Lancet, 393(10184), 1958-1972. https://doi.org/10.1016/S0140-6736(19)30041-8; (2) colorectal cancer: Vieira, A. R., Abar, L., Chan, D. S. M., et al. (2017). Foods and beverages and colorectal cancer risk: a systematic review and meta-analysis of cohort studies, an update of the evidence of the WCRF-AICR Continuous Update Project. Annals of Oncology, 28(8), 1788-1802. https://doi.org/10.1093/annonc/mdx171

**(e)** Nuts/seeds, relative risks per age groups

| Age groups (years) | Coronary heart disease | Type 2 diabetes |
| --- | --- | --- |
| 25-29 | 1.176 (1.055,1.322) | 1.050 (1.025,1.075) |
| 30-34 | 1.143 (1.045,1.259) | 1.049 (1.025,1.073) |
| 35-39 | 1.105 (1.033,1.188) | 1.048 (1.024,1.071) |
| 40-44 | 1.084 (1.027,1.150) | 1.045 (1.023,1.068) |
| 45-49 | 1.081 (1.026,1.144) | 1.041 (1.021,1.062) |
| 50-54 | 1.074 (1.024,1.132) | 1.038 (1.019,1.056) |
| 55-59 | 1.064 (1.021,1.114) | 1.035 (1.018,1.052) |
| 60-64 | 1.056 (1.018,1.099) | 1.031 (1.016,1.046) |
| 65-69 | 1.050 (1.016,1.089) | 1.028 (1.014,1.042) |
| 70-74 | 1.046 (1.015,1.081) | 1.025 (1.013,1.037) |
| 75-79 | 1.042 (1.014,1.075) | 1.022 (1.011,1.032) |
| 80-84 | 1.039 (1.013,1.069) | 1.015 (1.007,1.022) |
| 85-89 | 1.039 (1.013,1.069) | 1.015 (1.007,1.022) |
| >90 | 1.039 (1.013,1.069) | 1.015 (1.007,1.022) |

**Unit of RR:** per 4.05g/day. The recommended consumption level is ≥ 15g/day. In analysis, the relative risks of consumption between 15-16 g/day were approximated to the relative risks of consumption ≥ 16g/day.

**Source of RR:** Afshin, A., Sur, P. J., Fay, K. A., et al. (2019). Health effects of dietary risks in 195 countries, 1990–2017: a systematic analysis for the Global Burden of Disease Study 2017. The Lancet, 393(10184), 1958-1972. https://doi.org/10.1016/S0140-6736(19)30041-8

**(f)** Dairy products, relative risks per age groups

| Age groups (years) | Colorectal cancer |
| --- | --- |
| 25-29 | 1.038 (1.027,1.054) |
| 30-34 | 1.038 (1.027,1.054) |
| 35-39 | 1.038 (1.027,1.054) |
| 40-44 | 1.038 (1.027,1.054) |
| 45-49 | 1.038 (1.027,1.054) |
| 50-54 | 1.038 (1.027,1.054) |
| 55-59 | 1.038 (1.027,1.054) |
| 60-64 | 1.038 (1.027,1.054) |
| 65-69 | 1.038 (1.027,1.054) |
| 70-74 | 1.038 (1.027,1.054) |
| 75-79 | 1.038 (1.027,1.054) |
| 80-84 | 1.038 (1.027,1.054) |
| 85-89 | 1.038 (1.027,1.054) |
| >90 | 1.038 (1.027,1.054) |

**Unit of RR:** per 100g/day. The recommended consumption level is 300-450 g/day. In analysis, the optimal consumption level of ≥ 300 g/day was used. The health effects of higher consumption exceeding the recommended consumption level (> 450 g/day) was not considered, because dose-response meta-analysis shows no clear detrimental health effects beyond this consumption level. Cheese was included in dairy products without conversion.

**Source of RR:** Jin, S., Kim, Y., & Je, Y. (2020). Dairy consumption and risks of colorectal cancer incidence and mortality: a meta-analysis of prospective cohort studies. Cancer Epidemiology, Biomarkers & Prevention, 29(11), 2309-2322. https://doi.org/10.1158/1055-9965.EPI-20-0127

**(g)** Processed meat, relative risks per age groups

| Age groups (years) | Coronary heart disease | Stroke | Type 2 diabetes | Colorectal cancer |
| --- | --- | --- | --- | --- |
| 25-29 | 2.568 (1.047,4.657) | 1.304 (1.073,1.577) | 1.940 (1.395,2.545) | 1.179 (1.093,1.267) |
| 30-34 | 2.124 (1.038,3.478) | 1.304 (1.073,1.577) | 1.913 (1.386,2.496) | 1.179 (1.093,1.267) |
| 35-39 | 1.720 (1.028,2.489) | 1.304 (1.073,1.577) | 1.881 (1.375,2.439) | 1.179 (1.093,1.267) |
| 40-44 | 1.545 (1.022,2.093) | 1.304 (1.073,1.577) | 1.824 (1.354,2.337) | 1.179 (1.093,1.267) |
| 45-49 | 1.547 (1.022,2.097) | 1.249 (1.061,1.465) | 1.731 (1.319,2.173) | 1.179 (1.093,1.267) |
| 50-54 | 1.520 (1.022,2.037) | 1.249 (1.061,1.465) | 1.653 (1.289,2.038) | 1.179 (1.093,1.267) |
| 55-59 | 1.467 (1.020,1.922) | 1.200 (1.049,1.368) | 1.583 (1.261,1.918) | 1.179 (1.093,1.267) |
| 60-64 | 1.422 (1.018,1.826) | 1.200 (1.049,1.368) | 1.512 (1.233,1.798) | 1.179 (1.093,1.267) |
| 65-69 | 1.386 (1.017,1.750) | 1.160 (1.040,1.290) | 1.450 (1.207,1.696) | 1.179 (1.093,1.267) |
| 70-74 | 1.354 (1.016,1.683) | 1.160 (1.040,1.290) | 1.393 (1.183,1.603) | 1.179 (1.093,1.267) |
| 75-79 | 1.325 (1.015,1.622) | 1.106 (1.027,1.189) | 1.332 (1.157,1.505) | 1.179 (1.093,1.267) |
| 80-84 | 1.252 (1.012,1.475) | 1.106 (1.027,1.189) | 1.216 (1.105,1.323) | 1.179 (1.093,1.267) |
| 85-89 | 1.252 (1.012,1.475) | 1.042 (1.011,1.074) | 1.216 (1.105,1.323) | 1.179 (1.093,1.267) |
| >90 | 1.252 (1.012,1.475) | 1.042 (1.011,1.074) | 1.216 (1.105,1.323) | 1.179 (1.093,1.267) |

**Unit of RR:** per 50g/day. The recommended consumption level is 0 g/day. In analysis, consumption ≤3 g/day was considered minimal tolerable optimal consumption with no excessive risks.

**Source of RR:** (1) coronary heart disease, type 2 diabetes, and colorectal cancer: Afshin, A., Sur, P. J., Fay, K. A., et al. (2019). Health effects of dietary risks in 195 countries, 1990–2017: a systematic analysis for the Global Burden of Disease Study 2017. The Lancet, 393(10184), 1958-1972. https://doi.org/10.1016/S0140-6736(19)30041-8; (2) stroke: Shi, W., Huang, X., Schooling, C. M., & Zhao, J. V. (2023). Red meat consumption, cardiovascular diseases, and diabetes: a systematic review and meta-analysis. European Heart Journal, 44(28), 2626-2635. https://doi.org/10.1093/eurheartj/ehad336.

**(h)** Red meat, relative risks per age groups

| Age groups (years) | Stroke | Type 2 diabetes | Colorectal cancer | Lung cancer |
| --- | --- | --- | --- | --- |
| 25-29 | 1.148 (0.982,1.344) | 1.322 (1.037,1.603) | 1.167 (1.033,1.309) | 1.290 (1.040,1.600) |
| 30-34 | 1.148 (0.982,1.344) | 1.314 (1.036,1.588) | 1.167 (1.033,1.309) | 1.290 (1.040,1.600) |
| 35-39 | 1.148 (0.982,1.344) | 1.305 (1.035,1.569) | 1.167 (1.033,1.309) | 1.290 (1.040,1.600) |
| 40-44 | 1.148 (0.982,1.344) | 1.288 (1.034,1.536) | 1.167 (1.033,1.309) | 1.290 (1.040,1.600) |
| 45-49 | 1.122 (0.985,1.282) | 1.260 (1.031,1.480) | 1.167 (1.033,1.309) | 1.290 (1.040,1.600) |
| 50-54 | 1.122 (0.985,1.282) | 1.236 (1.028,1.433) | 1.167 (1.033,1.309) | 1.290 (1.040,1.600) |
| 55-59 | 1.099 (0.988,1.226) | 1.213 (1.026,1.389) | 1.167 (1.033,1.309) | 1.290 (1.040,1.600) |
| 60-64 | 1.099 (0.988,1.226) | 1.190 (1.023,1.345) | 1.167 (1.033,1.309) | 1.290 (1.040,1.600) |
| 65-69 | 1.080 (0.990,1.180) | 1.169 (1.021,1.306) | 1.167 (1.033,1.309) | 1.290 (1.040,1.600) |
| 70-74 | 1.080 (0.990,1.180) | 1.150 (1.019,1.269) | 1.167 (1.033,1.309) | 1.290 (1.040,1.600) |
| 75-79 | 1.054 (0.993,1.119) | 1.128 (1.016,1.229) | 1.167 (1.033,1.309) | 1.290 (1.040,1.600) |
| 80-84 | 1.054 (0.993,1.119) | 1.086 (1.011,1.152) | 1.167 (1.033,1.309) | 1.290 (1.040,1.600) |
| 85-89 | 1.022 (0.997,1.047) | 1.086 (1.011,1.152) | 1.167 (1.033,1.309) | 1.290 (1.040,1.600) |
| >90 | 1.022 (0.997,1.047) | 1.086 (1.011,1.152) | 1.167 (1.033,1.309) | 1.290 (1.040,1.600) |

**Unit of RR:** per 100g/day. The recommended consumption level is ≤ 45g/day. In analysis, the relative risks of consumption between 46-50 g/day were approximated to the relative risks of consumption 50-99 g/day.

**Source of RR:** (1) type 2 diabetes and colorectal cancer: Afshin, A., Sur, P. J., Fay, K. A., et al. (2019). Health effects of dietary risks in 195 countries, 1990–2017: a systematic analysis for the Global Burden of Disease Study 2017. The Lancet, 393(10184), 1958-1972. https://doi.org/10.1016/S0140-6736(19)30041-8; (2) stroke: Shi, W., Huang, X., Schooling, C. M., & Zhao, J. V. (2023). Red meat consumption, cardiovascular diseases, and diabetes: a systematic review and meta-analysis. European Heart Journal, 44(28), 2626-2635. https://doi.org/10.1093/eurheartj/ehad336; (3) lung cancer: Farvid, M. S., Sidahmed, E., Spence, N. D., Mante Angua, K., Rosner, B. A., & Barnett, J. B. (2021). Consumption of red meat and processed meat and cancer incidence: a systematic review and meta-analysis of prospective studies. European Journal of Epidemiology, 36, 937-951. https://doi.org/10.1007/s10654-021-00741-9

**(i)** Sugary beverages, relative risks per age groups

| Age groups (years) | Coronary heart disease | Type 2 diabetes |
| --- | --- | --- |
| 25-29 | 1.377 (0.933,1.883) | 1.263 (1.129,1.400) |
| 30-34 | 1.311 (0.943,1.717) | 1.257 (1.126,1.390) |
| 35-39 | 1.232 (0.955,1.521) | 1.250 (1.123,1.379) |
| 40-44 | 1.195 (0.961,1.436) | 1.237 (1.117,1.358) |
| 45-49 | 1.186 (0.963,1.413) | 1.214 (1.106,1.322) |
| 50-54 | 1.172 (0.965,1.381) | 1.195 (1.097,1.292) |
| 55-59 | 1.156 (0.968,1.343) | 1.177 (1.088,1.264) |
| 60-64 | 1.140 (0.971,1.306) | 1.158 (1.079,1.235) |
| 65-69 | 1.124 (0.974,1.270) | 1.141 (1.071,1.209) |
| 70-74 | 1.110 (0.977,1.238) | 1.125 (1.063,1.185) |
| 75-79 | 1.095 (0.980,1.205) | 1.107 (1.055,1.158) |
| 80-84 | 1.067 (0.985,1.143) | 1.080 (1.041,1.118) |
| 85-89 | 1.067 (0.985,1.143) | 1.080 (1.041,1.118) |
| >90 | 1.067 (0.985,1.143) | 1.080 (1.041,1.118) |

**Unit of RR:** per 226.8g/day. The recommended consumption level is 0 g/day. In analysis, consumption below 100 g/day was considered minimal tolerable optimal consumption with no excessive risks.

**Source of RR:** Afshin, A., Sur, P. J., Fay, K. A., et al. (2019). Health effects of dietary risks in 195 countries, 1990–2017: a systematic analysis for the Global Burden of Disease Study 2017. The Lancet, 393(10184), 1958-1972. https://doi.org/10.1016/S0140-6736(19)30041-8

**(j)** Fish, relative risks per age groups

| Age groups (years) | Coronary heart disease | Stroke |
| --- | --- | --- |
| 25-29 | 1.055 (1.041,1.070) | 1.038 (1.008,1.065) |
| 30-34 | 1.055 (1.041,1.070) | 1.038 (1.008,1.065) |
| 35-39 | 1.055 (1.041,1.070) | 1.038 (1.008,1.065) |
| 40-44 | 1.055 (1.041,1.070) | 1.038 (1.008,1.065) |
| 45-49 | 1.047 (1.035,1.060) | 1.032 (1.007,1.054) |
| 50-54 | 1.047 (1.035,1.060) | 1.032 (1.007,1.054) |
| 55-59 | 1.039 (1.029,1.050) | 1.025 (1.006,1.043) |
| 60-64 | 1.039 (1.029,1.050) | 1.025 (1.006,1.043) |
| 65-69 | 1.031 (1.023,1.039) | 1.019 (1.004,1.032) |
| 70-74 | 1.031 (1.023,1.039) | 1.019 (1.004,1.032) |
| 75-79 | 1.024 (1.018,1.031) | 1.012 (1.003,1.021) |
| 80-84 | 1.024 (1.018,1.031) | 1.012 (1.003,1.021) |
| 85-89 | 1.014 (1.010,1.017) | 1.004 (1.001,1.006) |
| >90 | 1.014 (1.010,1.017) | 1.004 (1.001,1.006) |

**Unit of RR:** per 15g/day

**Source of RR:** (1) coronary heart disease: Zhang, B., Xiong, K., Cai, J., & Ma, A. (2020). Fish consumption and coronary heart disease: a meta-analysis. Nutrients, 12(8), 2278. https://doi.org/10.3390/nu12082278; (2) stroke: Zhao, W., Tang, H., Yang, X., et al. (2019). Fish consumption and stroke risk: a meta-analysis of prospective cohort studies. Journal of Stroke and Cerebrovascular Diseases, 28(3), 604-611. https://doi.org/10.1016/j.jstrokecerebrovasdis.2018.10.036

**(k)** Tea, relative risks per age groups

| Age groups (years) | Type 2 diabetes | Stroke |
| --- | --- | --- |
| 25-29 | 1.071 (1.027,1.118) | 1.134 (1.031,1.251) |
| 30-34 | 1.071 (1.027,1.118) | 1.134 (1.031,1.251) |
| 35-39 | 1.071 (1.027,1.118) | 1.134 (1.031,1.251) |
| 40-44 | 1.071 (1.027,1.118) | 1.134 (1.031,1.251) |
| 45-49 | 1.061 (1.024,1.102) | 1.096 (1.023,1.177) |
| 50-54 | 1.061 (1.024,1.102) | 1.096 (1.023,1.177) |
| 55-59 | 1.053 (1.020,1.087) | 1.064 (1.015,1.116) |
| 60-64 | 1.053 (1.020,1.087) | 1.064 (1.015,1.116) |
| 65-69 | 1.044 (1.017,1.073) | 1.042 (1.010,1.075) |
| 70-74 | 1.044 (1.017,1.073) | 1.042 (1.010,1.075) |
| 75-79 | 1.033 (1.013,1.054) | 1.025 (1.006,1.045) |
| 80-84 | 1.033 (1.013,1.054) | 1.025 (1.006,1.045) |
| 85-89 | 1.020 (1.008,1.032) | 0.997 (0.996,0.999) |
| >90 | 1.020 (1.008,1.032) | 0.997 (0.996,0.999) |

**Unit of RR:** per 200g/day. In analysis, RR = 1 was used for stroke above age 85 years.

**Source of RR:** (1) type 2 diabetes: Yang, W. S., Wang, W. Y., Fan, W. Y., Deng, Q., & Wang, X. (2014). Tea consumption and risk of type 2 diabetes: a dose–response meta-analysis of cohort studies. British Journal of Nutrition, 111(8), 1329-1339. https://doi.org/10.1017/S0007114513003887; (2) stroke: Chung, M., Zhao, N., Wang, D., et al. (2020). Dose–response relation between tea consumption and risk of cardiovascular disease and all-cause mortality: a systematic review and meta-analysis of population-based studies. Advances in Nutrition, 11(4), 790-814. https://doi.org/10.1093/advances/nmaa010

**Supplementary Table S3.** Projected number and percentage of preventable NCD cases per 100,000 women or men (and 95% Uncertainty Intervals [UIs]) in 2030 (a) and 2040 (b) if the entire population adhered to Dutch dietary guidelines

**(a)** Year 2030

|  | **Women** | | **Men** | |
| --- | --- | --- | --- | --- |
| **Food groups / NCDs** | **Cases preventable** | **Percentage, %** | **Cases preventable** | **Percentage, %** |
| **Fruit** |  |  |  |  |
| Coronary heart disease | 196 (34, 338) | 5.0 (0.9, 8.7) | 265 (49, 452) | 4.3 (0.8, 7.3) |
| Stroke | 188 (111, 238) | 10.8 (6.4, 13.6) | 230 (140, 287) | 12.1 (7.4, 15.1) |
| Type 2 diabetes | 207 (27, 355) | 3.2 (0.4, 5.5) | 259 (31, 448) | 3.6 (0.4, 6.2) |
| Colorectal cancer | -2 (-2, -1) | -0.2 (-0.3, -0.1) | -5 (-6, -3) | -0.5 (-0.7, -0.3) |
| Lung cancer | 15 (8, 23) | 8.1 (4.5, 12.5) | 21 (11, 33) | 8.0 (4.3, 12.7) |
| **Vegetables** |  |  |  |  |
| Coronary heart disease | 170 (11, 271) | 4.4 (0.3, 7.0) | 229 (27, 357) | 3.7 (0.4, 5.8) |
| Stroke | 54 (19, 88) | 3.1 (1.1, 5.0) | 65 (26, 101) | 3.4 (1.4, 5.3) |
| Type 2 diabetes | -7 (-11, -1) | -0.1 (-0.2, 0) | -14 (-22, -1) | -0.2 (-0.3, 0) |
| Colorectal cancer | 10 (2, 17) | 1.3 (0.3, 2.4) | 11 (2, 21) | 1.3 (0.2, 2.4) |
| Lung cancer | 0 (0, 0) | 0 (-0.1, 0) | 0 (-1, 0) | -0.2 (-0.3, 0) |
| **Whole grains** |  |  |  |  |
| Coronary heart disease | 210 (114, 283) | 5.4 (2.9, 7.3) | 225 (126, 300) | 3.6 (2.0, 4.9) |
| Stroke | 114 (91, 138) | 6.6 (5.2, 7.9) | 113 (90, 134) | 5.9 (4.8, 7.0) |
| Type 2 diabetes | 243 (114, 338) | 3.8 (1.8, 5.2) | 242 (113, 338) | 3.3 (1.6, 4.7) |
| Colorectal cancer | 38 (30, 52) | 5.3 (4.2, 7.2) | 38 (30, 52) | 4.3 (3.3, 5.9) |
| Lung cancer | 0 (0, 0) | -0.1 (-0.1, -0.1) | -1 (-1, 0) | -0.2 (-0.3, -0.2) |
| **Nuts/seeds** |  |  |  |  |
| Coronary heart disease | 347 (65, 593) | 8.9 (1.7, 15.3) | 426 (96, 719) | 6.9 (1.6, 11.7) |
| Stroke | -1 (-3, 2) | 0 (-0.2, 0.1) | -4 (-10, 2) | -0.2 (-0.5, 0.1) |
| Type 2 diabetes | 236 (126, 333) | 3.7 (2.0, 5.2) | 262 (133, 371) | 3.6 (1.8, 5.1) |
| Colorectal cancer | -1 (-2, 0) | -0.1 (-0.3, 0) | -3 (-6, 0) | -0.4 (-0.7, 0) |
| Lung cancer | 0 (0, 0) | -0.1 (-0.1, 0) | -1 (-1, 0) | -0.3 (-0.5, 0) |
| **Legumes** |  |  |  |  |
| Coronary heart disease | 48 (16, 79) | 1.2 (0.4, 2.0) | 65 (24, 105) | 1.1 (0.4, 1.7) |
| Stroke | 0 (-1, 0) | 0 (0, 0) | -1 (-1, 0) | 0 (-0.1, 0) |
| Type 2 diabetes | -1 (-2, 0) | 0 (0, 0) | -3 (-6, 0) | 0 (-0.1, 0) |
| Colorectal cancer | 0 (0, 0) | 0 (0, 0) | 0 (-1, 0) | 0 (-0.1, 0) |
| Lung cancer | 0 (0, 0) | 0 (0, 0) | 0 (0, 0) | 0 (-0.1, 0) |
| **Fish** |  |  |  |  |
| Coronary heart disease | 51 (36, 68) | 1.3 (0.9, 1.7) | 67 (49, 88) | 1.1 (0.8, 1.4) |
| Stroke | 14 (4, 25) | 0.8 (0.2, 1.4) | 16 (5, 28) | 0.8 (0.3, 1.5) |
| Type 2 diabetes | -2 (-3, -1) | 0 (0, 0) | -4 (-6, -3) | -0.1 (-0.1, 0) |
| Colorectal cancer | 0 (0, 0) | 0 (0, 0) | -1 (-1, 0) | -0.1 (-0.1, 0) |
| Lung cancer | 0 (0, 0) | 0 (0, 0) | 0 (0, 0) | -0.1 (-0.1, 0) |
| **Red meat** |  |  |  |  |
| Coronary heart disease | -1 (-3, 0) | 0 (-0.1, 0) | -6 (-11, -1) | -0.1 (-0.2, 0) |
| Stroke | 13 (-3, 27) | 0.7 (-0.2, 1.6) | 20 (-5, 43) | 1.1 (-0.3, 2.3) |
| Type 2 diabetes | 67 (15, 120) | 1.0 (0.2, 1.9) | 121 (27, 214) | 1.7 (0.4, 3.0) |
| Colorectal cancer | 13 (5, 22) | 1.8 (0.7, 3.1) | 23 (8, 40) | 2.6 (0.9, 4.5) |
| Lung cancer | 8 (2, 14) | 4.2 (0.9, 7.7) | 16 (3, 29) | 6.0 (1.3, 11.0) |
| **Processed meat** |  |  |  |  |
| Coronary heart disease | 430 (68, 855) | 11.1 (1.7, 22.0) | 740 (144, 1374) | 12.0 (2.3, 22.3) |
| Stroke | 94 (26, 153) | 5.4 (1.5, 8.8) | 143 (46, 229) | 7.5 (2.4, 12.0) |
| Type 2 diabetes | 604 (369, 892) | 9.4 (5.7, 13.8) | 961 (595, 1392) | 13.3 (8.2, 19.3) |
| Colorectal cancer | 48 (30, 71) | 6.7 (4.2, 9.8) | 81 (48, 121) | 9.2 (5.4, 13.7) |
| Lung cancer | 0 (0, 0) | -0.1 (-0.2, -0.1) | -2 (-3, -1) | -0.7 (-1.2, -0.3) |
| **Dairy products** |  |  |  |  |
| Coronary heart disease | 0 (-1, 0) | 0 (0, 0) | -1 (-1, -1) | 0 (0, 0) |
| Stroke | 0 (0, 0) | 0 (0, 0) | 0 (0, 0) | 0 (0, 0) |
| Type 2 diabetes | -1 (-1, 0) | 0 (0, 0) | -1 (-1, -1) | 0 (0, 0) |
| Colorectal cancer | 19 (12, 25) | 2.6 (1.7, 3.5) | 23 (15, 31) | 2.5 (1.6, 3.4) |
| Lung cancer | 0 (0, 0) | 0 (0, 0) | 0 (0, 0) | 0 (0, 0) |
| **Tea** |  |  |  |  |
| Coronary heart disease | 1 (-1, 4) | 0 (0, 0.1) | 0 (-4, 5) | 0 (-0.1, 0.1) |
| Stroke | 92 (36, 140) | 5.3 (2.1, 8.0) | 125 (54, 184) | 6.6 (2.9, 9.7) |
| Type 2 diabetes | 182 (76, 296) | 2.8 (1.2, 4.6) | 253 (116, 405) | 3.5 (1.6, 5.6) |
| Colorectal cancer | 0 (-1, 0) | -0.1 (-0.1, 0) | -1 (-2, 0) | -0.1 (-0.2, 0) |
| Lung cancer | 0 (0, 0) | 0 (-0.1, 0) | 0 (0, 0) | -0.1 (-0.2, 0) |
| **Sugary beverages** |  |  |  |  |
| Coronary heart disease | 101 (-16, 208) | 2.6 (-0.4, 5.4) | 193 (-17, 377) | 3.1 (-0.3, 6.1) |
| Stroke | 0 (-1, 1) | 0 (0, 0.1) | 0 (-3, 3) | 0 (-0.1, 0.2) |
| Type 2 diabetes | 153 (94, 207) | 2.4 (1.5, 3.2) | 268 (166, 359) | 3.7 (2.3, 5.0) |
| Colorectal cancer | 0 (-1, 0) | 0 (-0.1, 0) | -1 (-2, 0) | -0.1 (-0.2, 0.1) |
| Lung cancer | 0 (0, 0) | 0 (0, 0) | 0 (-1, 0) | -0.1 (-0.2, 0) |

**(b)** Year 2040

|  | **Women** | | **Men** | |
| --- | --- | --- | --- | --- |
| **Food groups / NCDs** | **Cases preventable** | **Percentage, %** | **Cases preventable** | **Percentage, %** |
| **Fruit** |  |  |  |  |
| Coronary heart disease | 328 (63, 563) | 7.3 (1.4, 12.6) | 435 (85, 741) | 6.9 (1.4, 11.8) |
| Stroke | 314 (192, 393) | 15.3 (9.3, 19.1) | 371 (231, 458) | 17.1 (10.7, 21.2) |
| Type 2 diabetes | 354 (48, 607) | 5.2 (0.7, 8.9) | 420 (45, 733) | 5.4 (0.6, 9.3) |
| Colorectal cancer | -4 (-5, -3) | -0.5 (-0.7, -0.3) | -11 (-15, -7) | -1.2 (-1.5, -0.7) |
| Lung cancer | 15 (8, 23) | 8.1 (4.4, 12.6) | 22 (11, 35) | 7.8 (3.9, 12.6) |
| **Vegetables** |  |  |  |  |
| Coronary heart disease | 283 (24, 449) | 6.3 (0.5, 10.0) | 376 (48, 587) | 6.0 (0.8, 9.3) |
| Stroke | 91 (35, 143) | 4.4 (1.7, 7.0) | 104 (45, 160) | 4.8 (2.1, 7.4) |
| Type 2 diabetes | -15 (-22, -3) | -0.2 (-0.3, 0) | -31 (-48, -5) | -0.4 (-0.6, -0.1) |
| Colorectal cancer | 13 (2, 24) | 1.7 (0.3, 3.1) | 14 (1, 29) | 1.5 (0.1, 3.0) |
| Lung cancer | 0 (0, 0) | -0.1 (-0.1, 0) | -1 (-1, 0) | -0.3 (-0.5, -0.1) |
| **Whole grains** |  |  |  |  |
| Coronary heart disease | 343 (190, 458) | 7.7 (4.3, 10.3) | 368 (209, 490) | 5.9 (3.3, 7.8) |
| Stroke | 187 (150, 223) | 9.1 (7.3, 10.9) | 182 (147, 214) | 8.4 (6.8, 9.9) |
| Type 2 diabetes | 404 (192, 561) | 5.9 (2.8, 8.2) | 392 (183, 548) | 5.0 (2.3, 7.0) |
| Colorectal cancer | 51 (40, 70) | 6.6 (5.1, 9.0) | 49 (37, 69) | 5.1 (3.9, 7.1) |
| Lung cancer | 0 (0, 0) | -0.2 (-0.2, -0.1) | -1 (-2, -1) | -0.5 (-0.6, -0.4) |
| **Nuts/seeds** |  |  |  |  |
| Coronary heart disease | 583 (123, 986) | 13.0 (2.8, 22.0) | 700 (174, 1171) | 11.1 (2.8, 18.6) |
| Stroke | 0 (-6, 5) | 0 (-0.3, 0.2) | -7 (-19, 6) | -0.3 (-0.9, 0.3) |
| Type 2 diabetes | 404 (218, 569) | 5.9 (3.2, 8.3) | 426 (215, 601) | 5.4 (2.7, 7.7) |
| Colorectal cancer | -2 (-4, 0) | -0.3 (-0.5, 0) | -8 (-13, -1) | -0.8 (-1.4, -0.1) |
| Lung cancer | 0 (0, 0) | -0.1 (-0.2, 0) | -2 (-3, 0) | -0.6 (-1.0, -0.1) |
| **Legumes** |  |  |  |  |
| Coronary heart disease | 80 (28, 130) | 1.8 (0.6, 2.9) | 108 (43, 172) | 1.7 (0.7, 2.7) |
| Stroke | -1 (-1, 0) | 0 (-0.1, 0) | -2 (-3, 0) | -0.1 (-0.1, 0) |
| Type 2 diabetes | -2 (-4, 0) | 0 (-0.1, 0) | -6 (-11, -1) | -0.1 (-0.1, 0) |
| Colorectal cancer | 0 (0, 0) | 0 (-0.1, 0) | -1 (-2, 0) | -0.1 (-0.2, 0) |
| Lung cancer | 0 (0, 0) | 0 (0, 0) | 0 (0, 0) | -0.1 (-0.1, 0) |
| **Fish** |  |  |  |  |
| Coronary heart disease | 84 (60, 111) | 1.9 (1.4, 2.5) | 110 (80, 143) | 1.7 (1.3, 2.3) |
| Stroke | 23 (7, 40) | 1.1 (0.3, 1.9) | 25 (8, 43) | 1.1 (0.4, 2.0) |
| Type 2 diabetes | -4 (-6, -2) | -0.1 (-0.1, 0) | -9 (-13, -6) | -0.1 (-0.2, -0.1) |
| Colorectal cancer | 0 (-1, 0) | -0.1 (-0.1, 0) | -1 (-2, -1) | -0.1 (-0.2, -0.1) |
| Lung cancer | 0 (0, 0) | 0 (0, 0) | 0 (0, 0) | -0.1 (-0.1, -0.1) |
| **Red meat** |  |  |  |  |
| Coronary heart disease | -2 (-6, 2) | 0 (-0.1, 0) | -10 (-21, 0) | -0.2 (-0.3, 0) |
| Stroke | 20 (-5, 43) | 1.0 (-0.2, 2.1) | 31 (-8, 66) | 1.4 (-0.3, 3.0) |
| Type 2 diabetes | 114 (24, 203) | 1.7 (0.4, 3.0) | 197 (45, 347) | 2.5 (0.6, 4.4) |
| Colorectal cancer | 18 (6, 31) | 2.3 (0.8, 4.0) | 30 (10, 53) | 3.1 (1.1, 5.5) |
| Lung cancer | 8 (2, 15) | 4.3 (0.9, 8.1) | 16 (3, 31) | 5.9 (1.2, 11.0) |
| **Processed meat** |  |  |  |  |
| Coronary heart disease | 727 (134, 1436) | 16.3 (3.0, 32.1) | 1216 (265, 2263) | 19.4 (4.2, 36.1) |
| Stroke | 157 (51, 250) | 7.6 (2.5, 12.2) | 223 (79, 356) | 10.3 (3.7, 16.5) |
| Type 2 diabetes | 1044 (642, 1542) | 15.3 (9.4, 22.6) | 1580 (977, 2293) | 20.2 (12.5, 29.4) |
| Colorectal cancer | 67 (41, 100) | 8.7 (5.3, 12.8) | 104 (56, 160) | 10.7 (5.8, 16.5) |
| Lung cancer | -1 (-1, 0) | -0.3 (-0.4, -0.2) | -4 (-7, -2) | -1.5 (-2.4, -0.7) |
| **Dairy products** |  |  |  |  |
| Coronary heart disease | -1 (-2, -1) | 0 (0, 0) | -3 (-4, -2) | 0 (-0.1, 0) |
| Stroke | -1 (-1, 0) | 0 (0, 0) | -1 (-1, -1) | 0 (-0.1, 0) |
| Type 2 diabetes | -2 (-2, -1) | 0 (0, 0) | -3 (-4, -2) | 0 (0, 0) |
| Colorectal cancer | 26 (17, 35) | 3.4 (2.2, 4.6) | 31 (20, 43) | 3.2 (2.1, 4.4) |
| Lung cancer | 0 (0, 0) | 0 (0, 0) | 0 (0, 0) | 0 (-0.1, 0) |
| **Tea** |  |  |  |  |
| Coronary heart disease | 4 (-2, 11) | 0.1 (0, 0.2) | 3 (-8, 15) | 0 (-0.1, 0.2) |
| Stroke | 155 (67, 230) | 7.5 (3.2, 11.2) | 203 (96, 293) | 9.4 (4.4, 13.5) |
| Type 2 diabetes | 310 (133, 502) | 4.5 (1.9, 7.3) | 416 (195, 666) | 5.3 (2.5, 8.5) |
| Colorectal cancer | -1 (-2, 0) | -0.2 (-0.2, -0.1) | -3 (-5, -1) | -0.3 (-0.5, -0.1) |
| Lung cancer | 0 (0, 0) | -0.1 (-0.1, 0) | -1 (-1, 0) | -0.2 (-0.4, -0.1) |
| **Sugary beverages** |  |  |  |  |
| Coronary heart disease | 175 (-20, 353) | 3.9 (-0.4, 7.9) | 327 (-14, 627) | 5.2 (-0.2, 10.0) |
| Stroke | 2 (-1, 4) | 0.1 (0, 0.2) | 1 (-5, 8) | 0.1 (-0.2, 0.3) |
| Type 2 diabetes | 269 (166, 362) | 3.9 (2.4, 5.3) | 454 (285, 608) | 5.8 (3.6, 7.7) |
| Colorectal cancer | -1 (-1, 0) | -0.1 (-0.2, 0) | -3 (-5, 0) | -0.3 (-0.5, 0.1) |
| Lung cancer | 0 (0, 0) | 0 (-0.1, 0) | -1 (-1, 0) | -0.2 (-0.4, 0) |

**Supplementary Table S4.** Projected number of total preventable NCD cases (and 95% Uncertainty Intervals [UIs]) by 2050 if the entire population adhered to Dutch dietary guidelines

|  | **Women** | | | **Men** | | |
| --- | --- | --- | --- | --- | --- | --- |
| **Food groups / NCDs** | **By 2030** | **By 2040** | **By 2050** | **By 2030** | **By 2040** | **By 2050** |
| **Fruit** |  |  |  |  |  |  |
| Coronary heart disease | 16950 (2710, 29479) | 27990 (4782, 48551) | 32883 (6193, 56561) | 22262 (3585, 38485) | 36070 (5891, 62484) | 41177 (7386, 70912) |
| Stroke | 16448 (9643, 20813) | 27295 (16522, 34169) | 32204 (20046, 39931) | 19927 (12004, 24926) | 31845 (19579, 39506) | 35794 (22410, 44117) |
| Type 2 diabetes | 17680 (1747, 30827) | 29827 (2856, 52188) | 36140 (3988, 62839) | 21627 (1571, 38271) | 34293 (1462, 61757) | 39375 (1760, 71017) |
| Colorectal cancer | -220 (-286, -129) | -519 (-652, -336) | -748 (-934, -505) | -562 (-753, -338) | -1322 (-1734, -839) | -1886 (-2460, -1207) |
| Lung cancer | 1269 (694, 1983) | 1272 (678, 2010) | 1220 (638, 1941) | 1794 (929, 2857) | 1816 (853, 2992) | 1606 (673, 2718) |
| **Vegetables** |  |  |  |  |  |  |
| Coronary heart disease | 14910 (976, 23803) | 24612 (1988, 39154) | 28522 (2834, 45084) | 19689 (2277, 30795) | 32168 (3991, 50350) | 36328 (5073, 56646) |
| Stroke | 4756 (1652, 7672) | 7824 (2966, 12413) | 9099 (3646, 14240) | 5558 (2240, 8703) | 8820 (3751, 13696) | 9739 (4299, 15031) |
| Type 2 diabetes | -836 (-1253, -163) | -1747 (-2535, -440) | -2295 (-3268, -675) | -1699 (-2643, -209) | -3729 (-5668, -667) | -5013 (-7517, -1065) |
| Colorectal cancer | 833 (160, 1493) | 1114 (167, 2078) | 1102 (108, 2134) | 946 (93, 1842) | 1138 ( -66, 2401) | 959 (-299, 2266) |
| Lung cancer | -13 (-18, -4) | -27 (-36, -10) | -34 (-46, -14) | -54 (-84, -7) | -120 (-179, -25) | -165 (-242, -41) |
| **Whole grains** |  |  |  |  |  |  |
| Coronary heart disease | 18351 (9854, 24737) | 29562 (16211, 39665) | 34538 (19430, 45992) | 19211 (10633, 25700) | 31104 (17420, 41555) | 36353 (20631, 48405) |
| Stroke | 9971 (7884, 12062) | 16178 (12934, 19312) | 19168 (15529, 22620) | 9728 (7749, 11561) | 15550 (12450, 18264) | 18016 (14455, 21012) |
| Type 2 diabetes | 21043 (9668, 29444) | 34652 (16017, 48410) | 41755 (19802, 58011) | 20604 (9266, 29037) | 32920 (14541, 46522) | 38492 (16936, 54437) |
| Colorectal cancer | 3338 (2604, 4529) | 4408 (3411, 6037) | 4450 (3413, 6151) | 3284 (2519, 4530) | 4152 (3105, 5871) | 4102 (2994, 5994) |
| Lung cancer | -25 (-30, -19) | -53 (-62, -42) | -71 (-83, -58) | -77 (-98, -53) | -178 (-220, -133) | -258 (-316, -196) |
| **Nuts/seeds** |  |  |  |  |  |  |
| Coronary heart disease | 30512 (5761, 52153) | 50846 (10742, 86128) | 59218 (13883, 99275) | 36854 (8373, 62152) | 60142 (14976, 100779) | 69742 (18775, 115949) |
| Stroke | -125 (-373, 147) | -221 (-798, 406) | -296 (-1086, 556) | -539 (-1189, 171) | -1065 (-2416, 390) | -1444 (-3213, 444) |
| Type 2 diabetes | 20547 (10802, 29099) | 34916 (18438, 49265) | 41677 (22322, 58517) | 22321 (10807, 31686) | 35821 (17016, 50693) | 41680 (19865, 58760) |
| Colorectal cancer | -119 (-214, -6) | -282 (-474, -47) | -420 (-670, -106) | -384 (-698, -12) | -859 (-1486, -109) | -1221 (-2034, -244) |
| Lung cancer | -17 (-30, -2) | -37 (-59, -9) | -49 (-76, -15) | -94 (-169, -6) | -197 (-336, -31) | -270 (-444, -60) |
| **Legumes** |  |  |  |  |  |  |
| Coronary heart disease | 4201 (1373, 6947) | 6983 (2447, 11390) | 8279 (3169, 13244) | 5674 (2130, 9107) | 9347 (3724, 14803) | 10913 (4665, 16985) |
| Stroke | -31 (-61, -2) | -75 (-143, -6) | -110 (-201, -16) | -87 (-168, -4) | -191 (-361, -18) | -269 (-491, -42) |
| Type 2 diabetes | -127 (-245, -8) | -261 (-492, -26) | -343 (-626, -54) | -342 (-650, -29) | -710 (-1326, -83) | -936 (-1699, -157) |
| Colorectal cancer | -13 (-25, -1) | -28 (-52, -2) | -39 (-72, -6) | -44 (-85, -2) | -93 (-177, -8) | -131 (-241, -20) |
| Lung cancer | -2 (-3, 0) | -4 (-7, -1) | -5 (-8, -1) | -11 (-20, -1) | -22 (-41, -2) | -30 (-53, -5) |
| **Fish** |  |  |  |  |  |  |
| Coronary heart disease | 4471 (3177, 5923) | 7332 (5257, 9672) | 8560 (6185, 11243) | 5797 (4215, 7585) | 9409 (6877, 12286) | 10779 (7928, 14028) |
| Stroke | 1219 (340, 2201) | 1950 (589, 3472) | 2247 (709, 3965) | 1362 (414, 2425) | 2093 (648, 3712) | 2301 (715, 4073) |
| Type 2 diabetes | -236 (-355, -121) | -474 (-696, -265) | -614 (-882, -365) | -510 (-717, -354) | -1073 (-1493, -752) | -1403 (-1931, -999) |
| Colorectal cancer | -23 (-36, -11) | -50 (-75, -27) | -70 (-101, -41) | -64 (-92, -41) | -141 (-200, -95) | -198 (-275, -138) |
| Lung cancer | -4 (-5, -2) | -7 (-10, -4) | -9 (-12, -6) | -16 (-23, -11) | -34 (-47, -24) | -45 (-62, -33) |
| **Red meat** |  |  |  |  |  |  |
| Coronary heart disease | -172 (-358, -35) | -387 (-842, -23) | -489 (-1102, 12) | -812 (-1449, -287) | -1499 (-2761, -476) | -1687 (-3194, -508) |
| Stroke | 1078 (-337, 2363) | 1683 (-524, 3693) | 1889 (-568, 4130) | 1672 (-572, 3670) | 2483 (-884, 5492) | 2732 (-965, 6059) |
| Type 2 diabetes | 5781 (1062, 10435) | 9675 (1738, 17558) | 11589 (2268, 20891) | 10297 (1992, 18473) | 16463 (3118, 29695) | 19578 (4073, 34993) |
| Colorectal cancer | 1111 (408, 1946) | 1522 (539, 2701) | 1550 (531, 2778) | 1957 (697, 3466) | 2522 (834, 4567) | 2518 (772, 4644) |
| Lung cancer | 659 (144, 1228) | 693 (147, 1295) | 656 (135, 1228) | 1351 (289, 2500) | 1415 (276, 2636) | 1380 (249, 2588) |
| **Processed meat** |  |  |  |  |  |  |
| Coronary heart disease | 37647 (5765, 75077) | 63123 (11144, 125310) | 72372 (14445, 142110) | 63625 (12027, 118692) | 103719 (21466, 194787) | 121065 (27298, 227124) |
| Stroke | 8144 (2225, 13287) | 13379 (4305, 21521) | 15111 (5188, 24052) | 12099 (3675, 19756) | 18500 (5852, 30378) | 20405 (6114, 33603) |
| Type 2 diabetes | 52763 (32027, 78292) | 90484 (55129, 134413) | 107735 (66540, 159029) | 82832 (50432, 120898) | 134909 (81692, 197880) | 160931 (98565, 235309) |
| Colorectal cancer | 4201 (2598, 6174) | 5774 (3406, 8618) | 5672 (3166, 8604) | 6926 (3945, 10452) | 8646 (4284, 13477) | 8102 (3357, 12909) |
| Lung cancer | -39 (-61, -20) | -87 (-128, -49) | -115 (-165, -69) | -228 (-384, -84) | -523 (-824, -255) | -727 (-1106, -390) |
| **Dairy products** |  |  |  |  |  |  |
| Coronary heart disease | -61 (-83, -40) | -187 (-252, -121) | -279 (-378, -181) | -134 (-182, -87) | -356 (-482, -231) | -458 (-621, -297) |
| Stroke | -28 (-38, -18) | -90 (-122, -58) | -137 (-186, -89) | -43 (-58, -28) | -126 (-171, -82) | -175 (-237, -114) |
| Type 2 diabetes | -90 (-121, -58) | -250 (-338, -162) | -352 (-477, -229) | -133 (-180, -86) | -375 (-508, -243) | -496 (-671, -321) |
| Colorectal cancer | 1631 (1058, 2207) | 2292 (1487, 3103) | 2404 (1560, 3254) | 1972 (1278, 2670) | 2739 (1775, 3710) | 2731 (1769, 3700) |
| Lung cancer | -2 (-3, -1) | -6 (-8, -4) | -7 (-10, -5) | -6 (-8, -4) | -16 (-22, -10) | -21 (-28, -13) |
| **Tea** |  |  |  |  |  |  |
| Coronary heart disease | -5 (-247, 260) | 135 (-479, 776) | 234 (-579, 1110) | -208 (-668, 382) | -279 (-1255, 913) | -342 (-1566, 1116) |
| Stroke | 8105 (3158, 12299) | 13493 (5803, 20069) | 15860 (7276, 23221) | 10888 (4747, 16041) | 17577 (8313, 25412) | 19968 (10015, 28400) |
| Type 2 diabetes | 15896 (6602, 26007) | 26860 (11390, 43773) | 32115 (13919, 52041) | 21915 (9998, 35341) | 35760 (16573, 57659) | 41436 (19435, 66577) |
| Colorectal cancer | -57 (-96, -13) | -144 (-231, -56) | -222 (-337, -104) | -120 (-199, -35) | -339 (-527, -146) | -539 (-793, -268) |
| Lung cancer | -10 (-16, -3) | -21 (-32, -10) | -29 (-43, -15) | -32 ( -51, -10) | -82 (-124, -38) | -122 (-178, -64) |
| **Sugary beverages** |  |  |  |  |  |  |
| Coronary heart disease | 8865 (-1416, 18311) | 15251 (-1762, 30823) | 18104 (-1352, 35817) | 16698 (-1427, 32633) | 28140 (-1205, 53960) | 33576 (-206, 63366) |
| Stroke | 21 (-85, 118) | 95 (-172, 337) | 155 (-232, 505) | -59 (-383, 254) | -58 (-778, 633) | -78 (-1046, 847) |
| Type 2 diabetes | 13475 (8200, 18203) | 23504 (14439, 31654) | 28695 (17887, 38419) | 23274 (14331, 31414) | 39161 (24282, 52914) | 47367 (29717, 63739) |
| Colorectal cancer | -28 (-62, 11) | -78 (-150, 5) | -131 (-231, -15) | -116 (-260, 48) | -309 (-609, 48) | -500 (-899, -23) |
| Lung cancer | -5 (-10, 1) | -11 (-21,-1) | -17 (-28, -3) | -31 ( -66, 10) | -76 (-147, 7) | -117 (-208, -9) |

**Supplementary Figure S1.** Projected gains in life expectancy and disease-free life expectancy (and 95% Uncertainty Intervals [UIs]) at birth, and at ages 50 and 70 over a 30-year period (by 2050) if the entire population adhered to Dutch dietary guidelines^a^

^a^ Curves denote estimated gains in life expectancy and disease-free life expectancy, and shaded areas denote the uncertainty intervals of the estimates.

**(a1)** Fruit, life expectancy


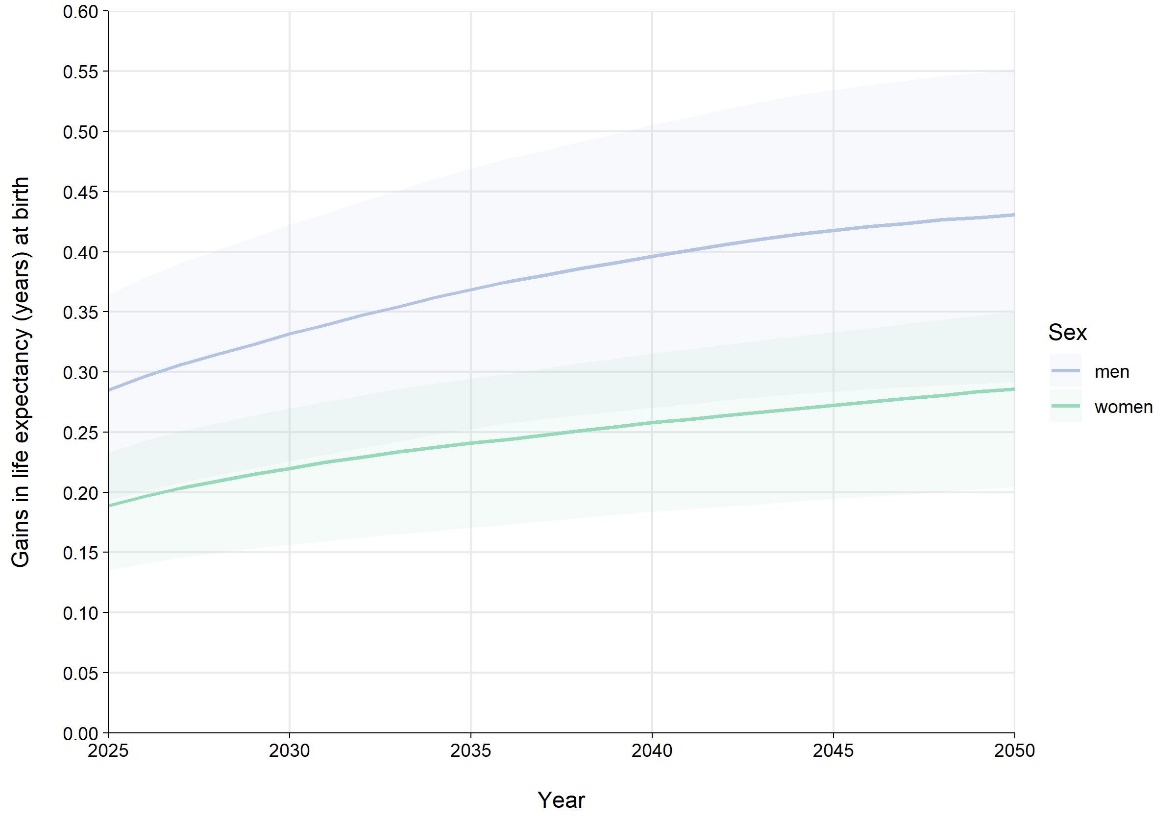


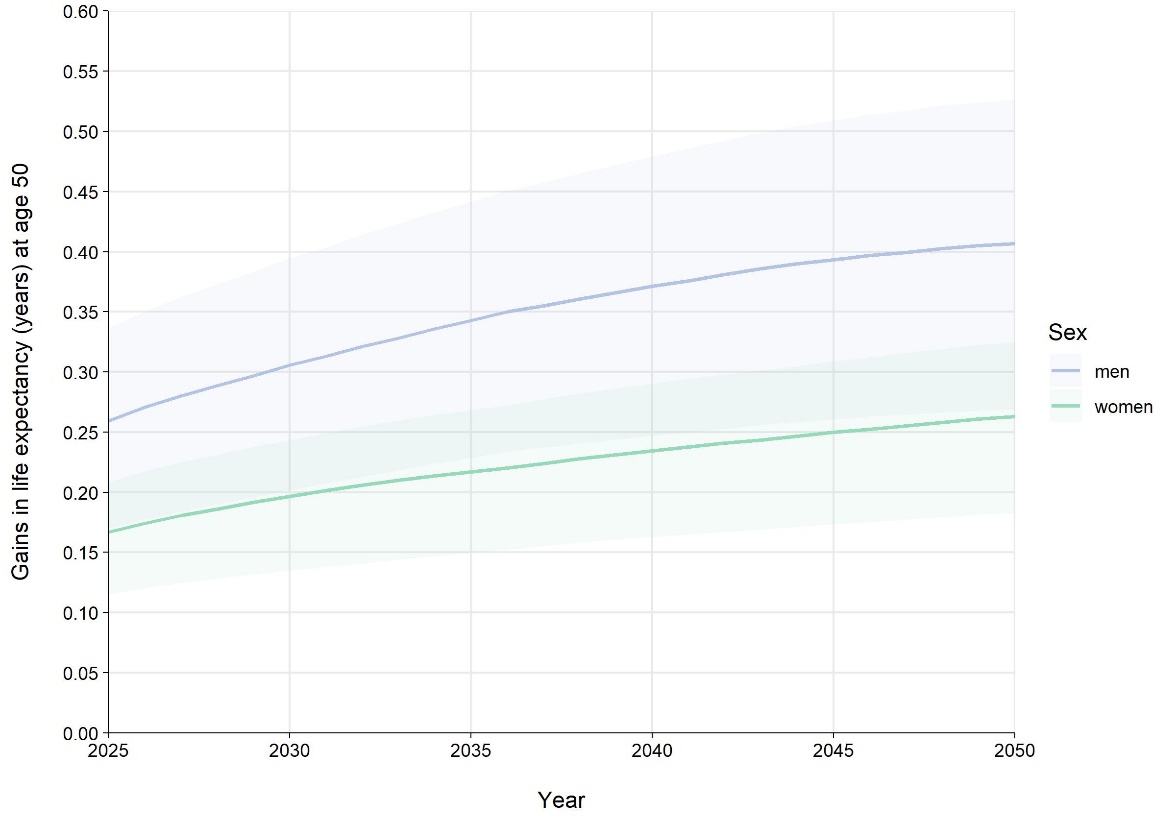


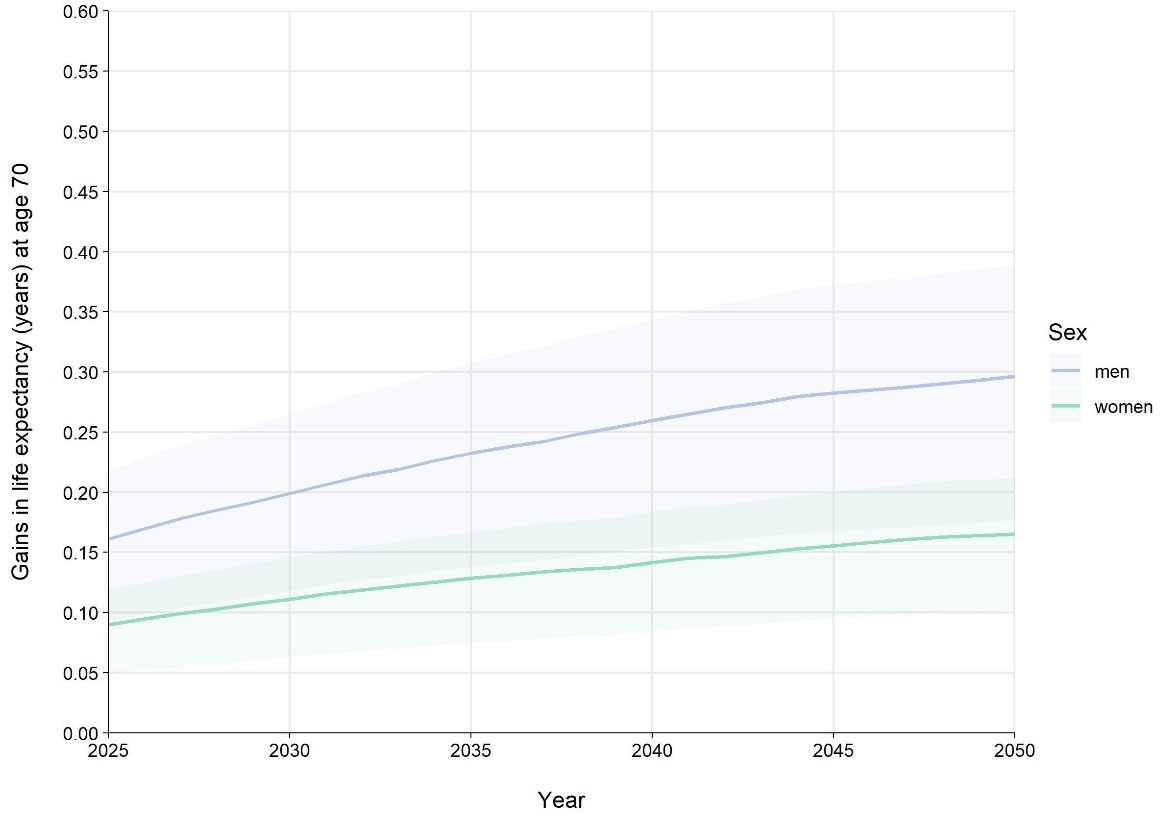


**(a2)** Fruit, disease-free life expectancy


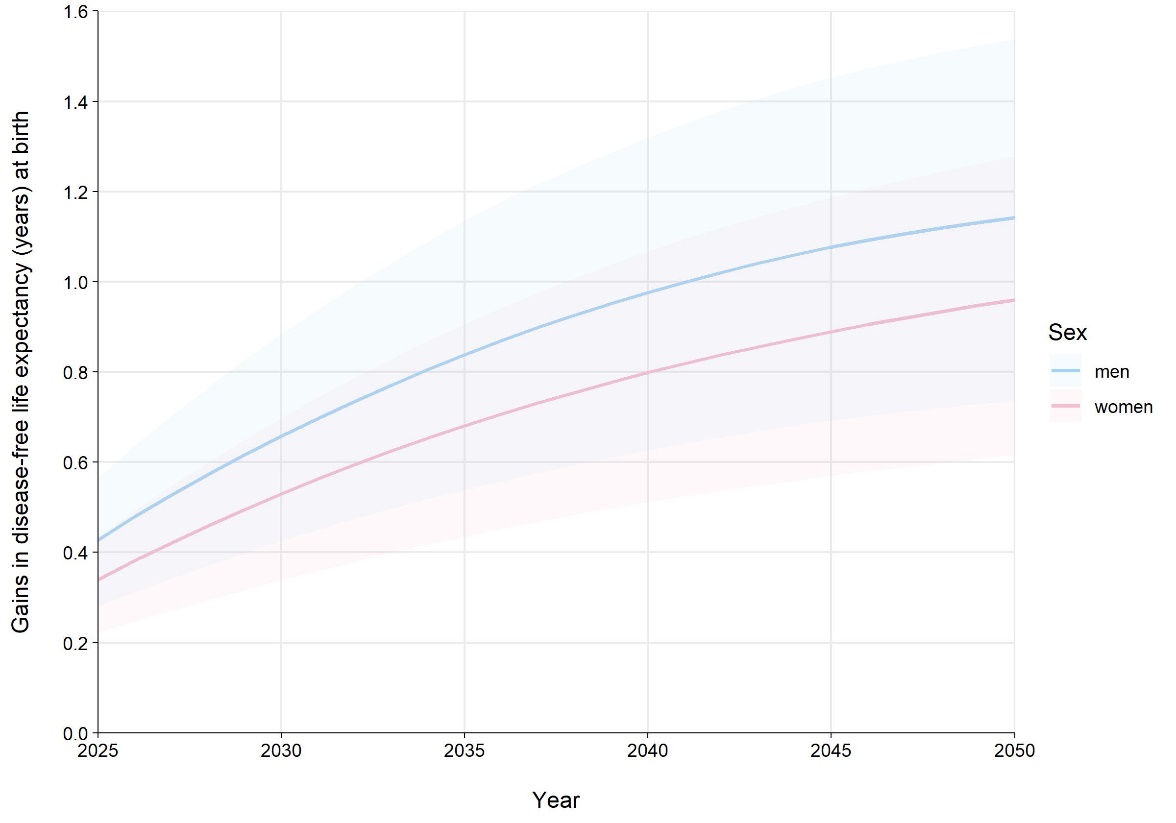


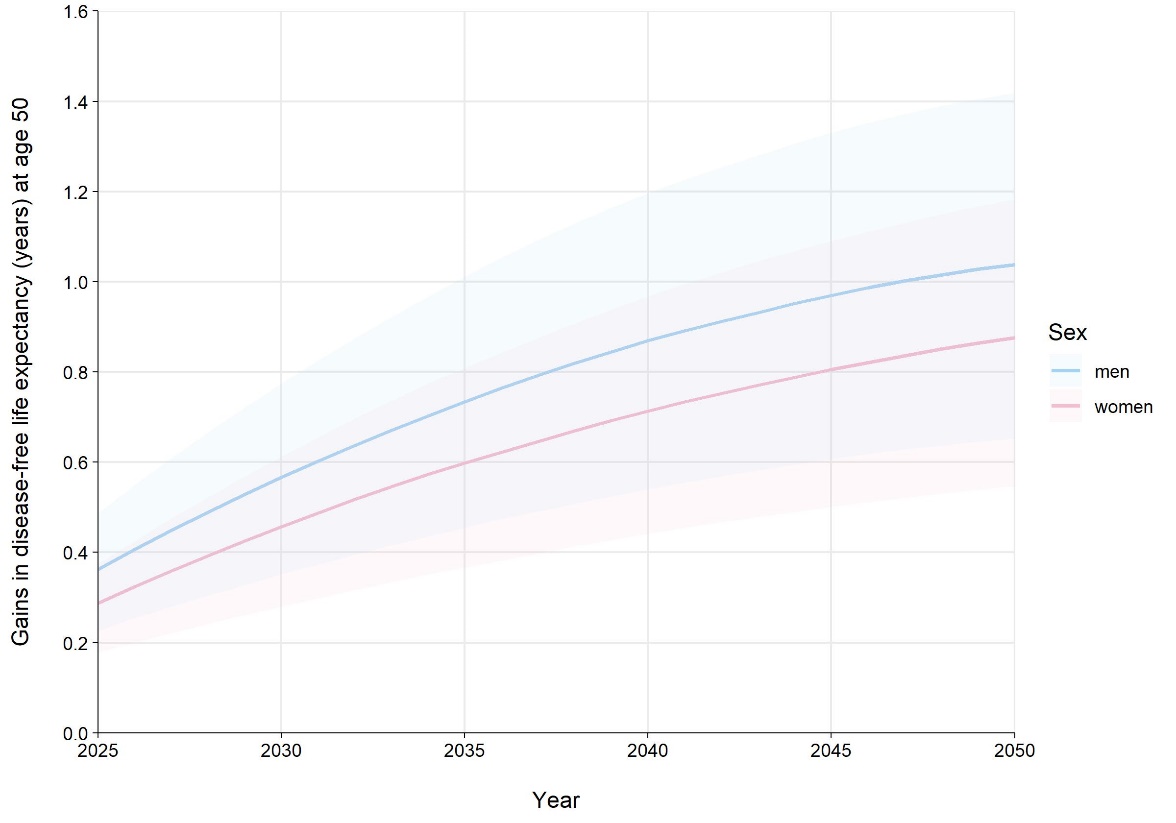


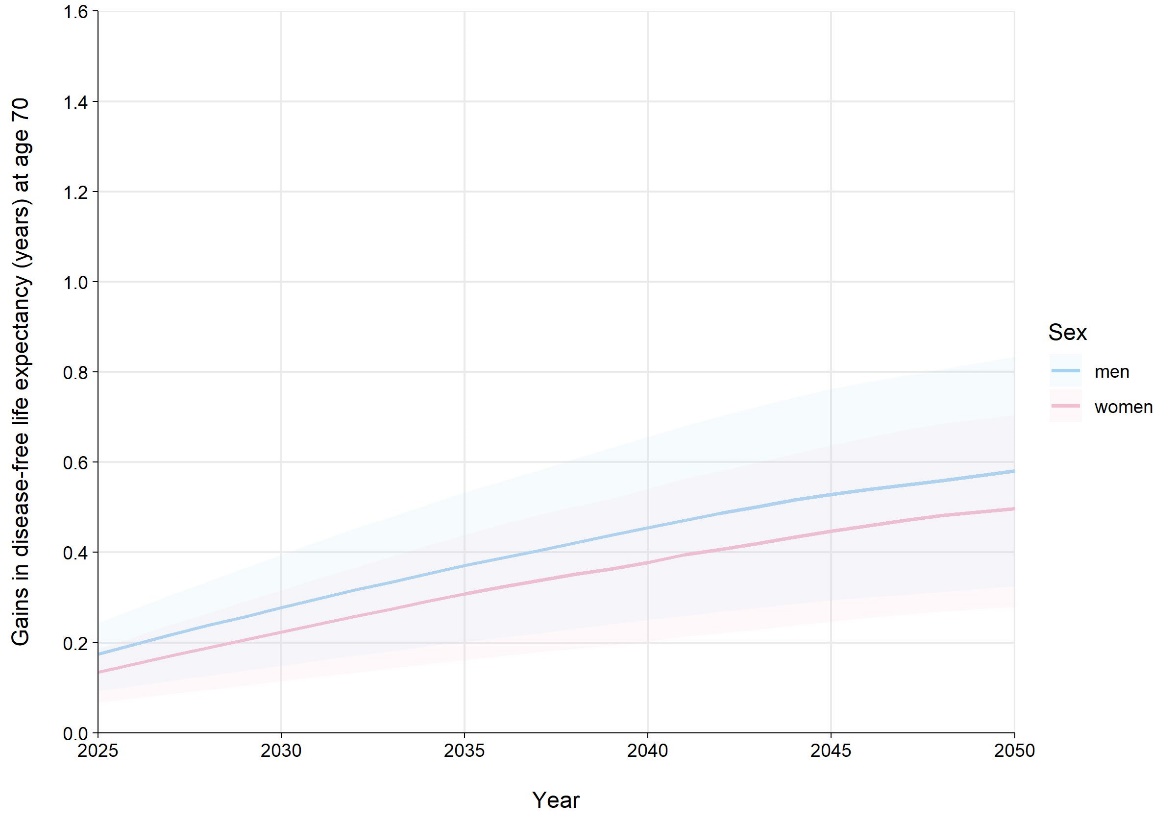


**(b1)** Vegetables, life expectancy


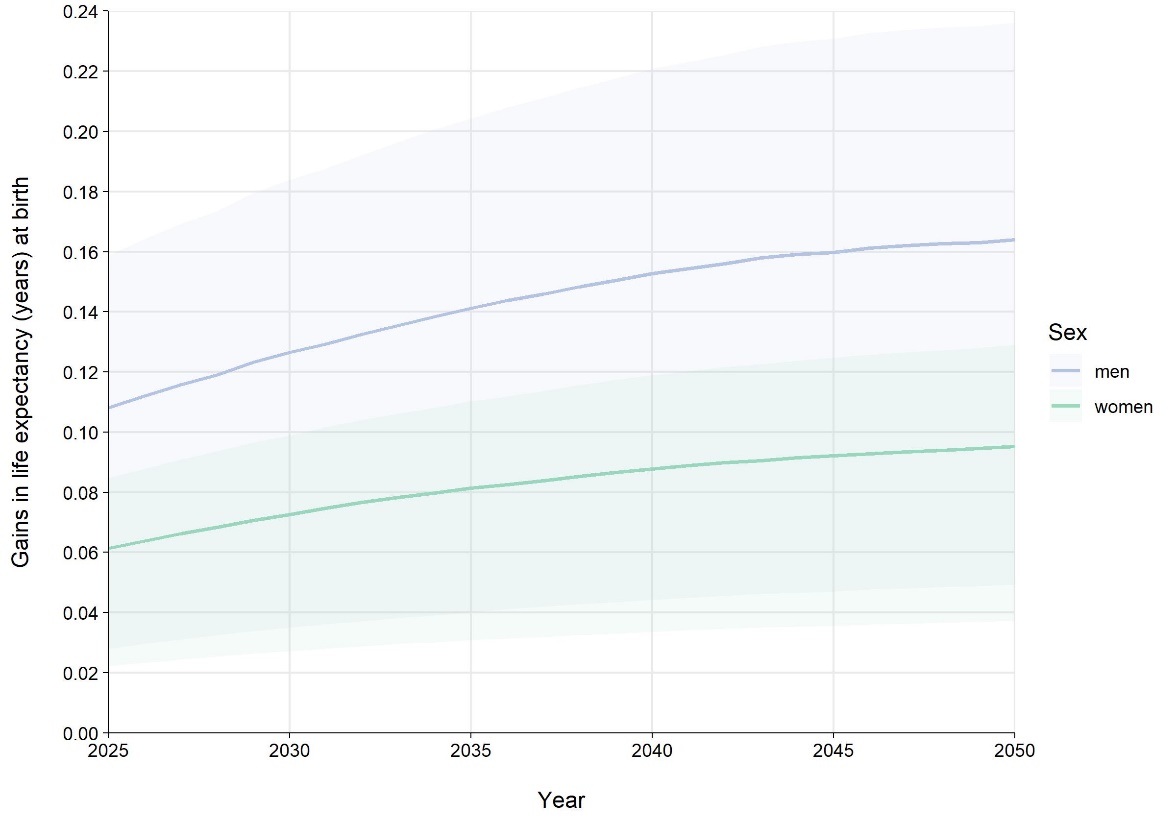


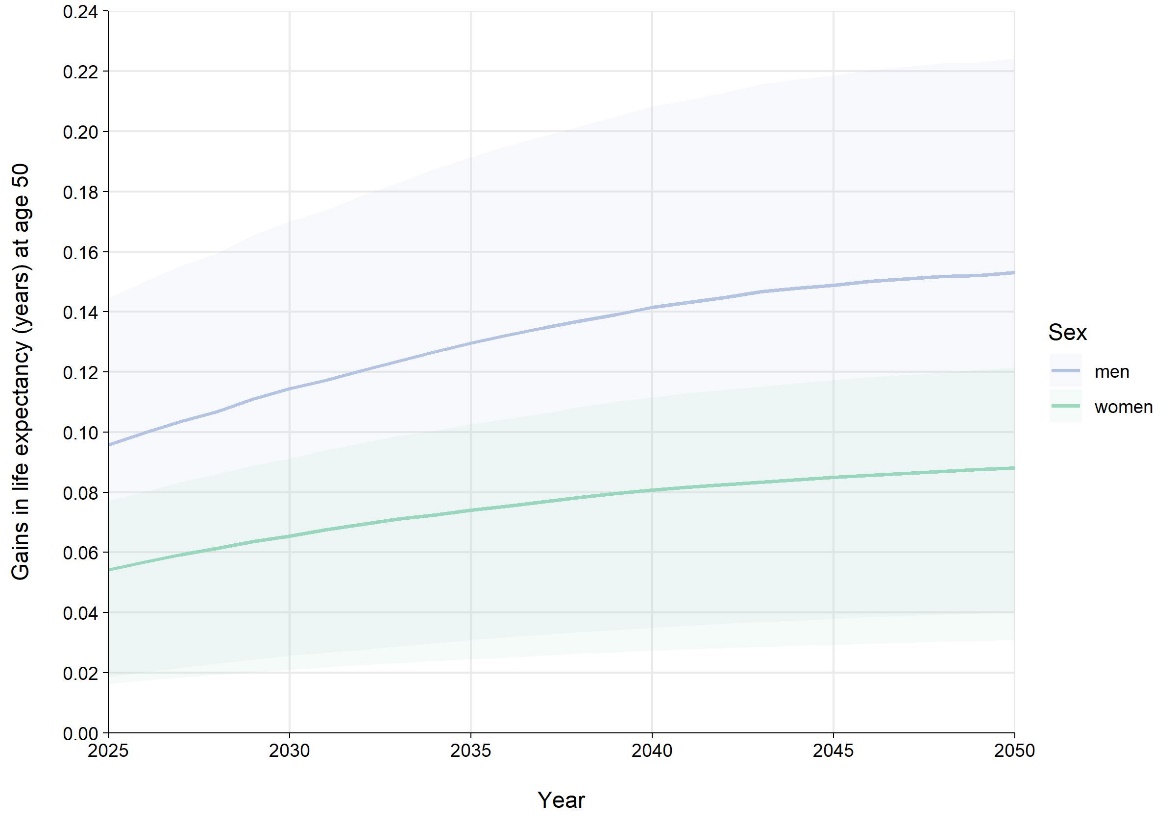


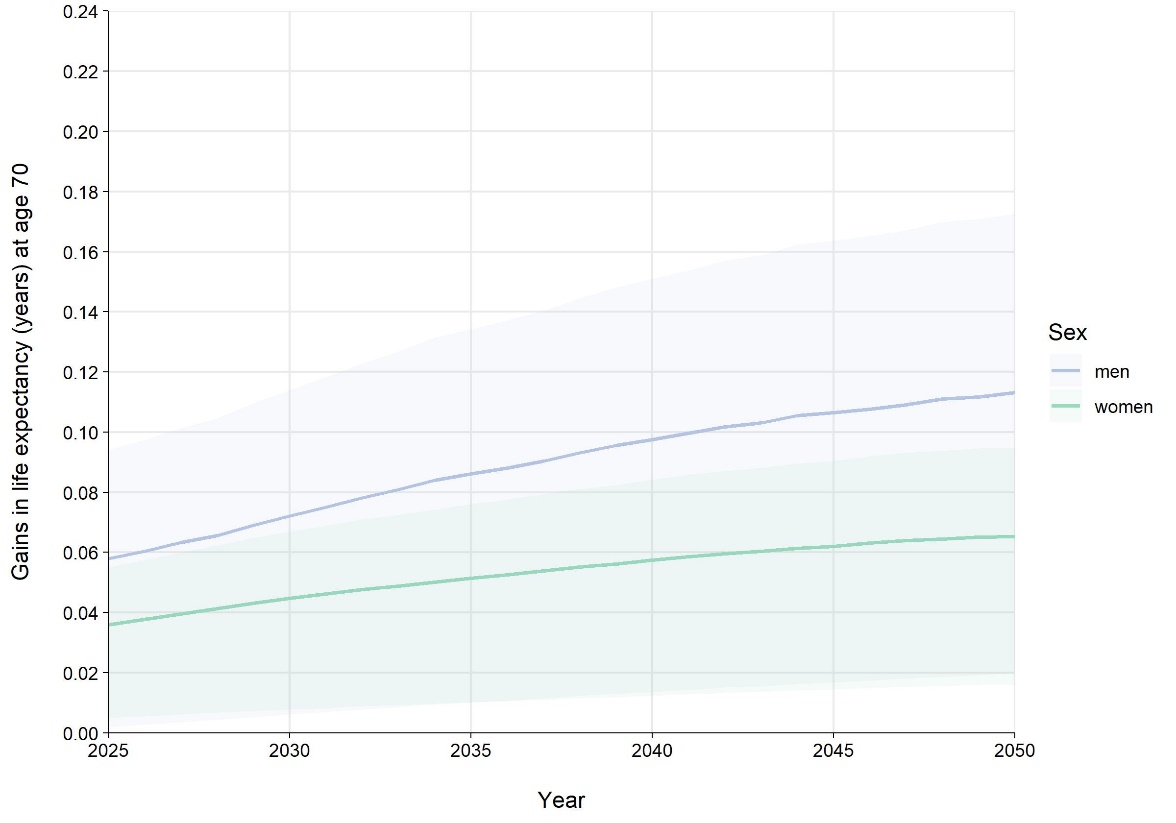


**(b2)** Vegetables, disease-free life expectancy


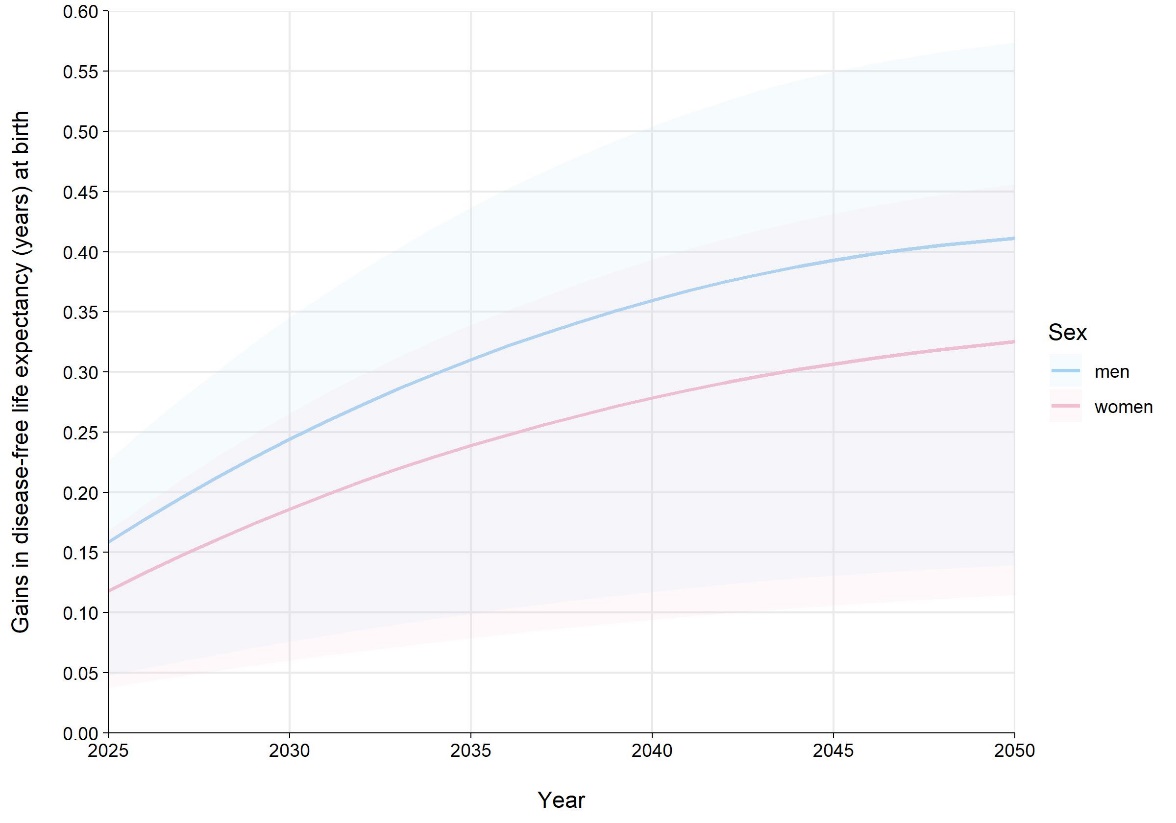


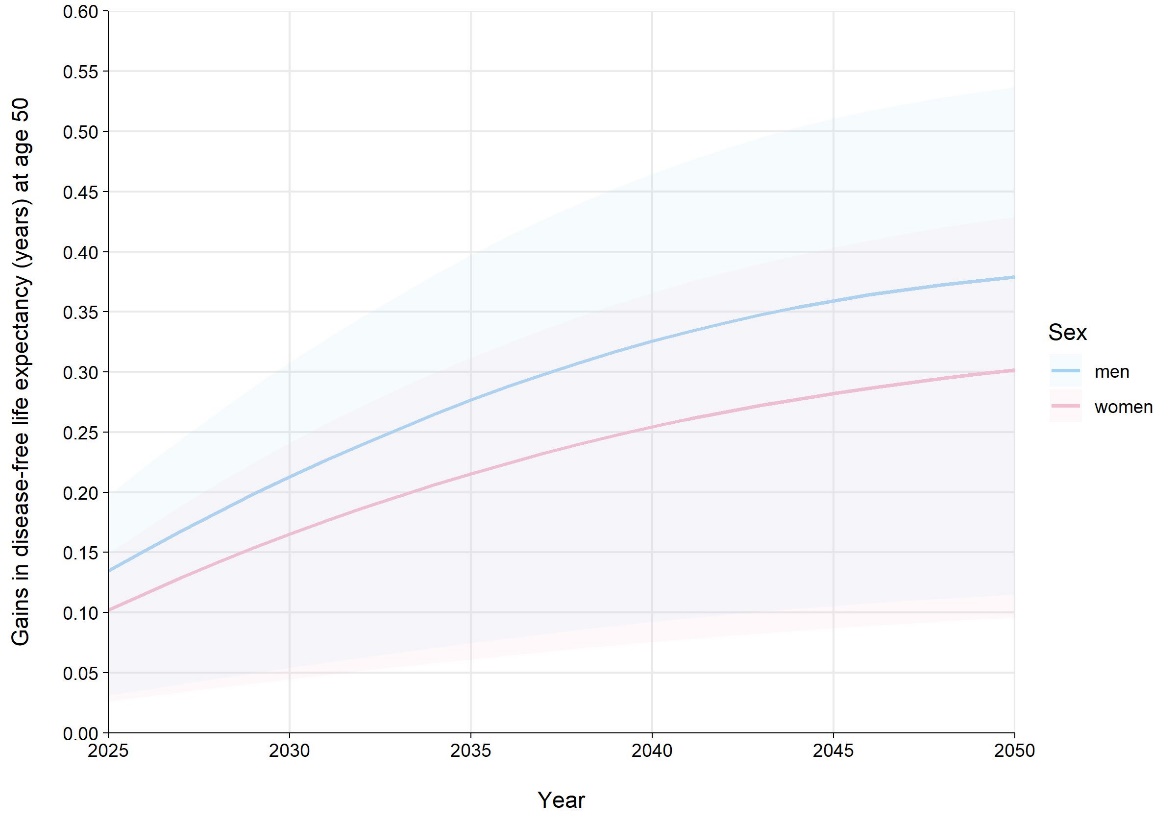


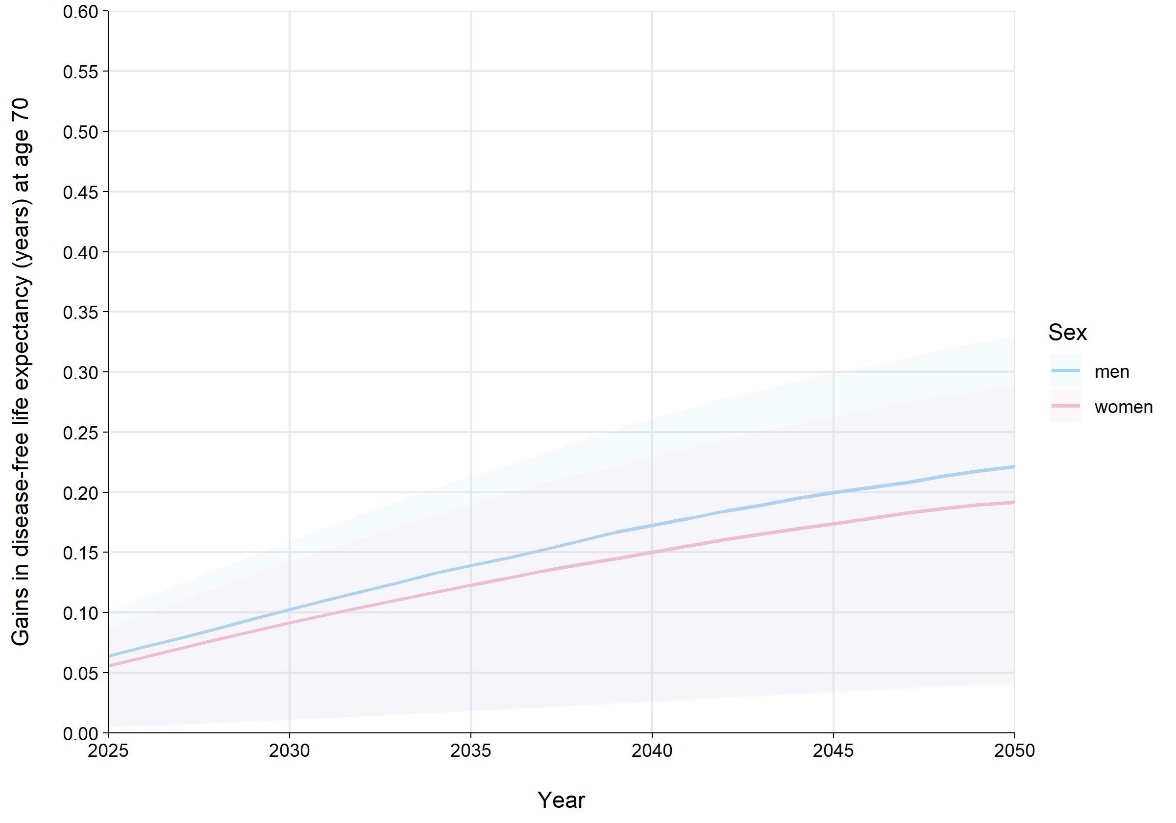


**(c1)** Whole grains, life expectancy


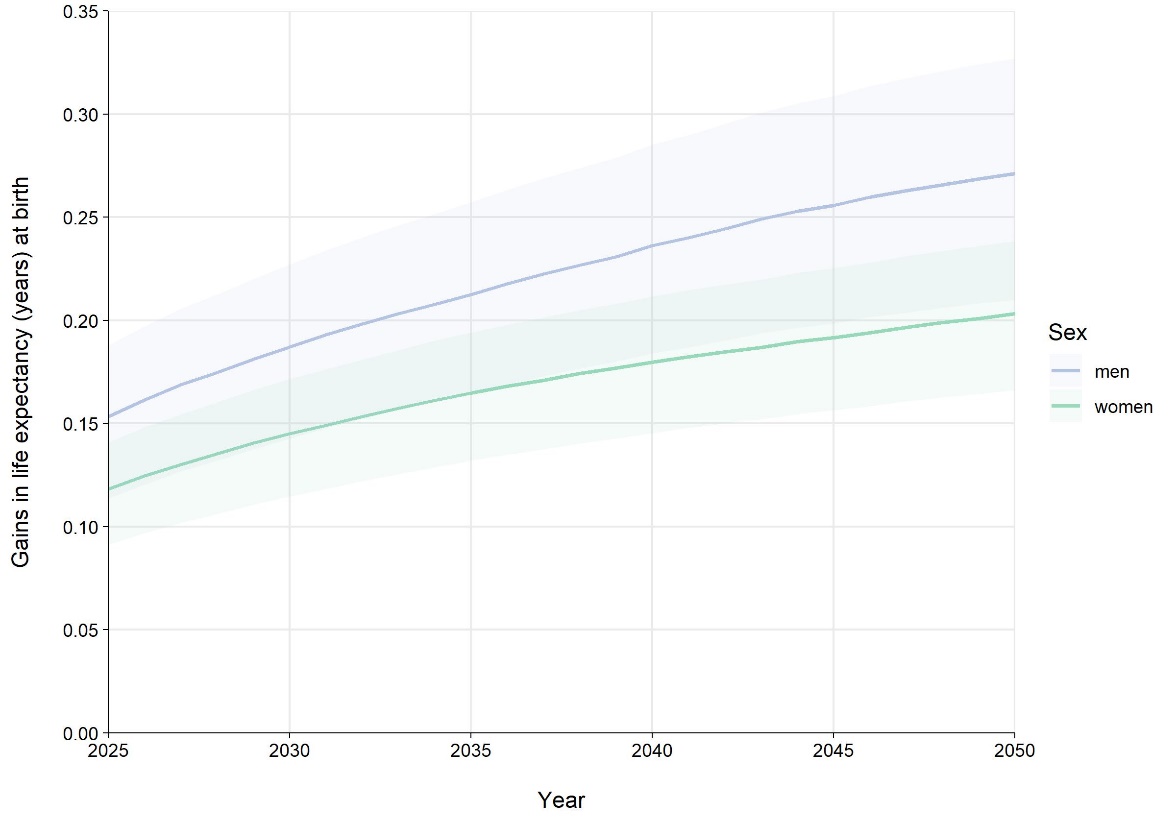


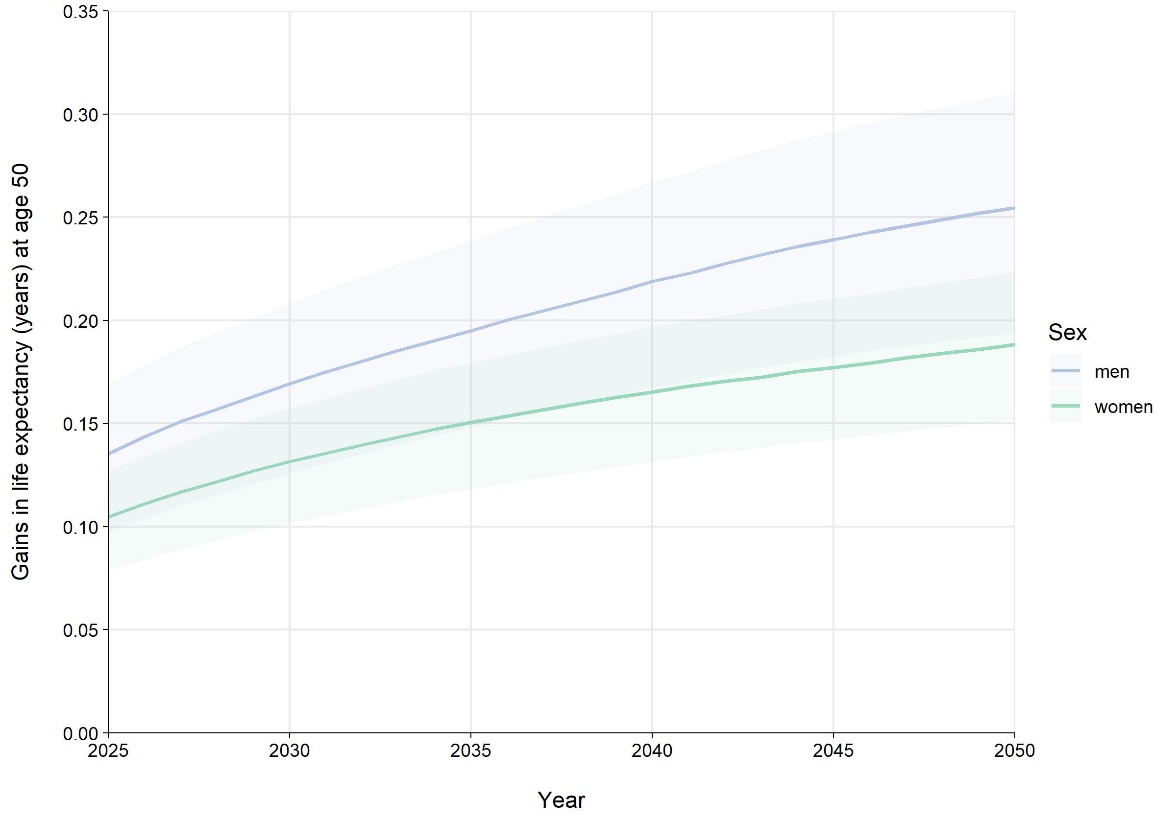


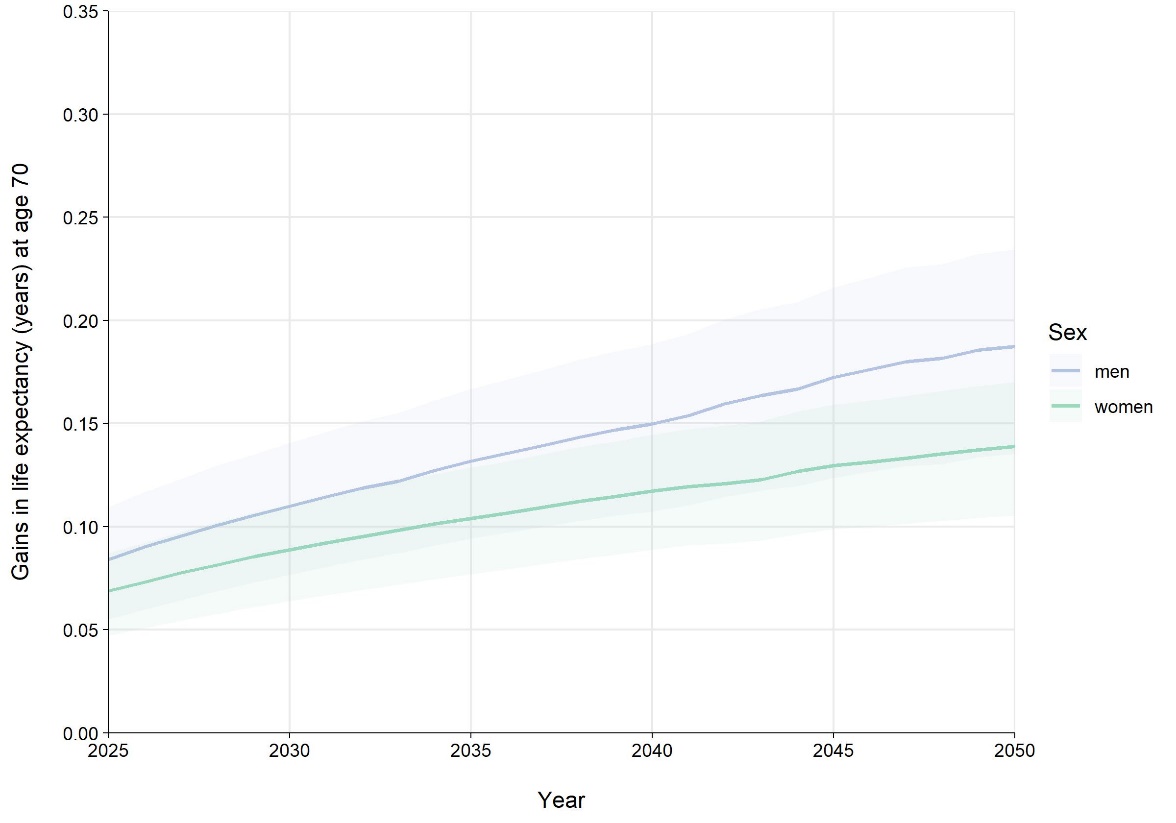


**(c2)** Whole grains, disease-free life expectancy


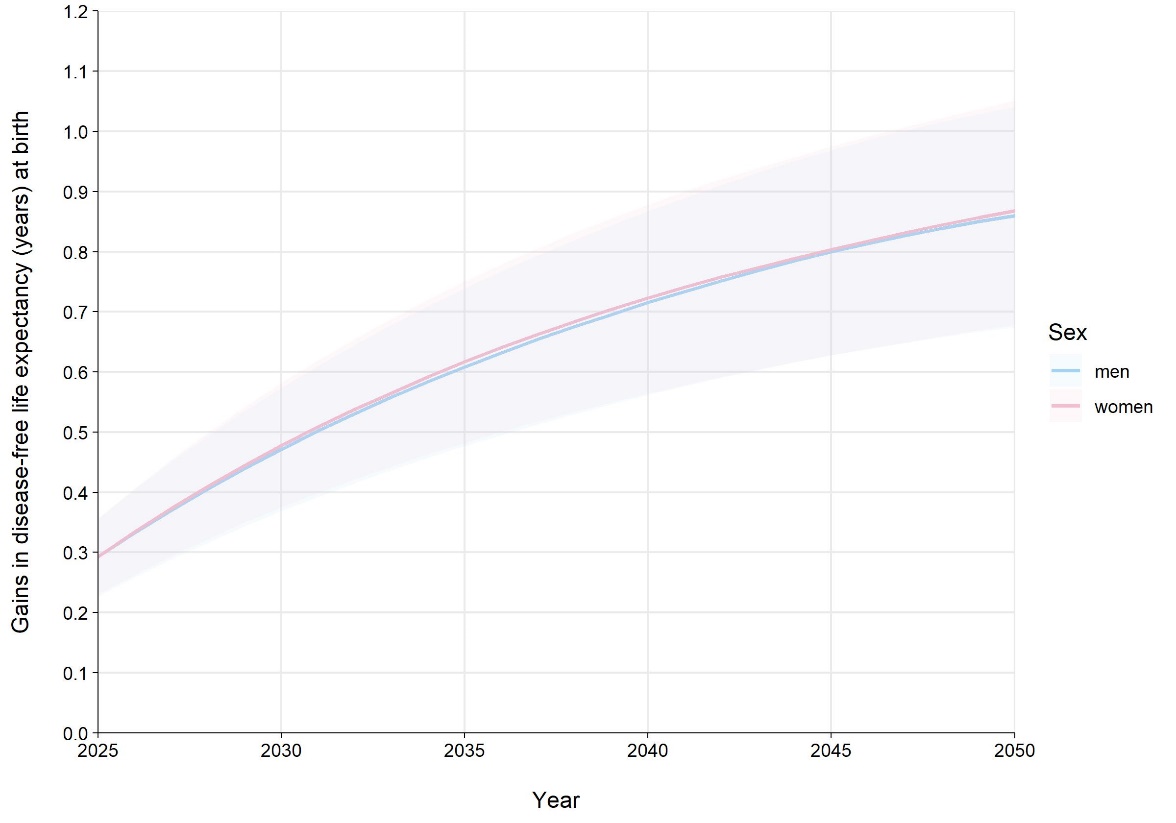


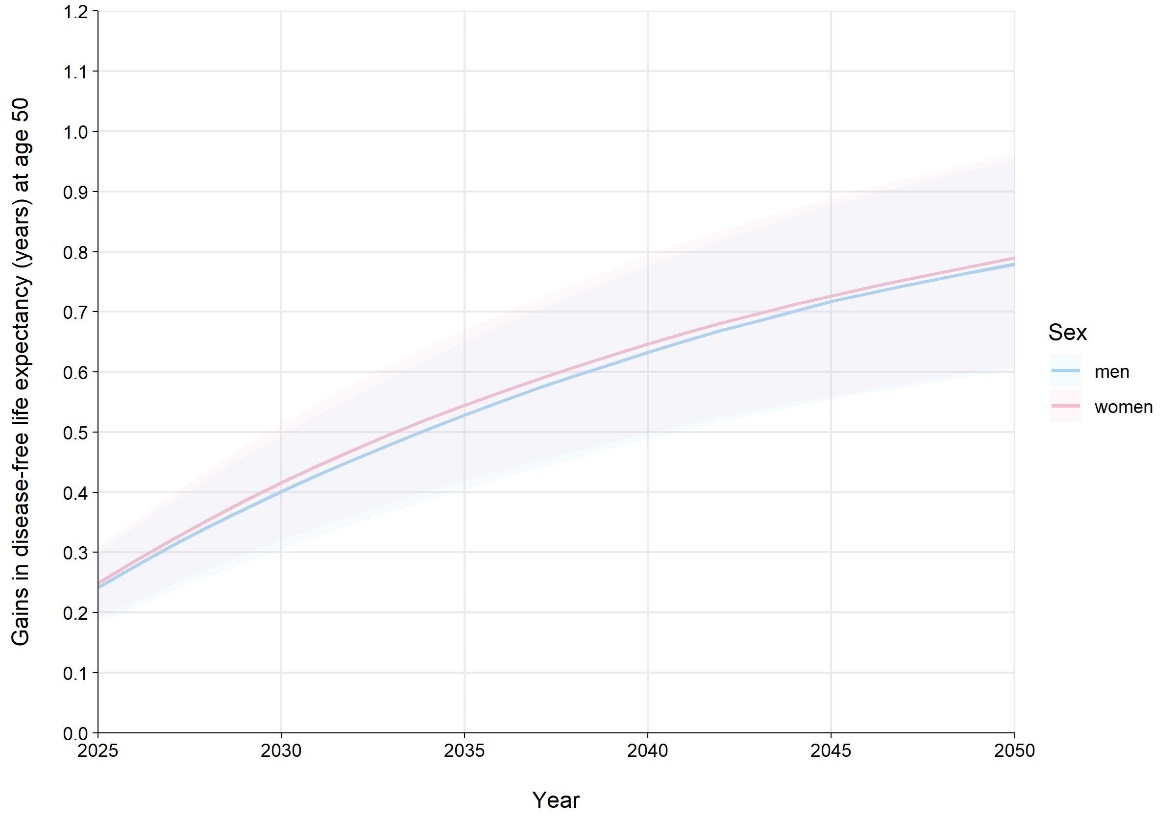


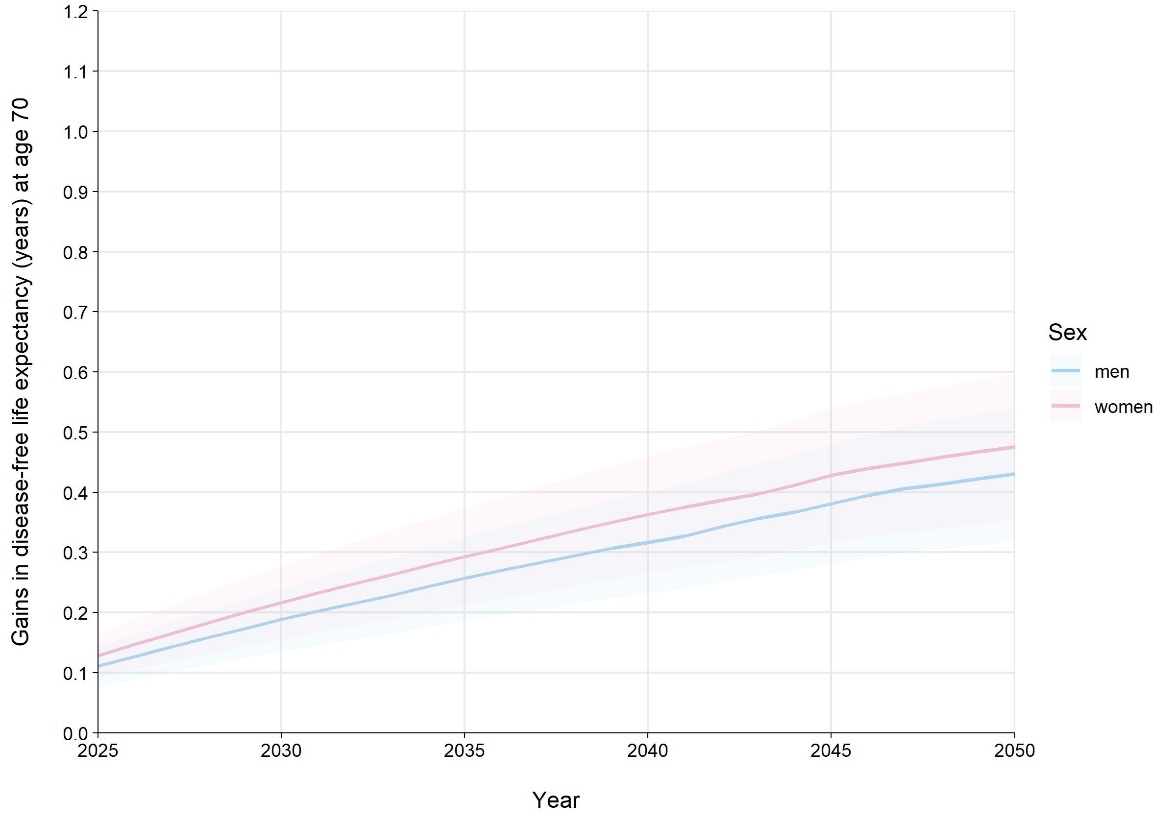


**(d1)** Nuts/seeds, life expectancy


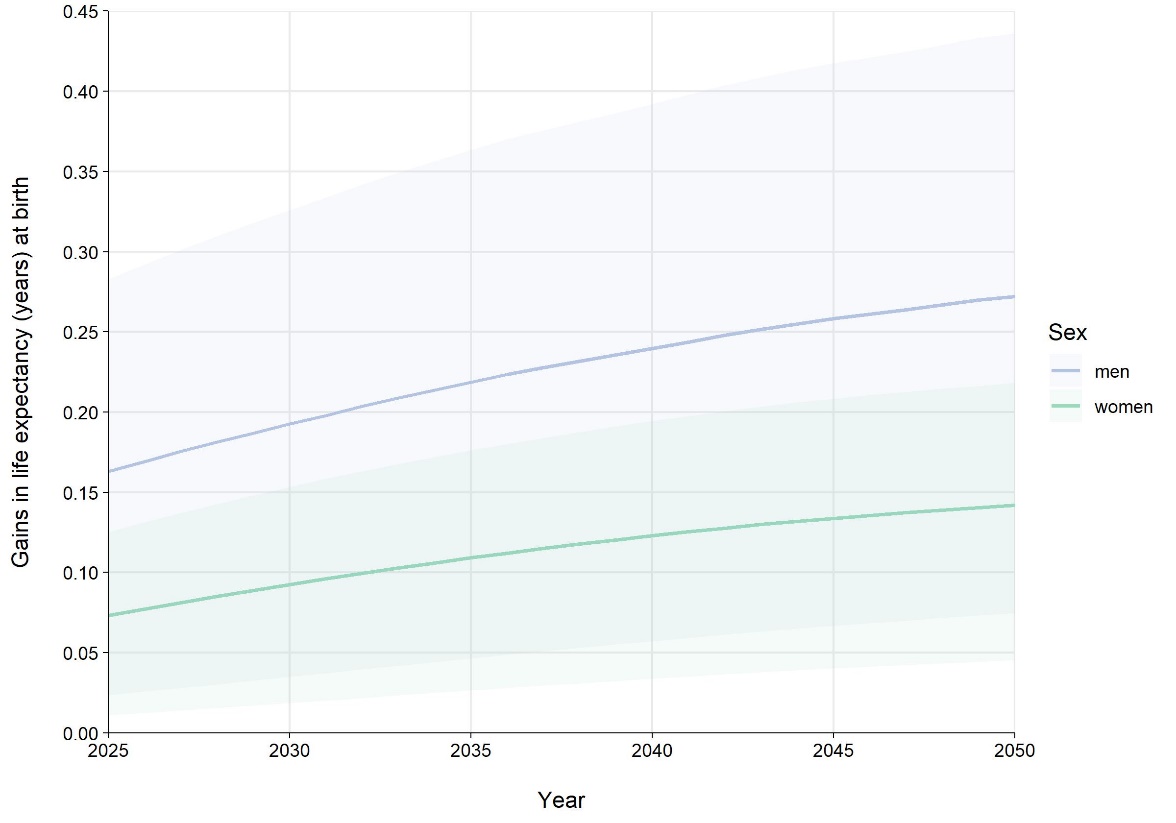


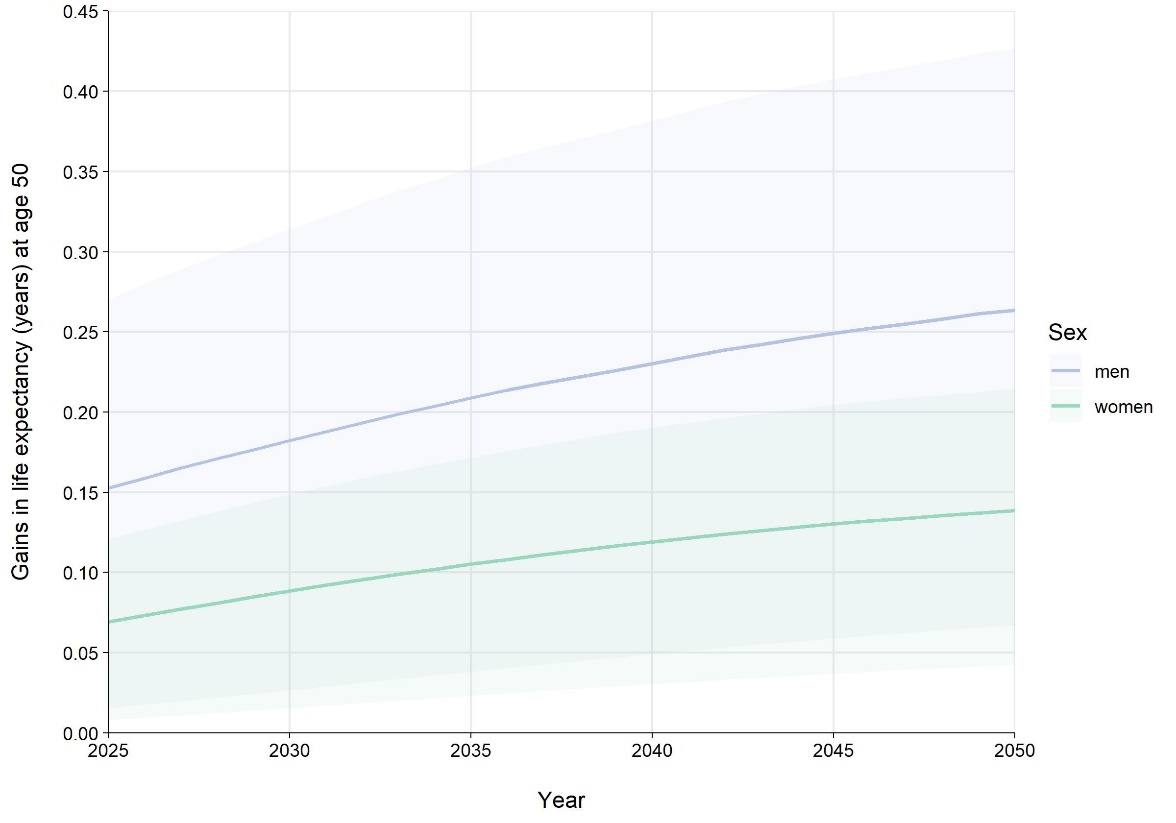


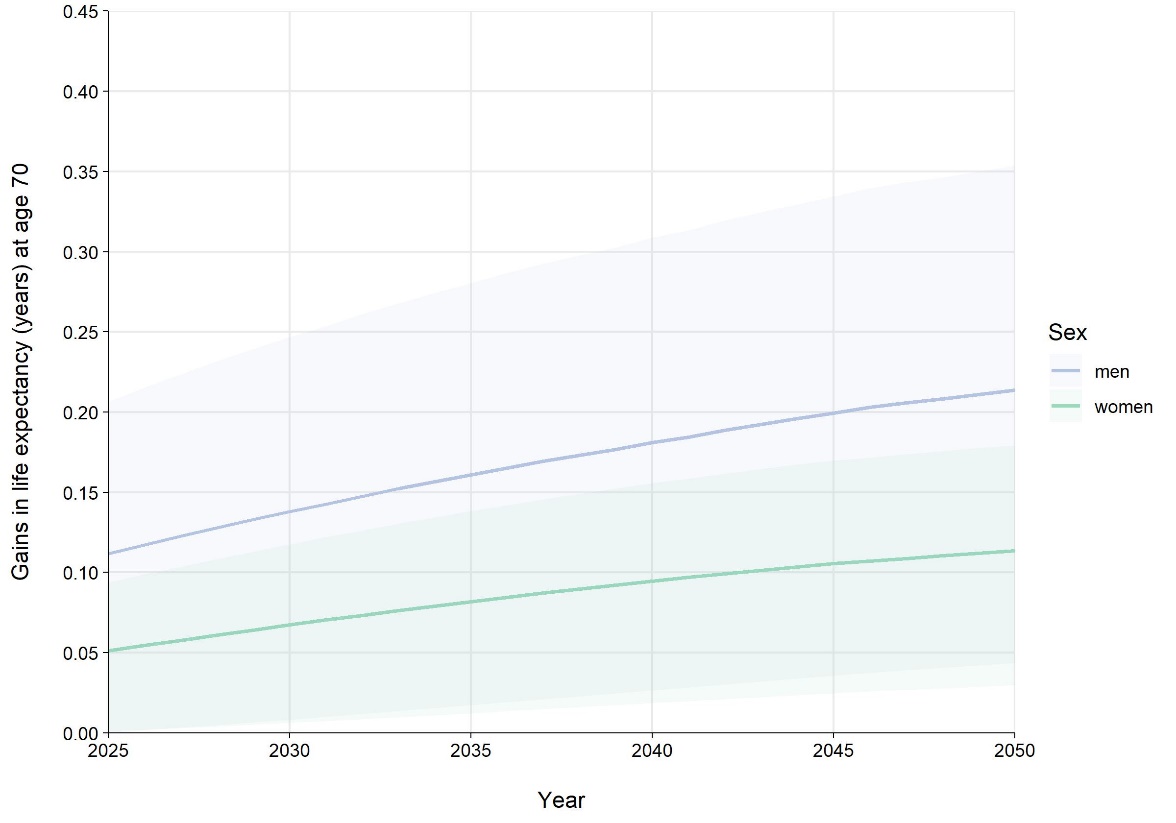


**(d2)** Nuts/seeds, disease-free life expectancy


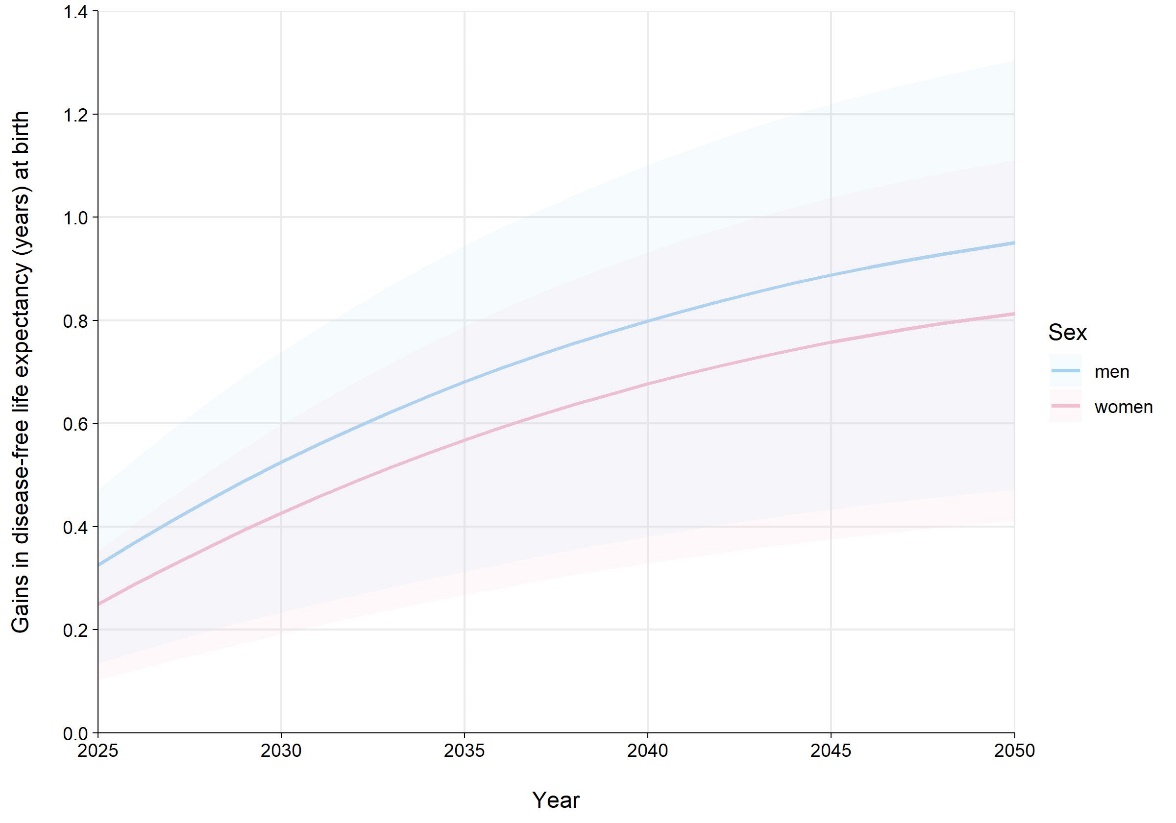


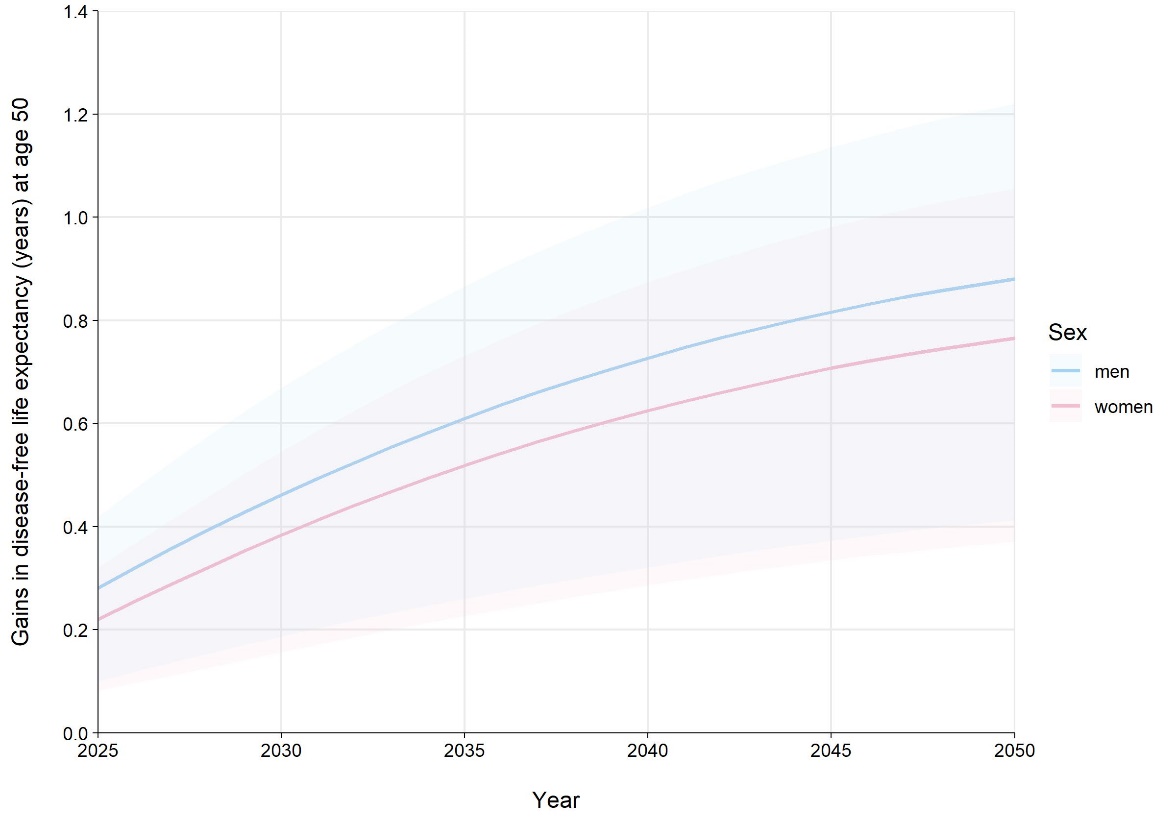


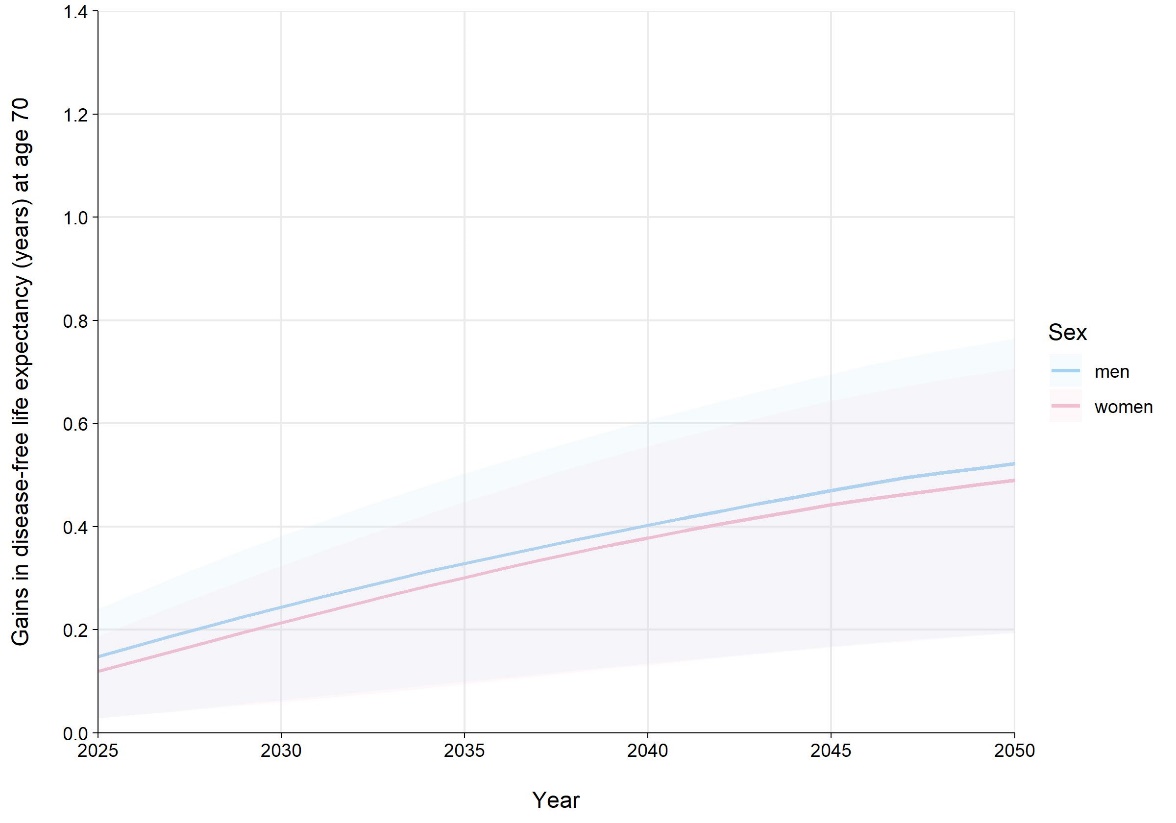


**(e1)** Legumes, life expectancy


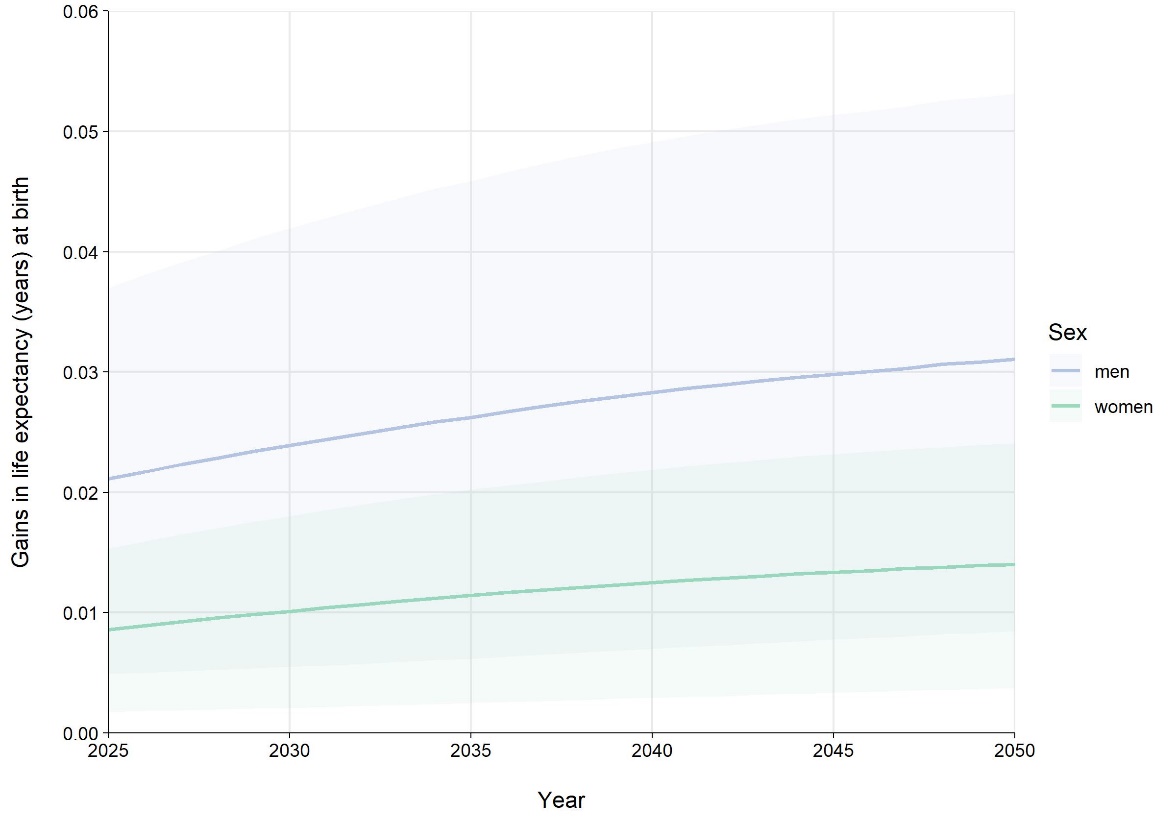


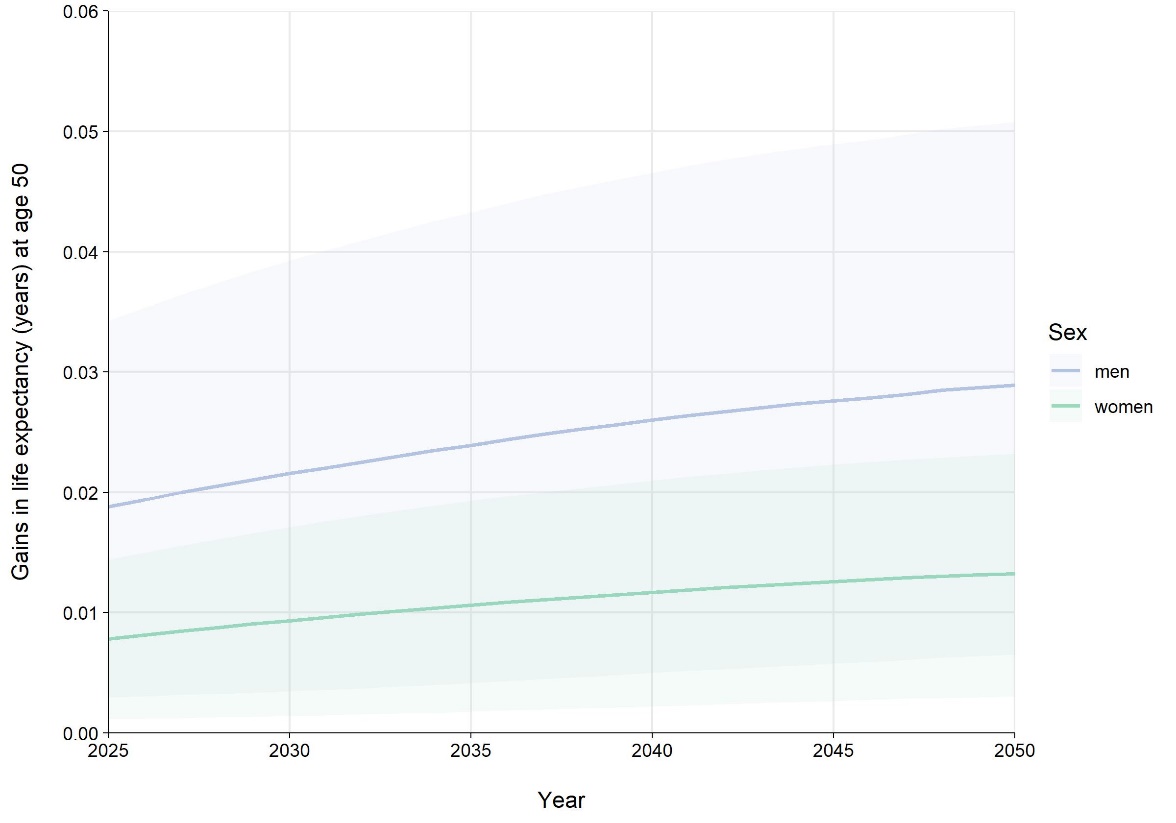


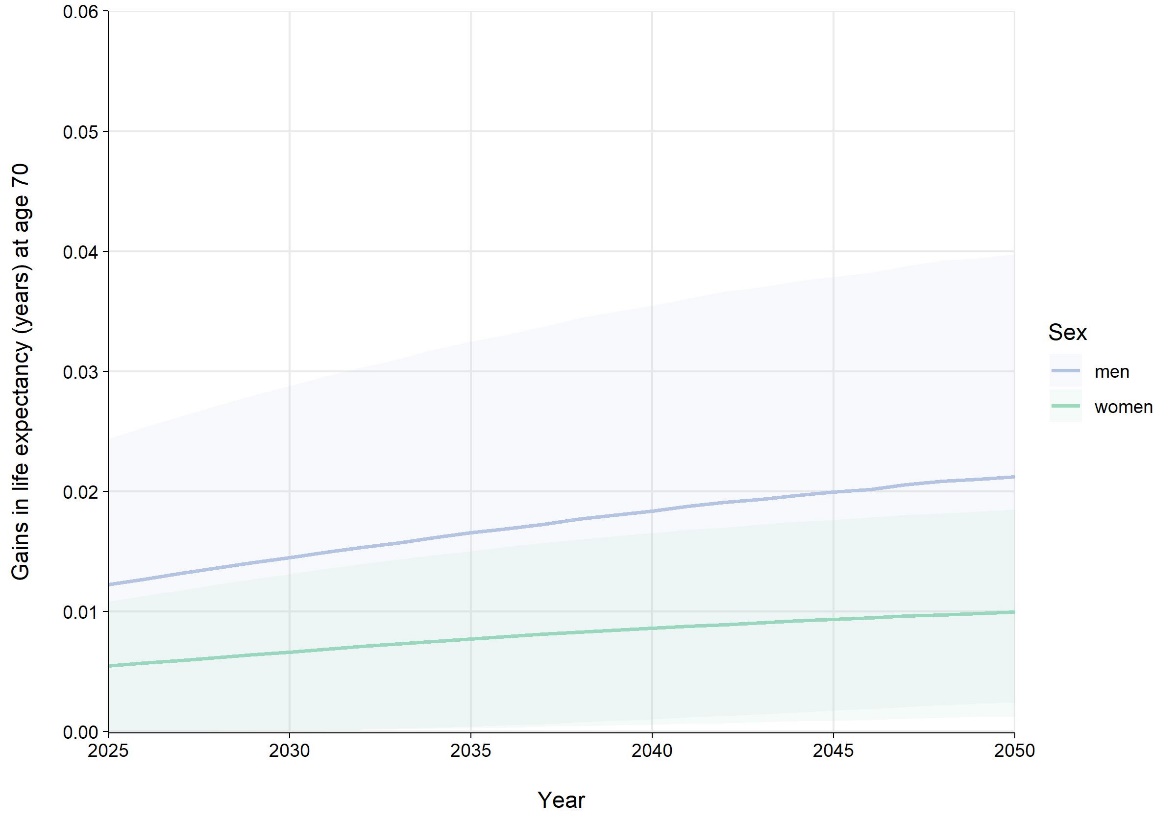


**(e2)** Legumes, disease-free life expectancy


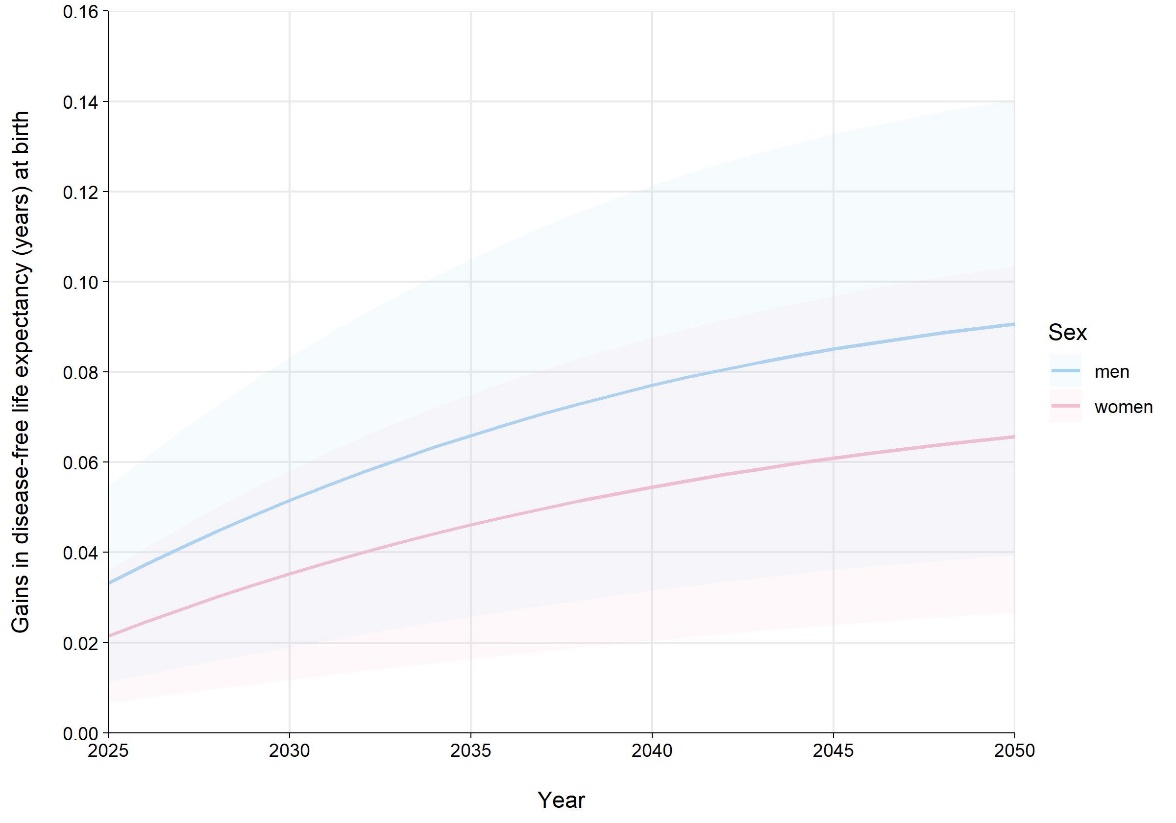


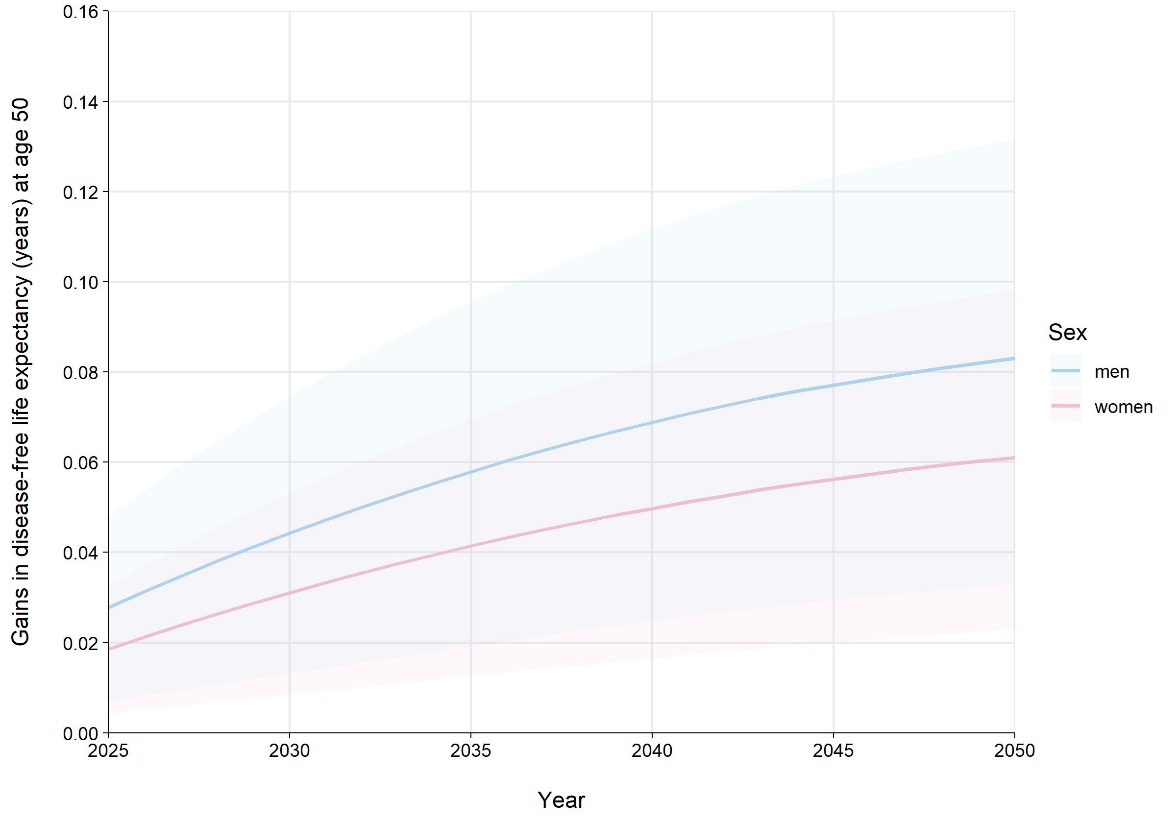


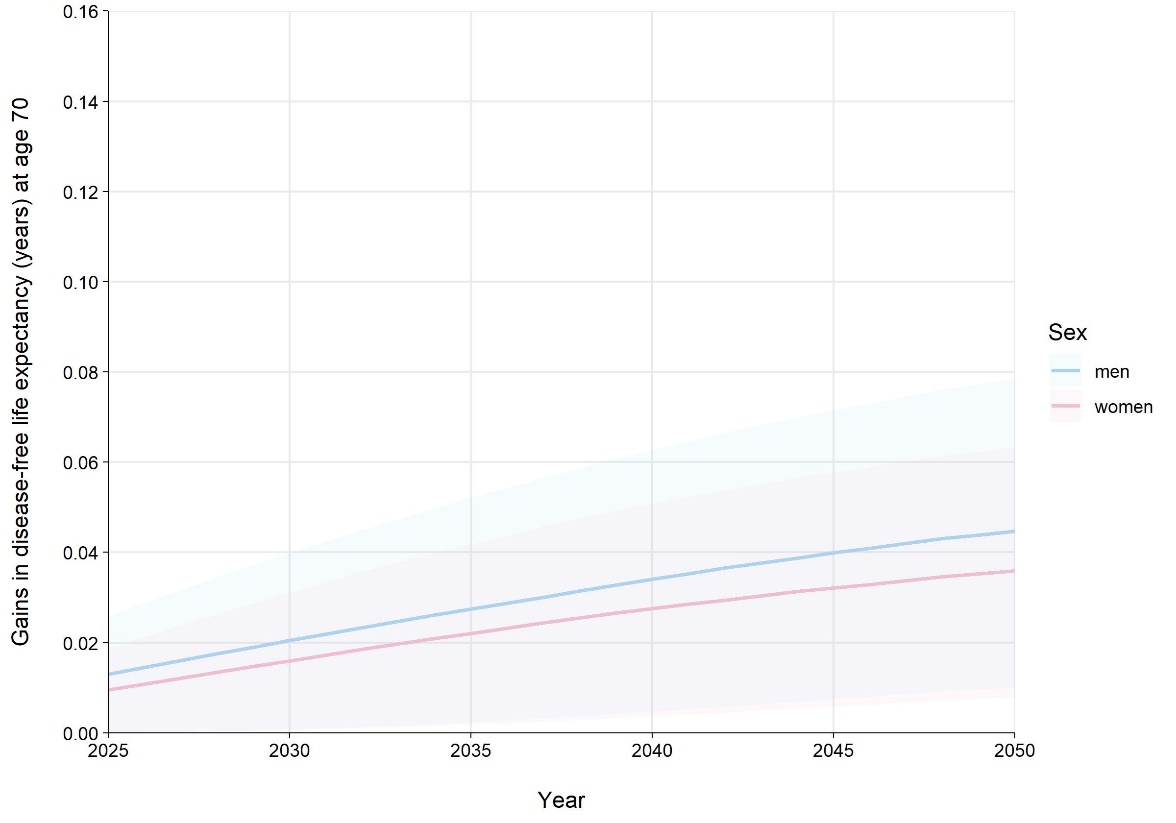


**(f1)** Fish, life expectancy


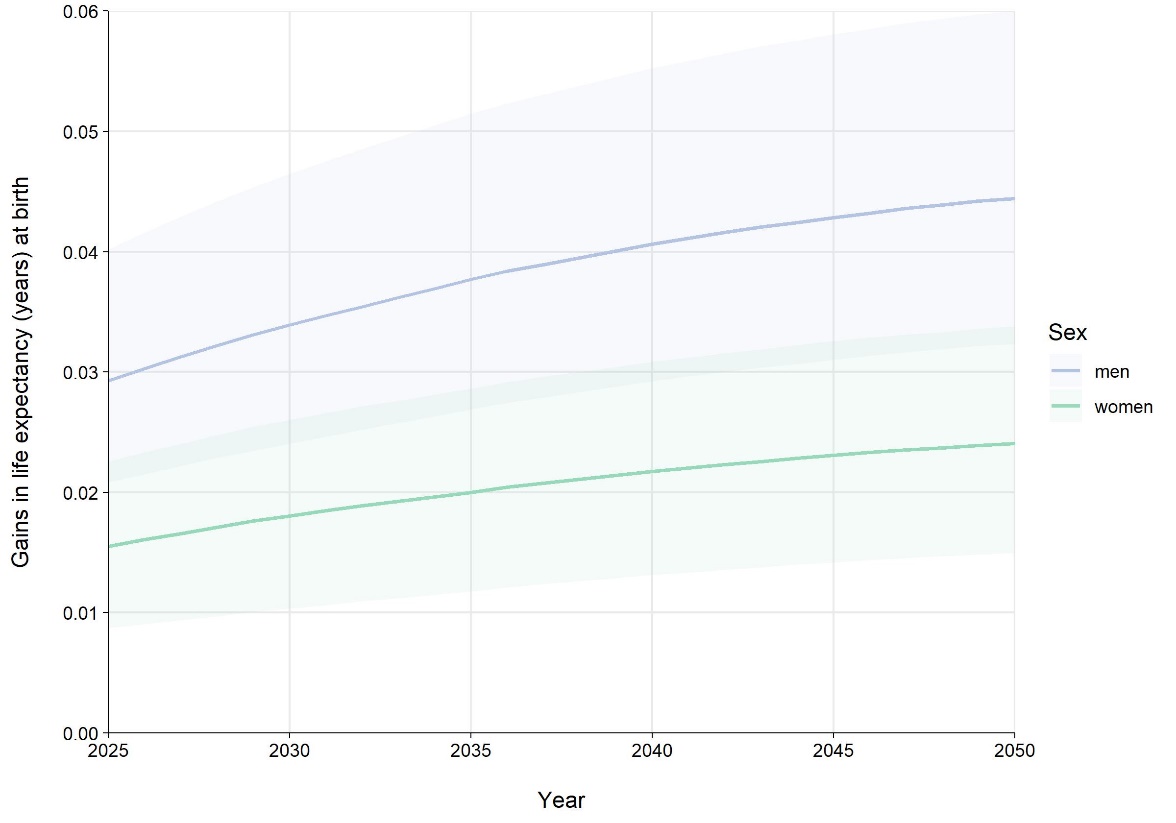


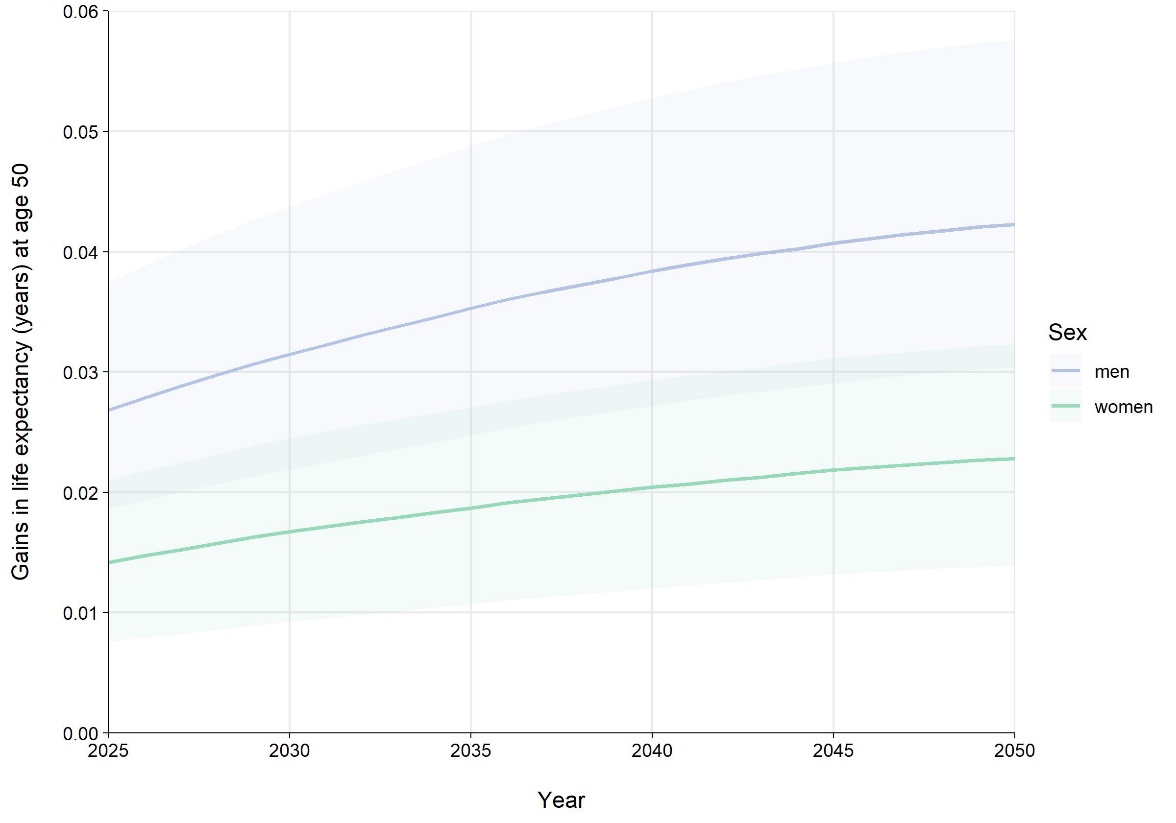


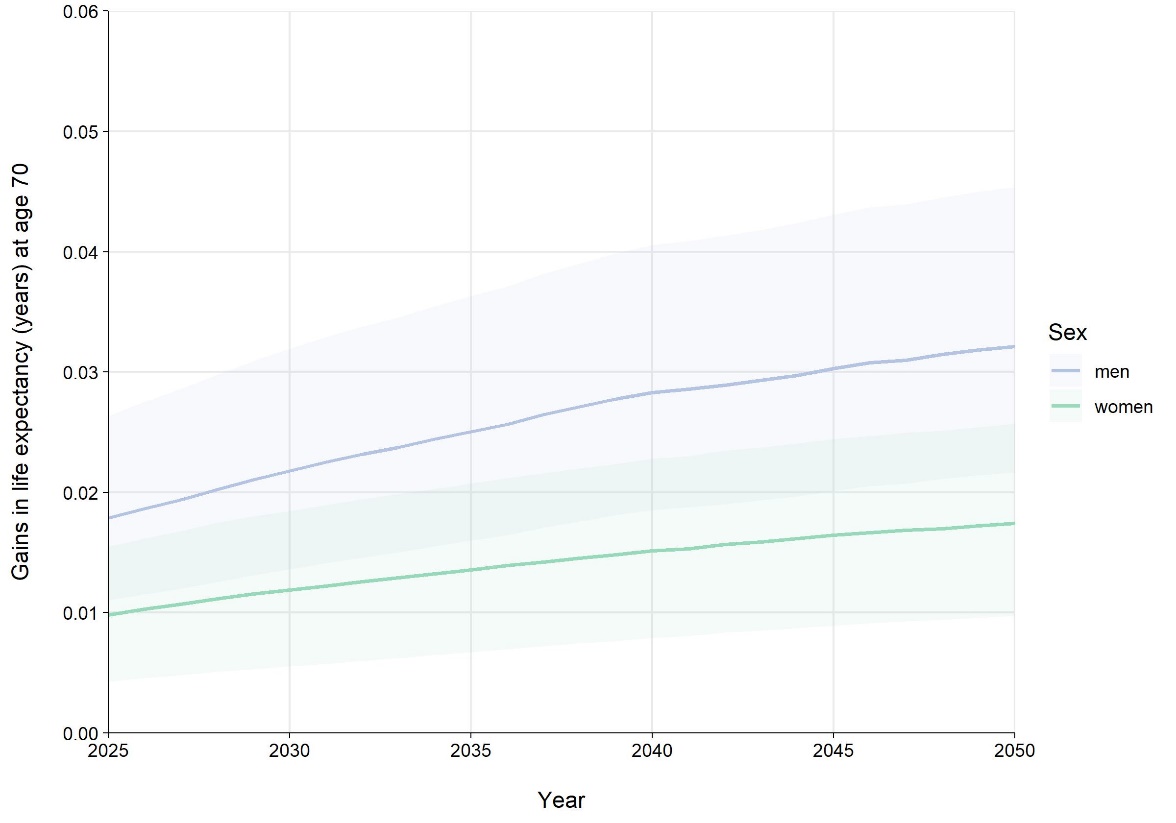


**(f2)** Fish, disease-free life expectancy


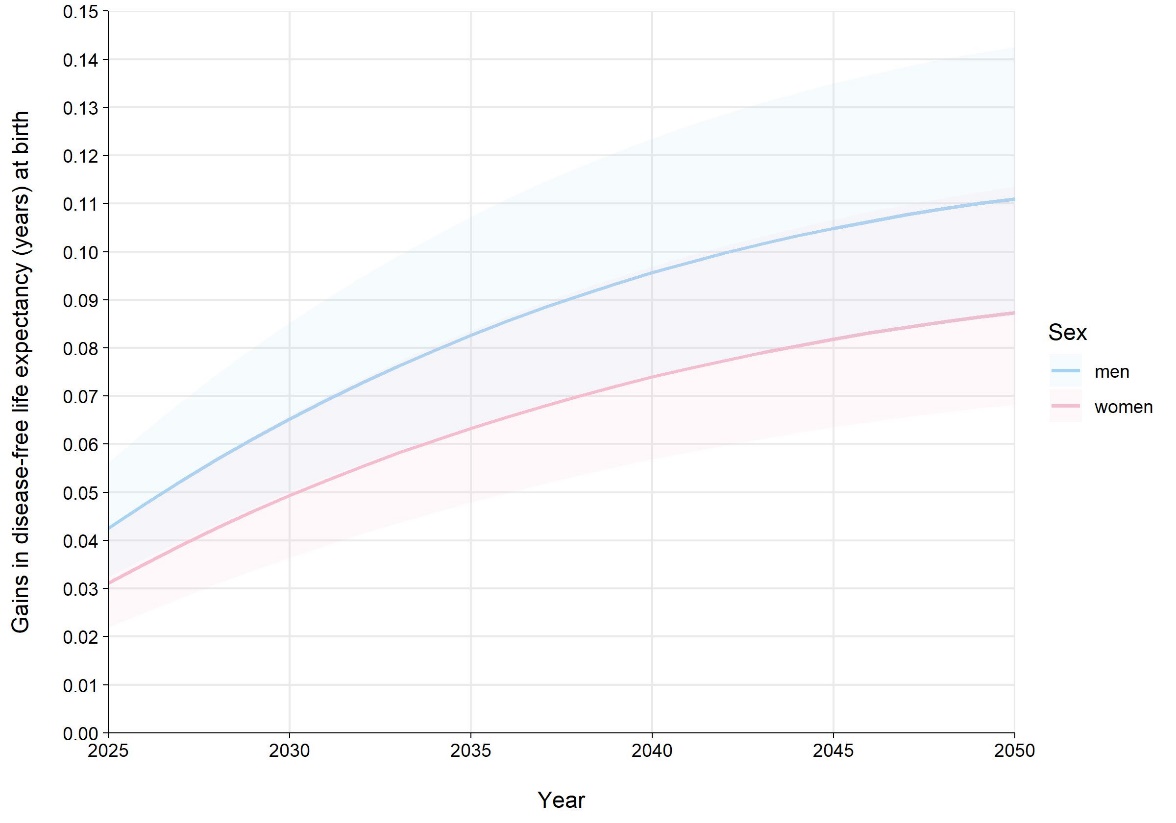


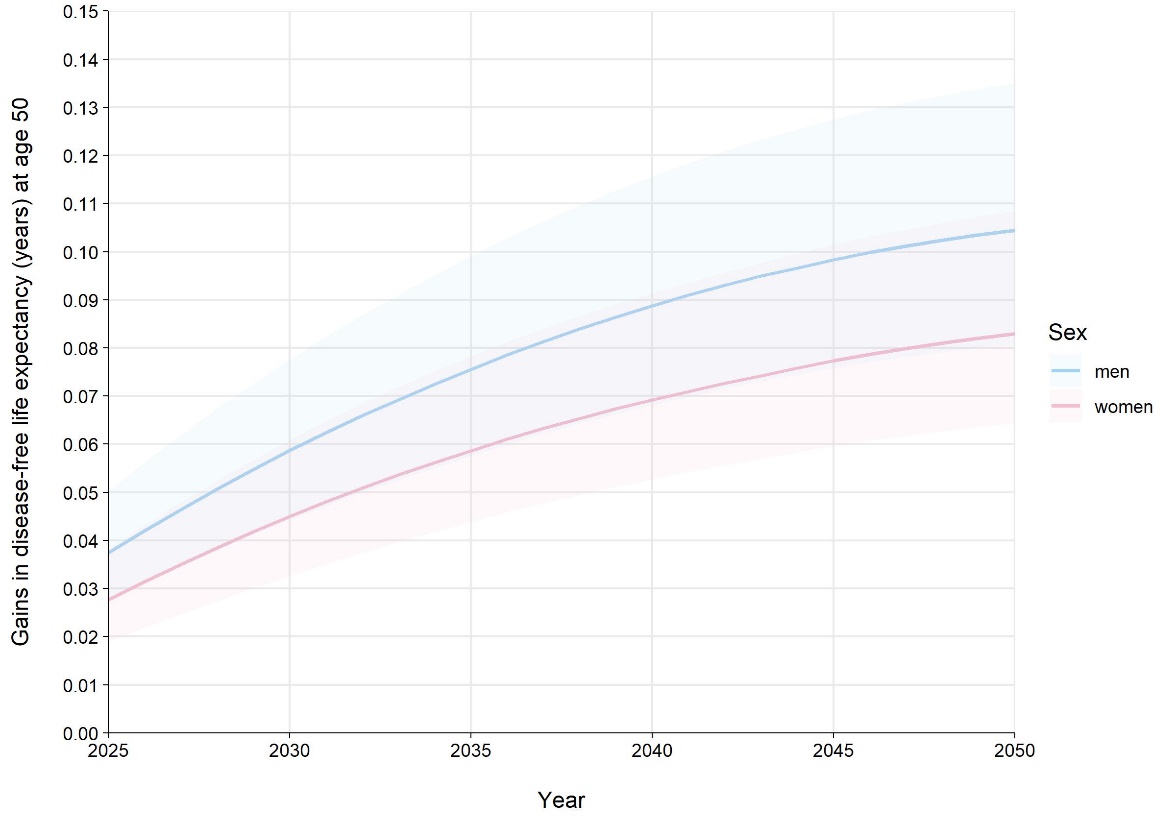


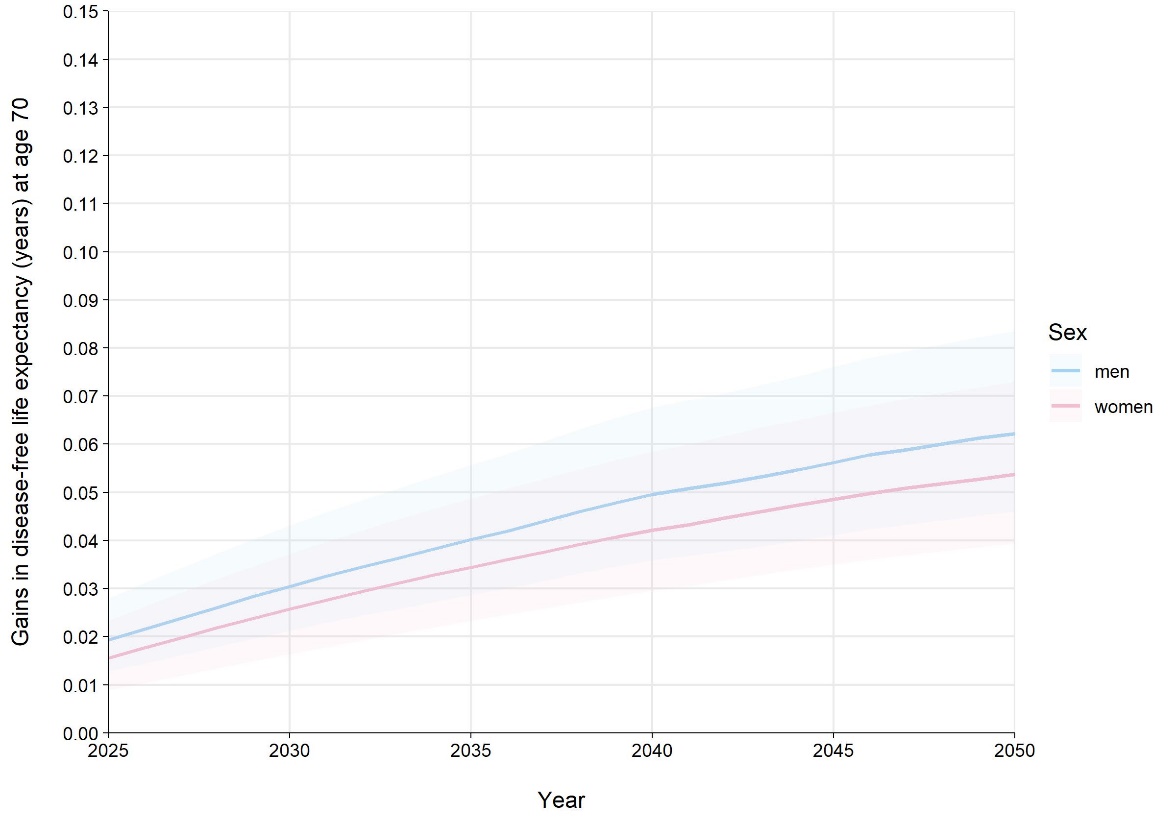


**(g1)** Red meat, life expectancy


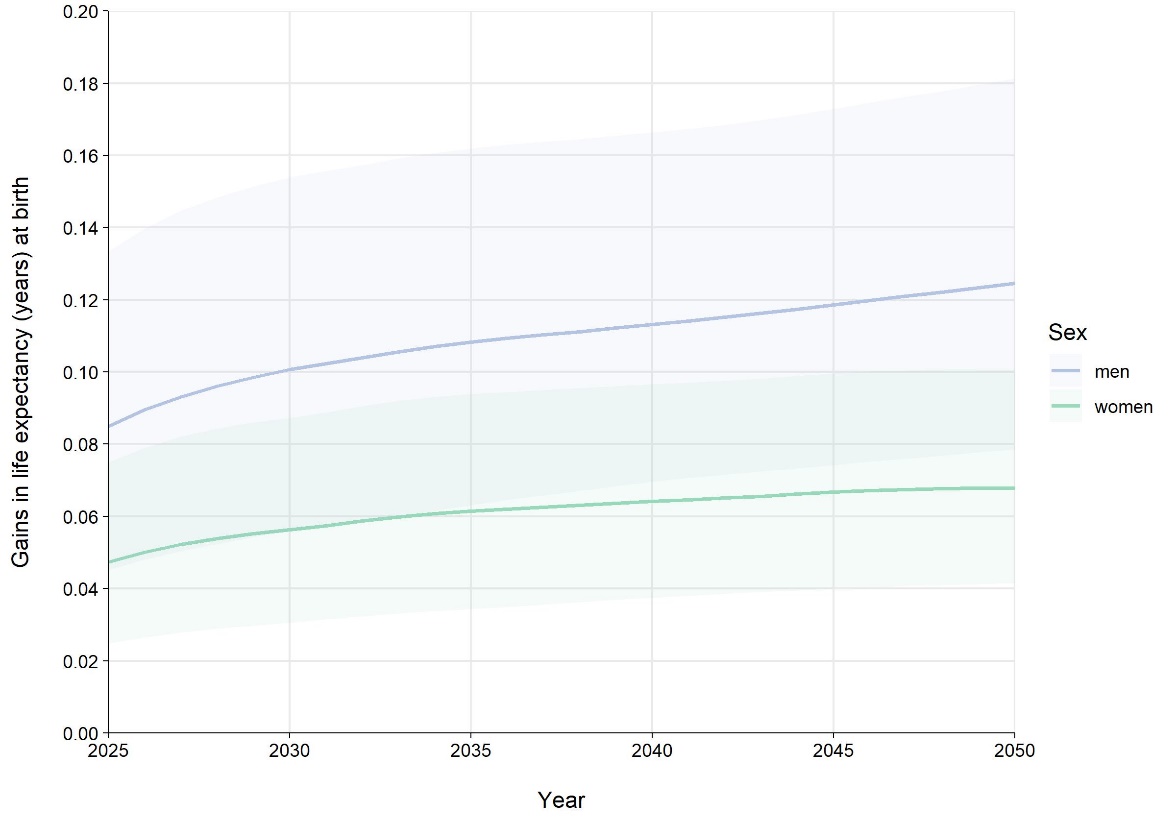


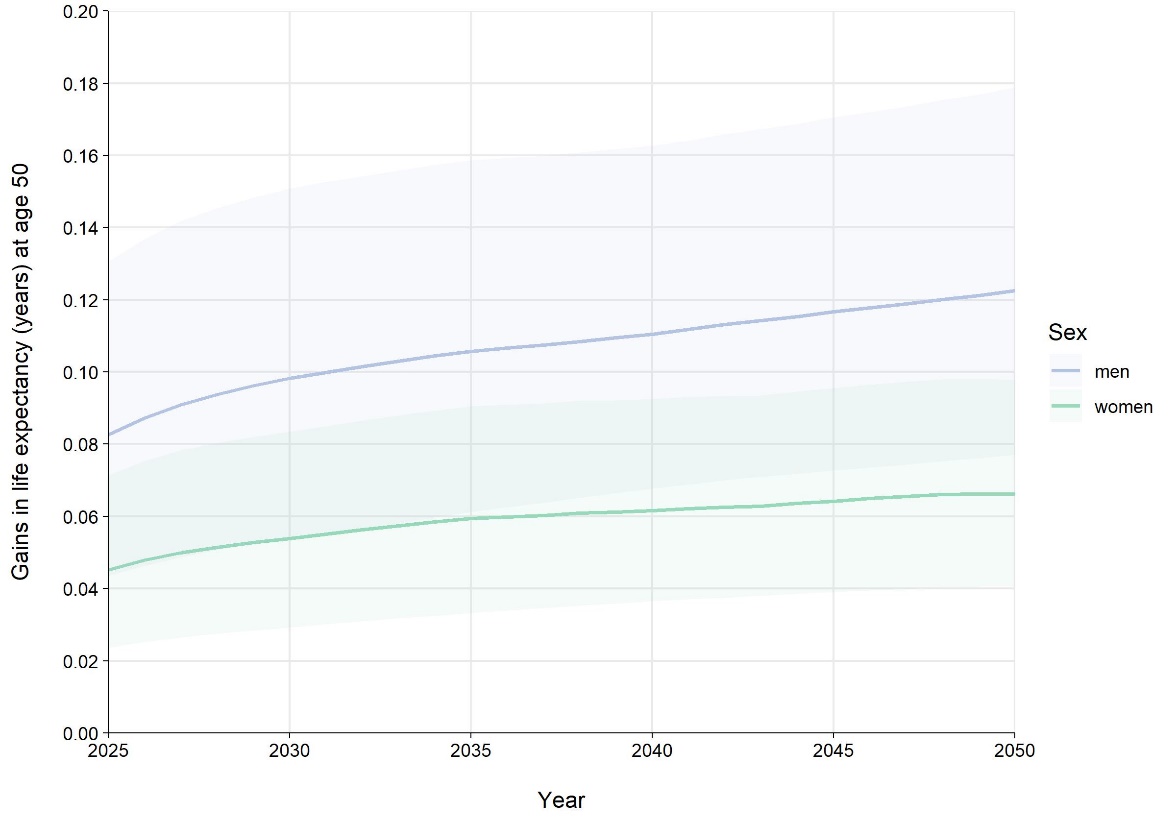


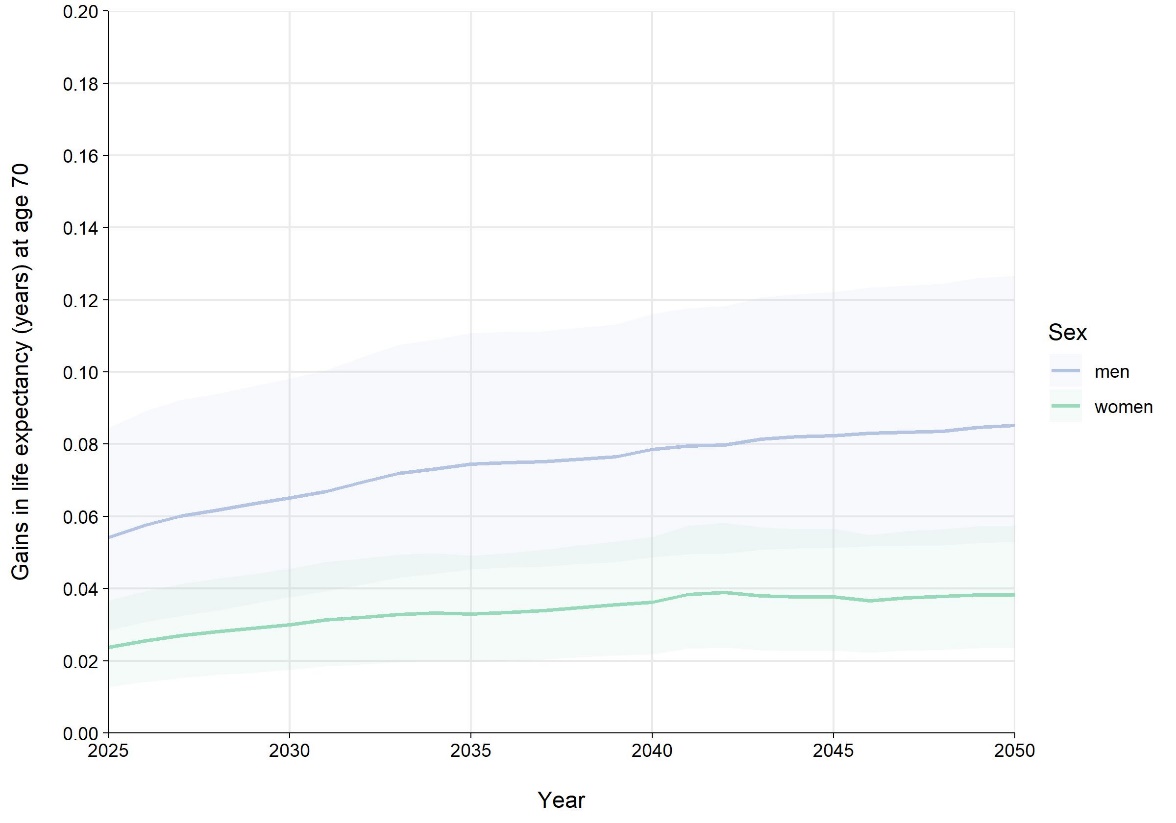


**(g2)** Red meat, disease-free life expectancy


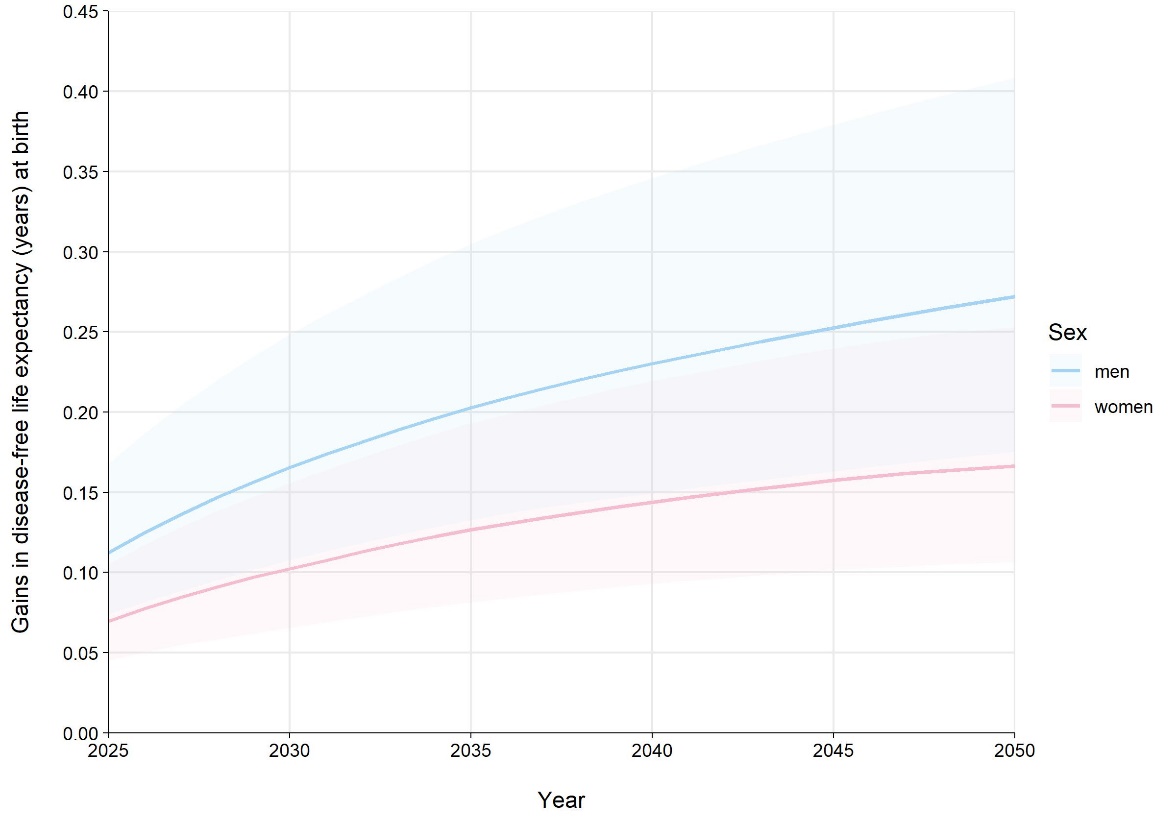


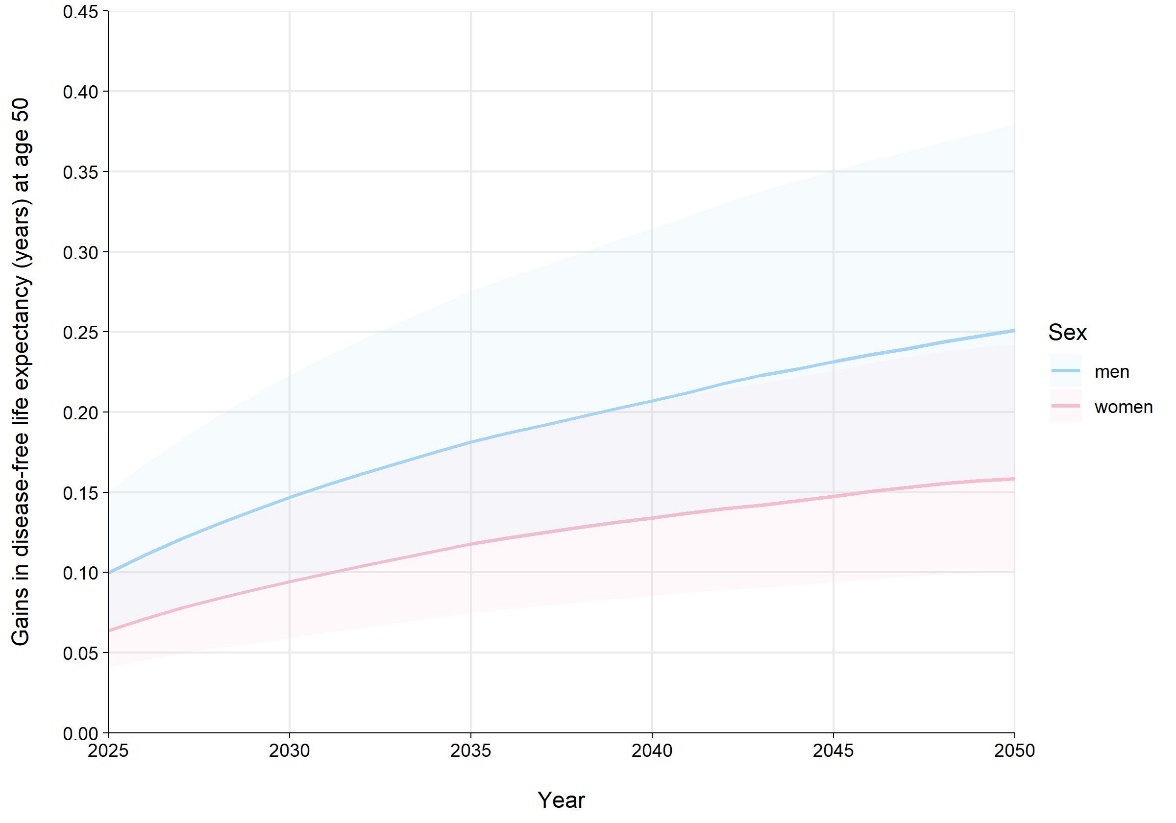


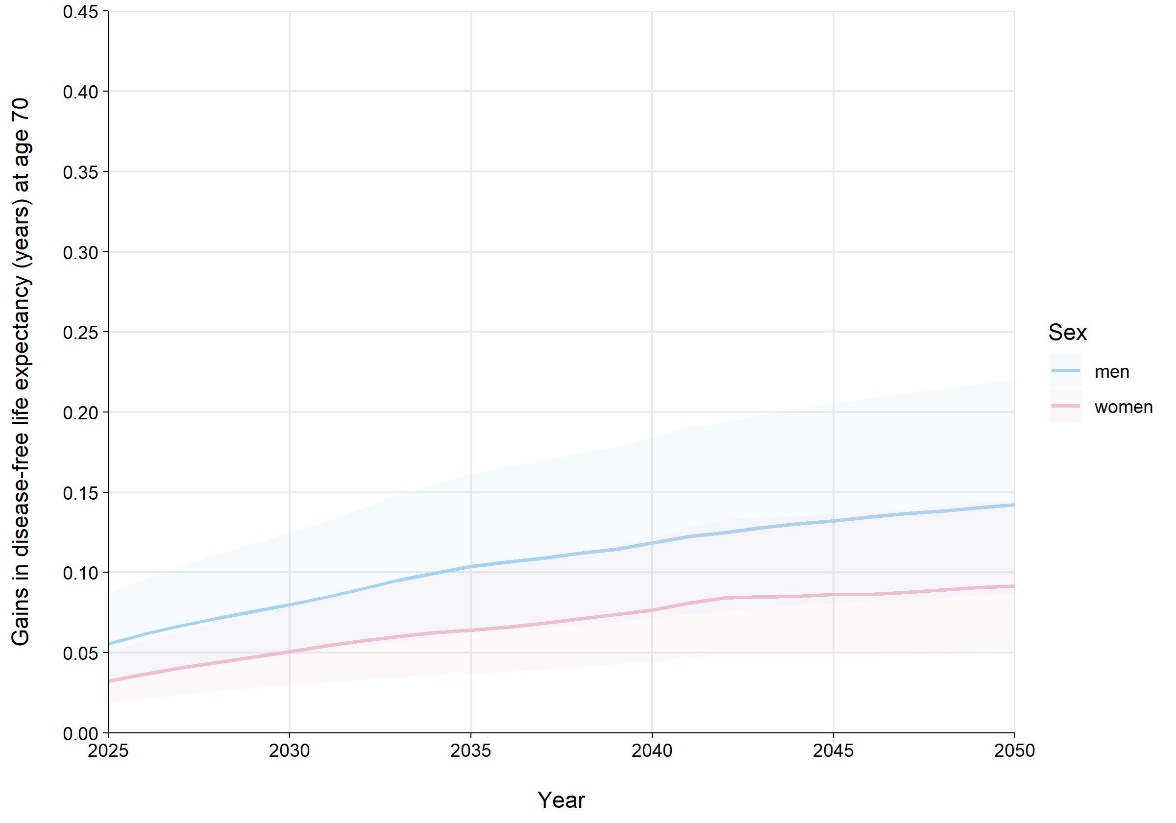


**(h1)** Processed meat, life expectancy


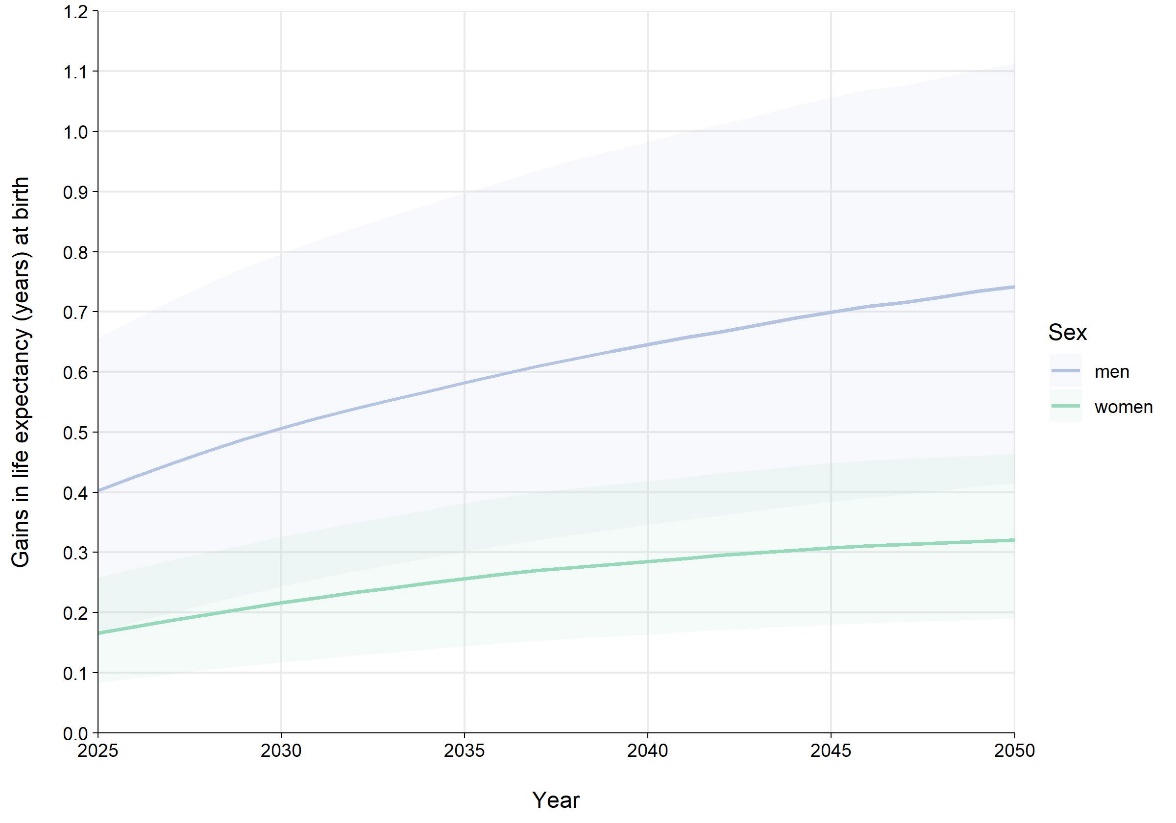


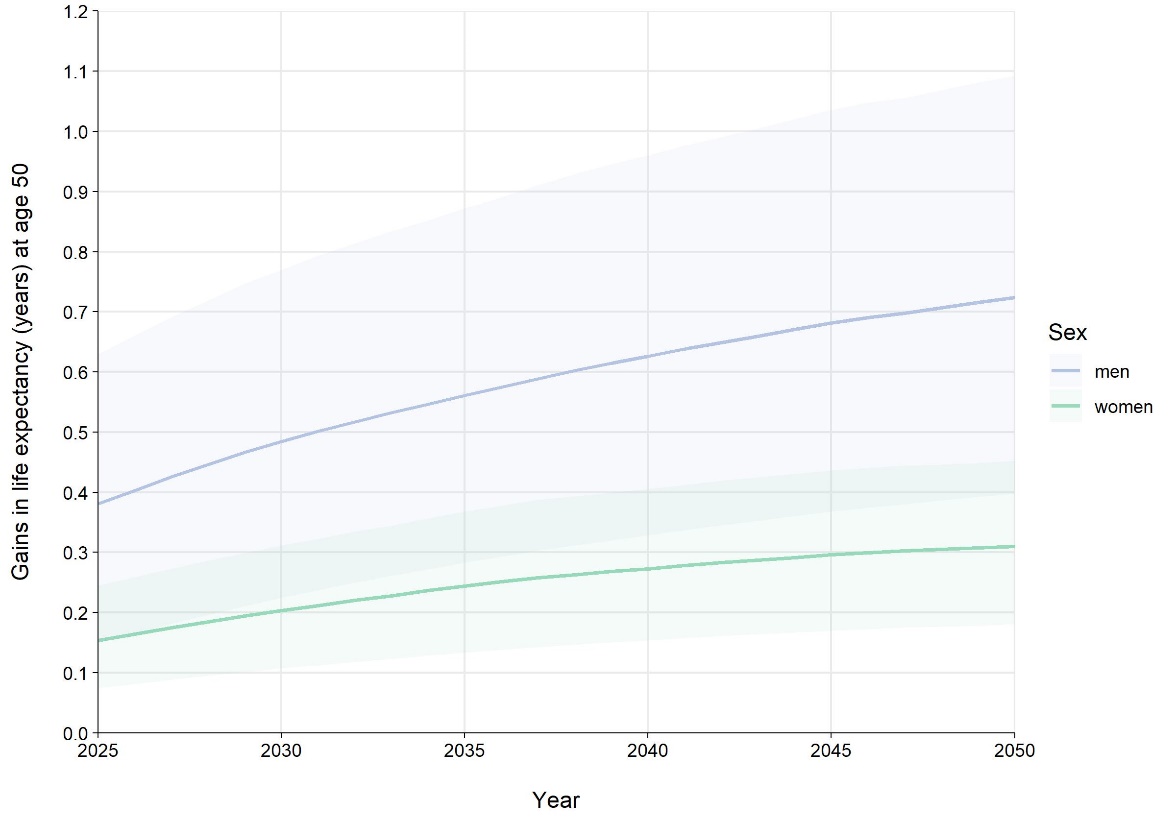


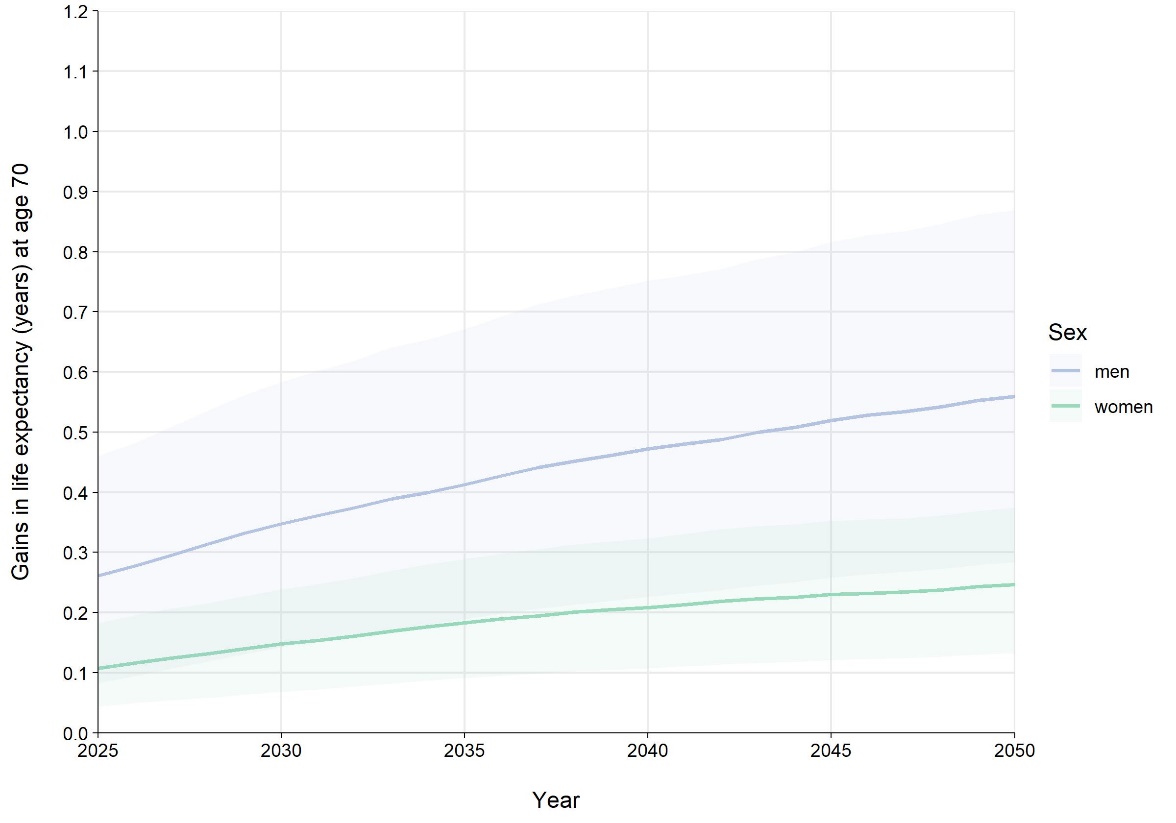


**(h2)** Processed meat, disease-free life expectancy


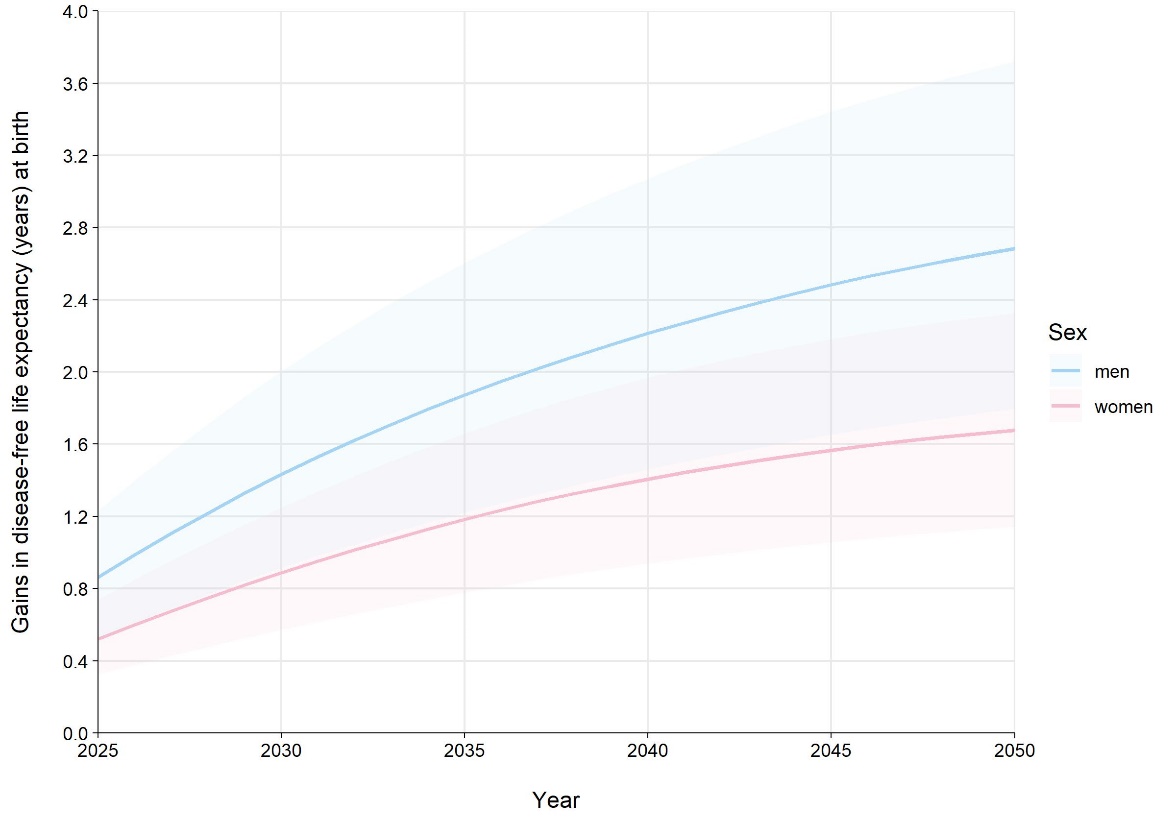


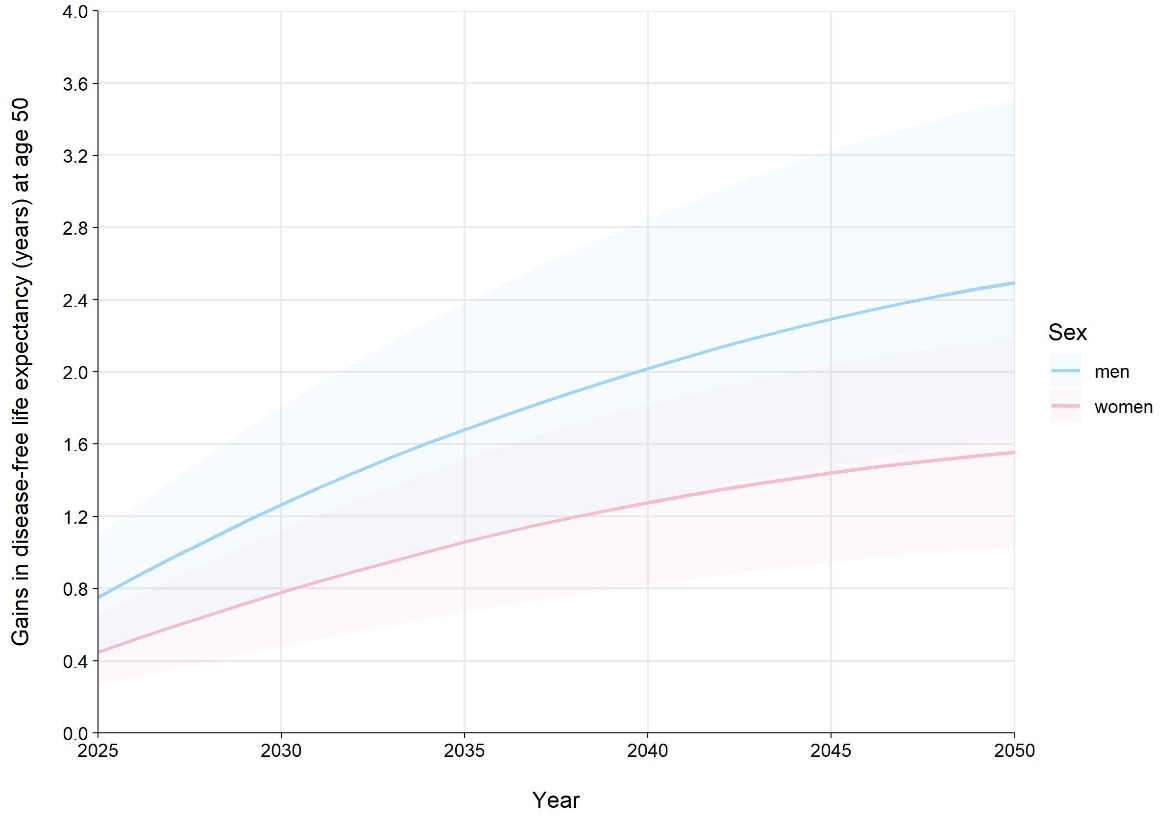


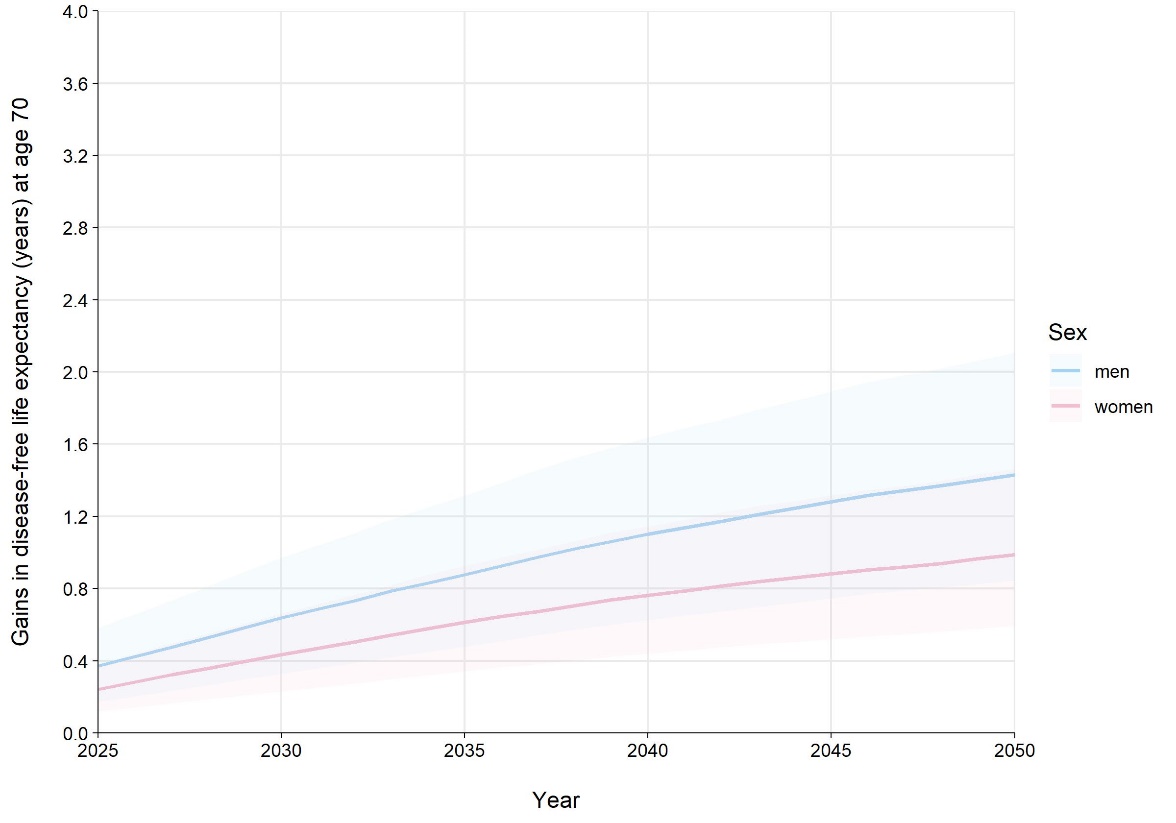


**(i1)** Dairy products, life expectancy


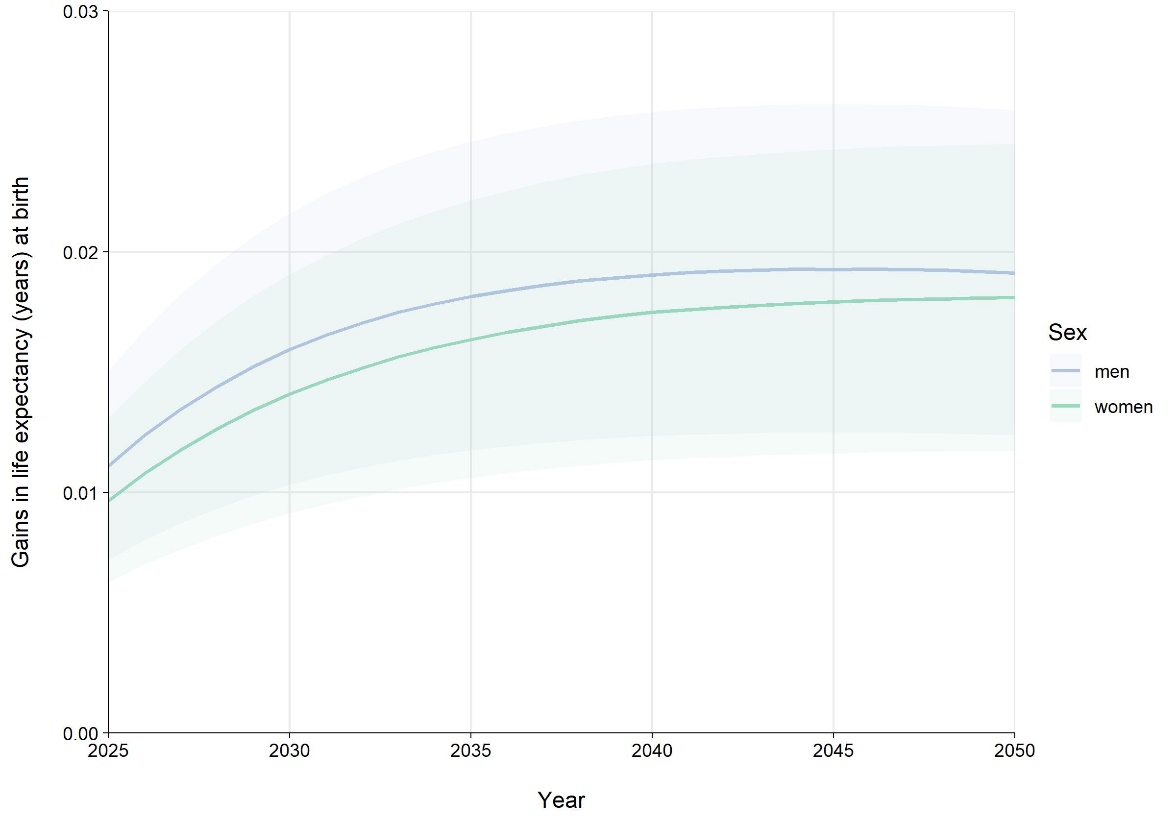


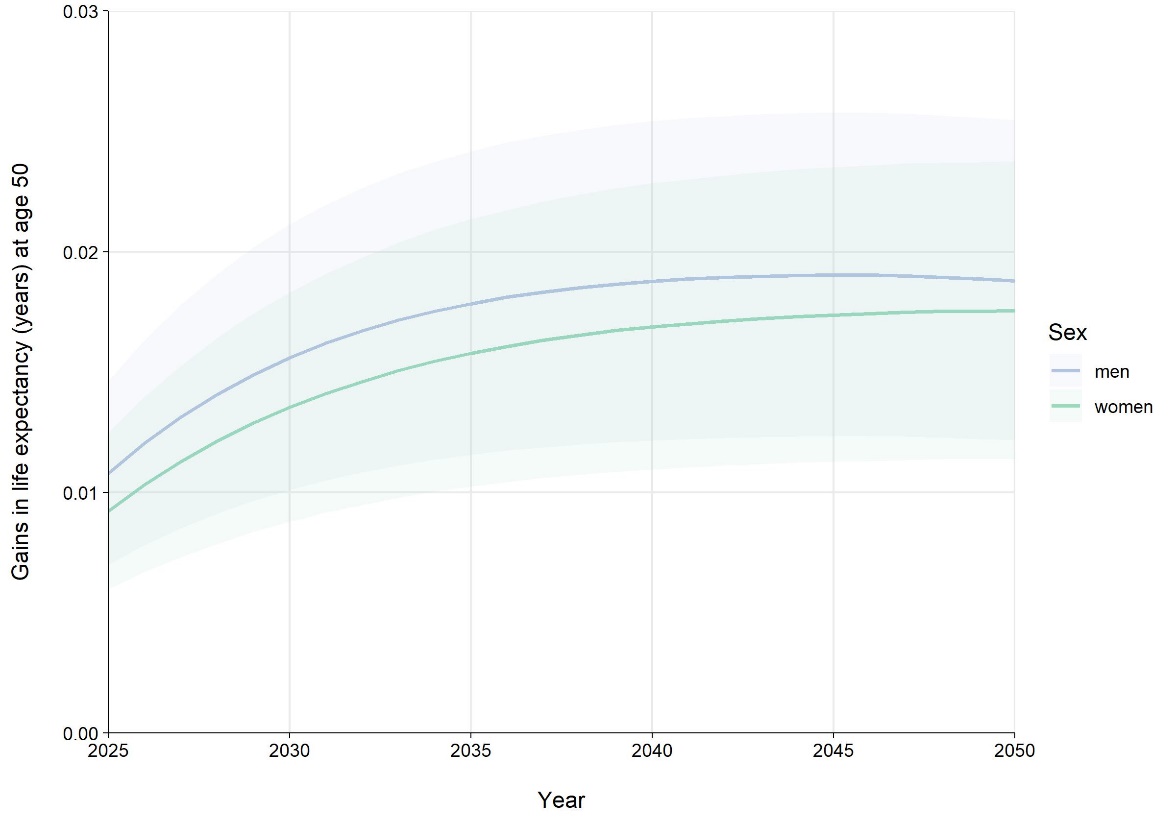


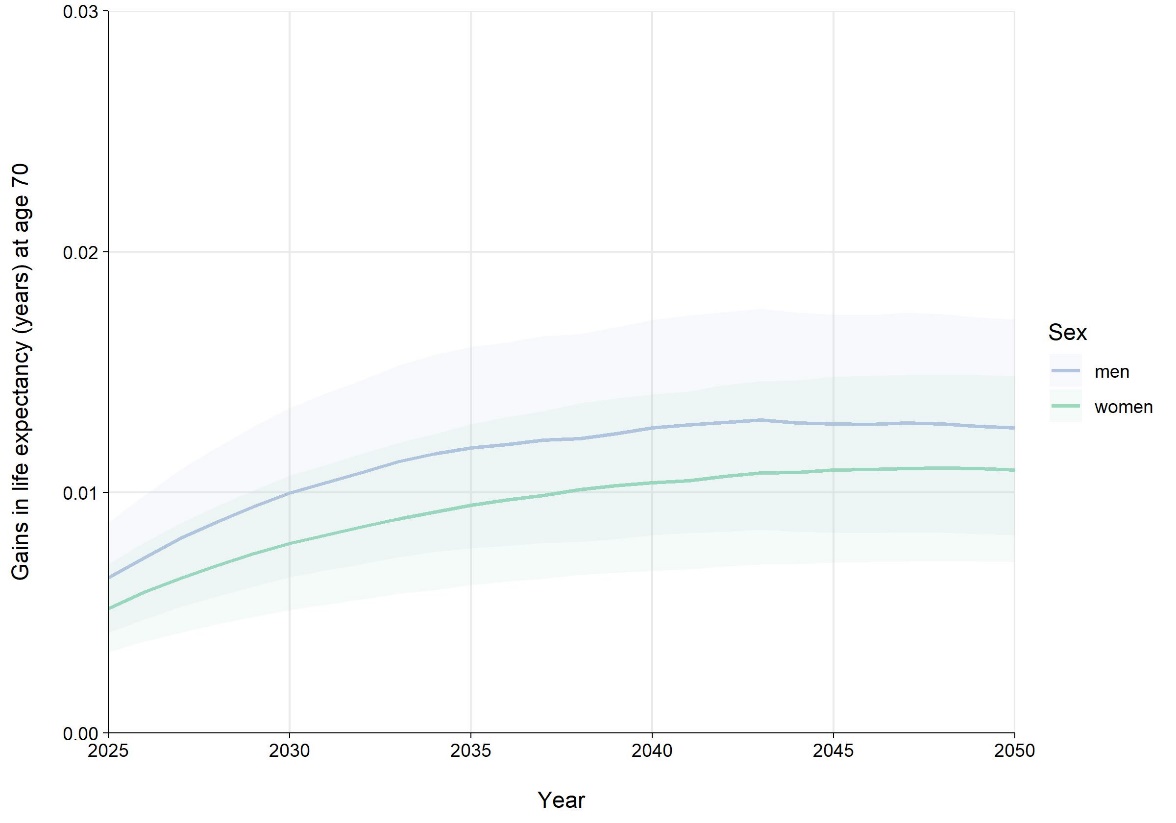


**(i2)** Dairy products, disease-free life expectancy


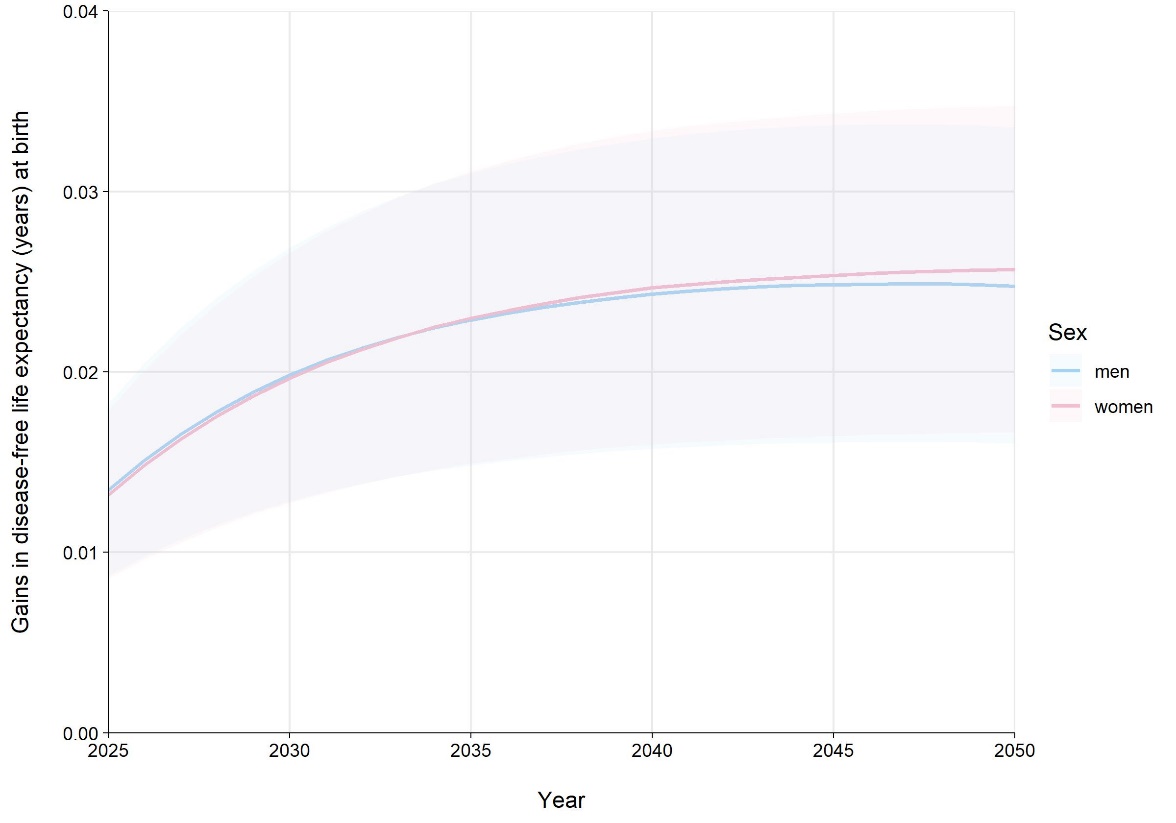


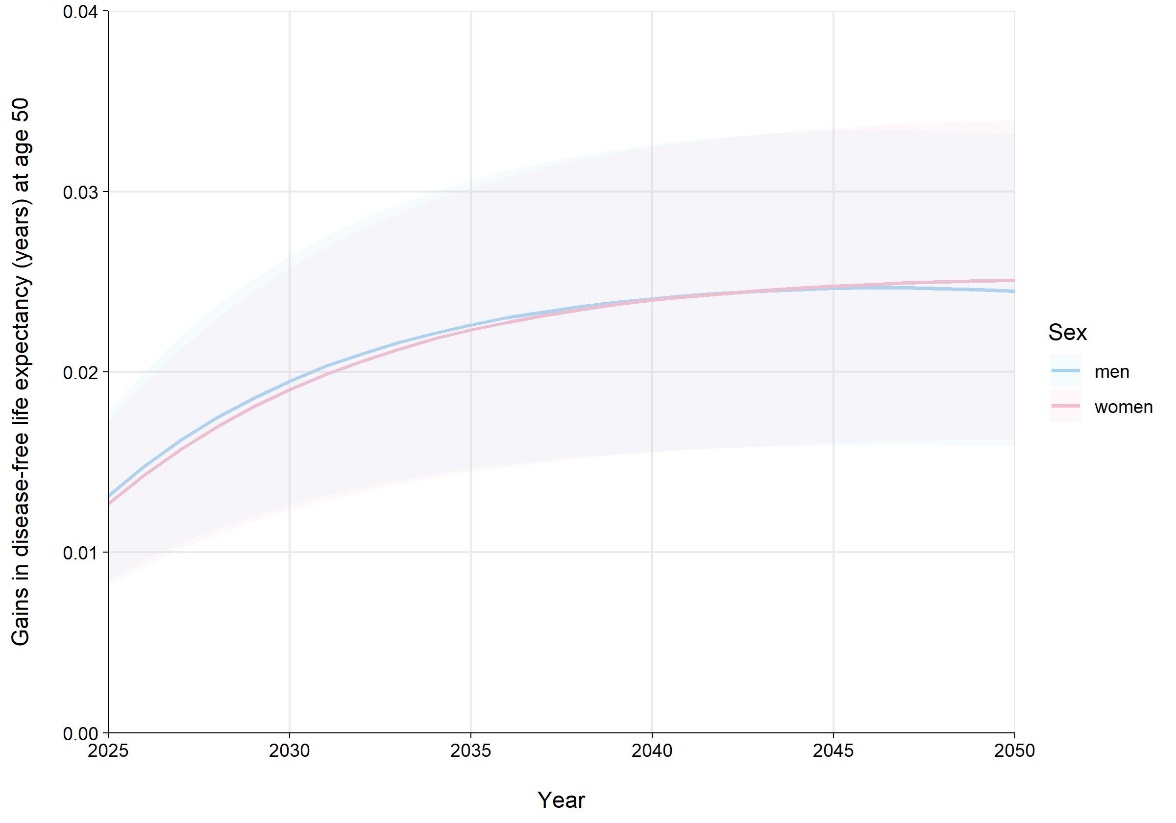


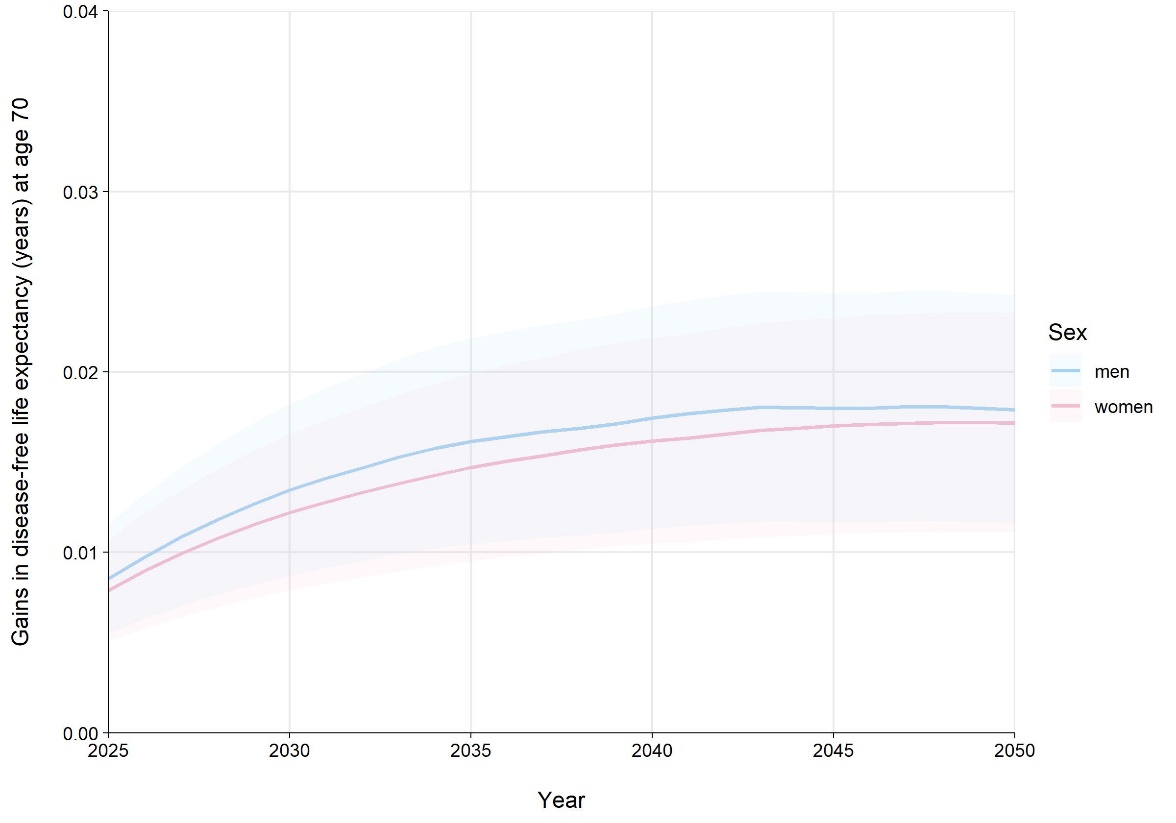


**(j1)** Tea, life expectancy


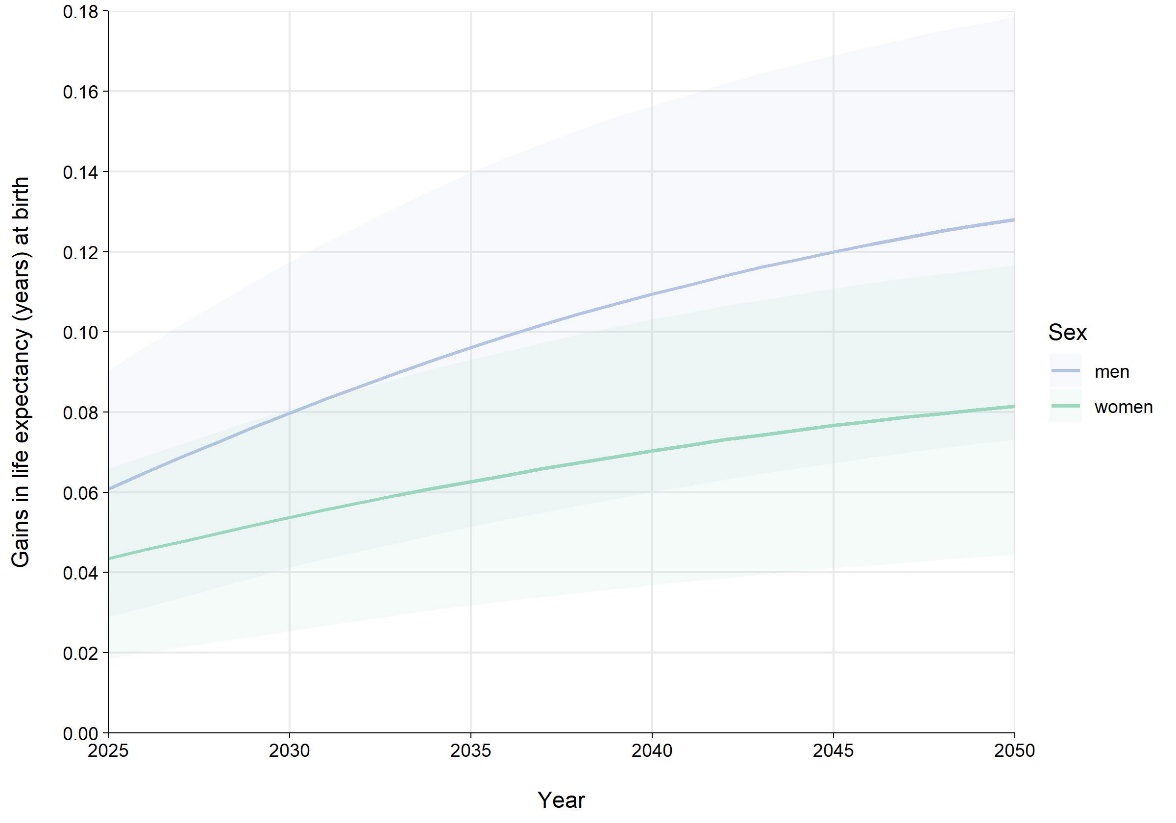


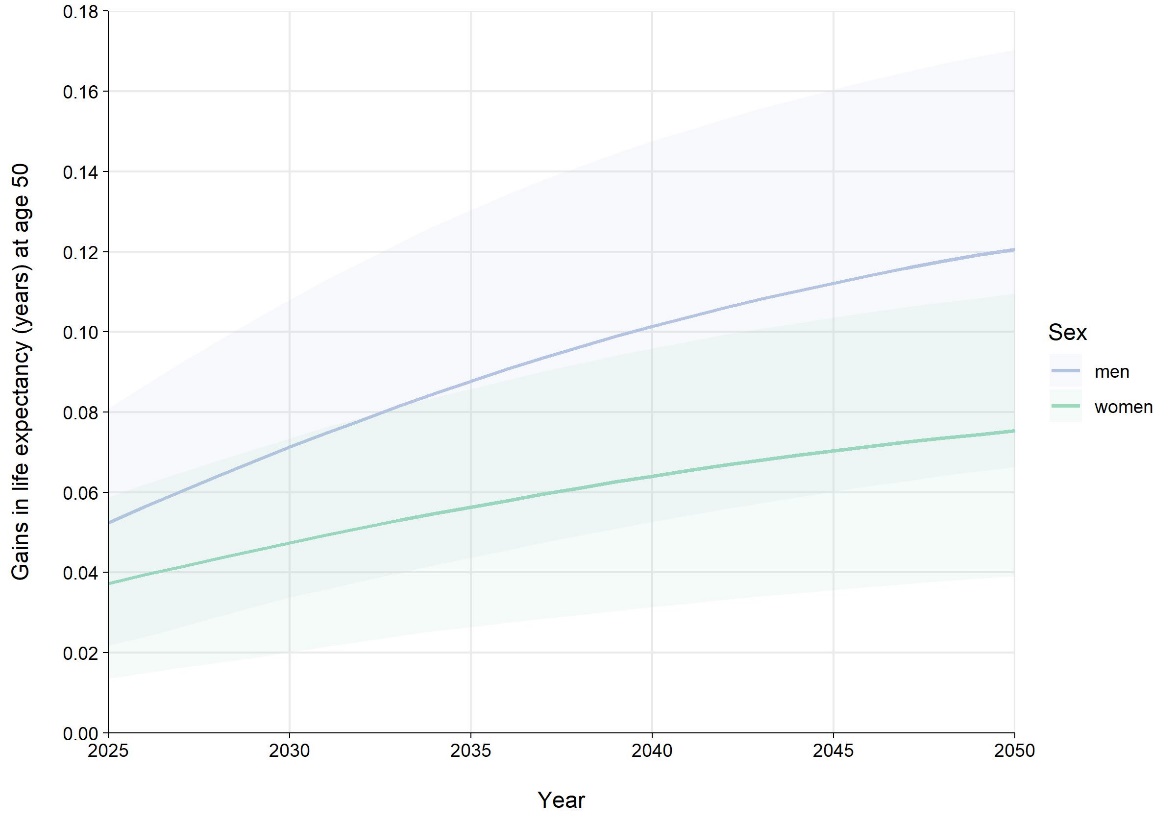


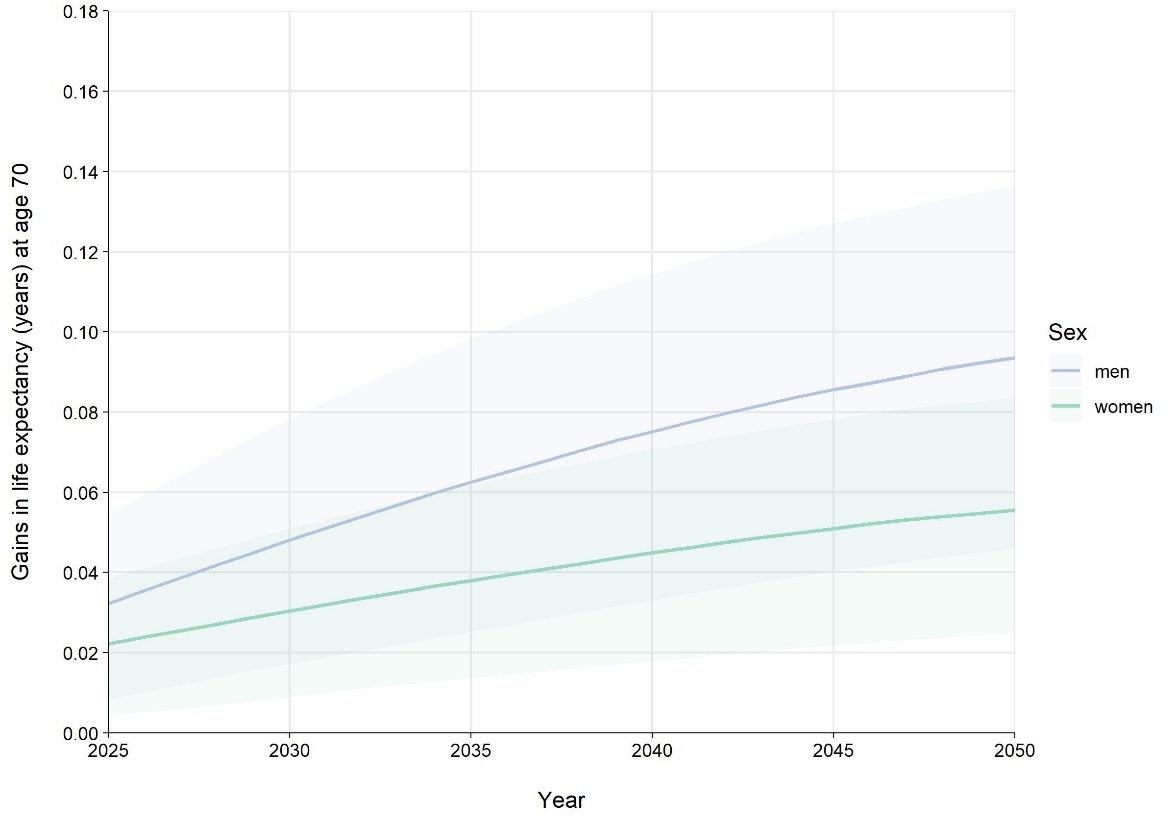


**(j2)** Tea, disease-free life expectancy


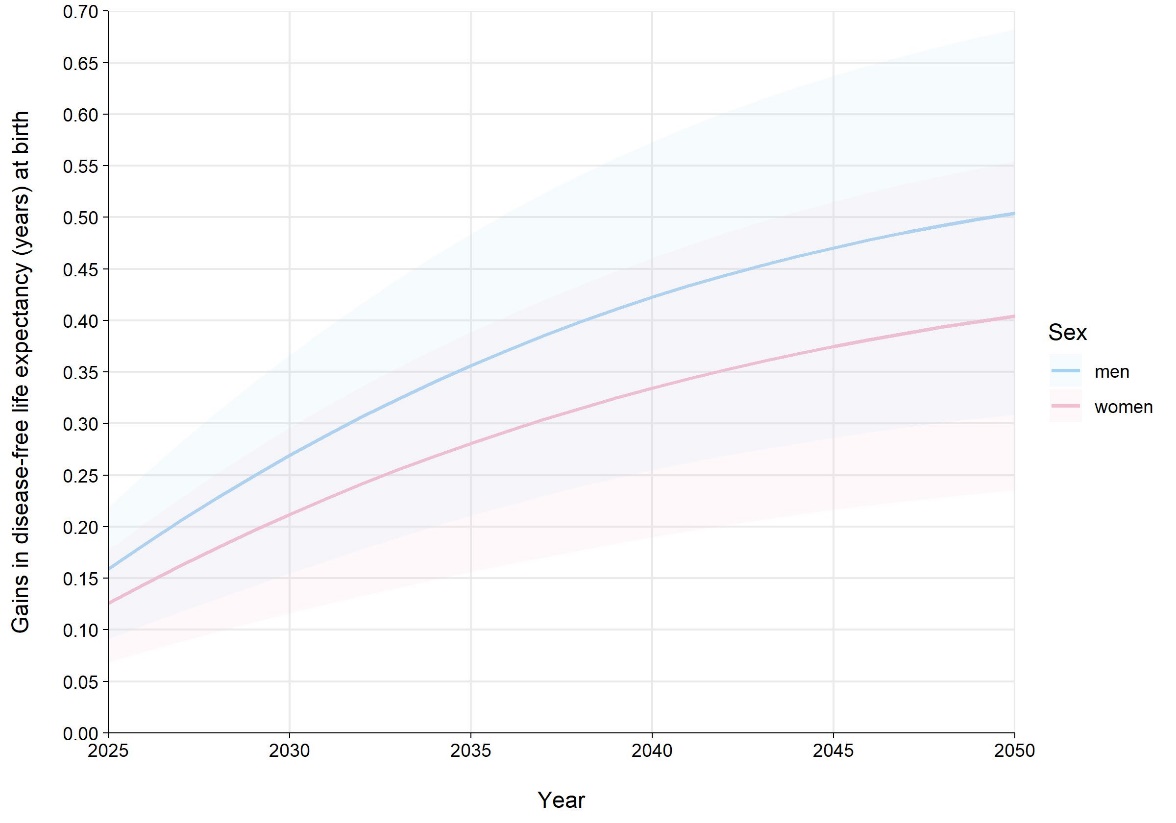


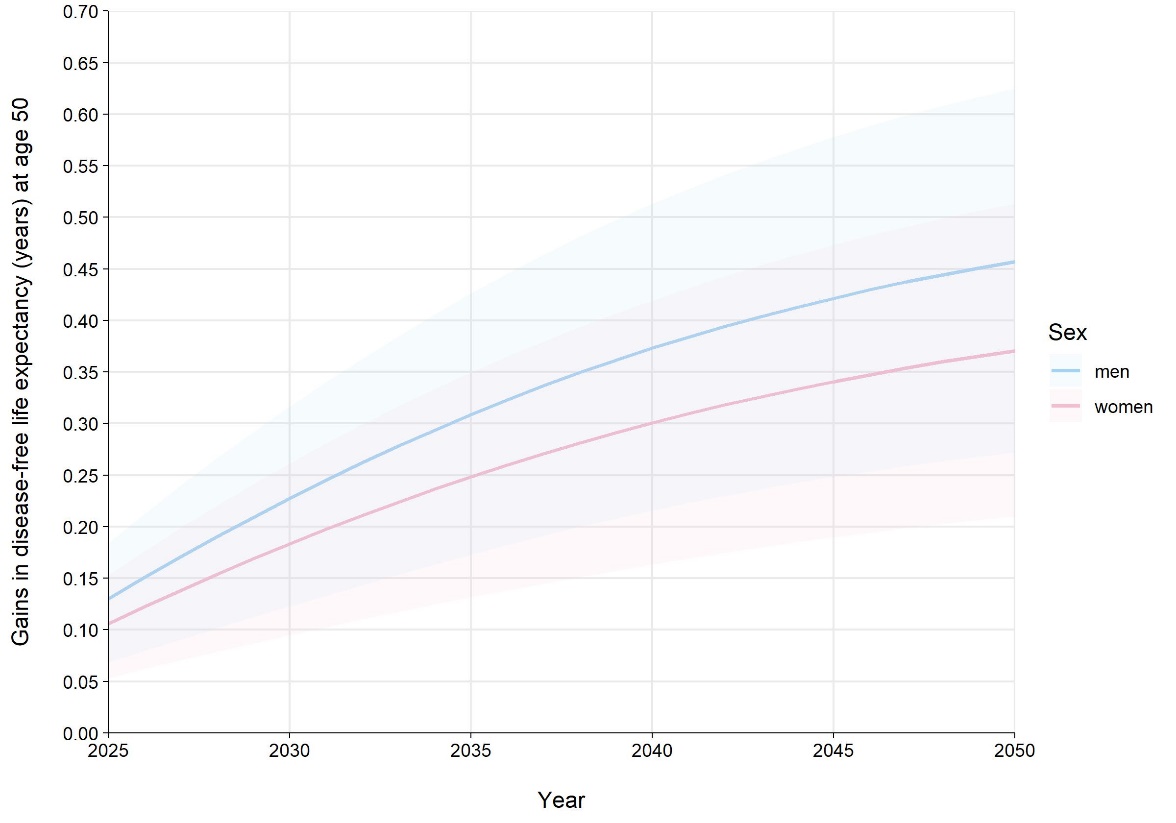


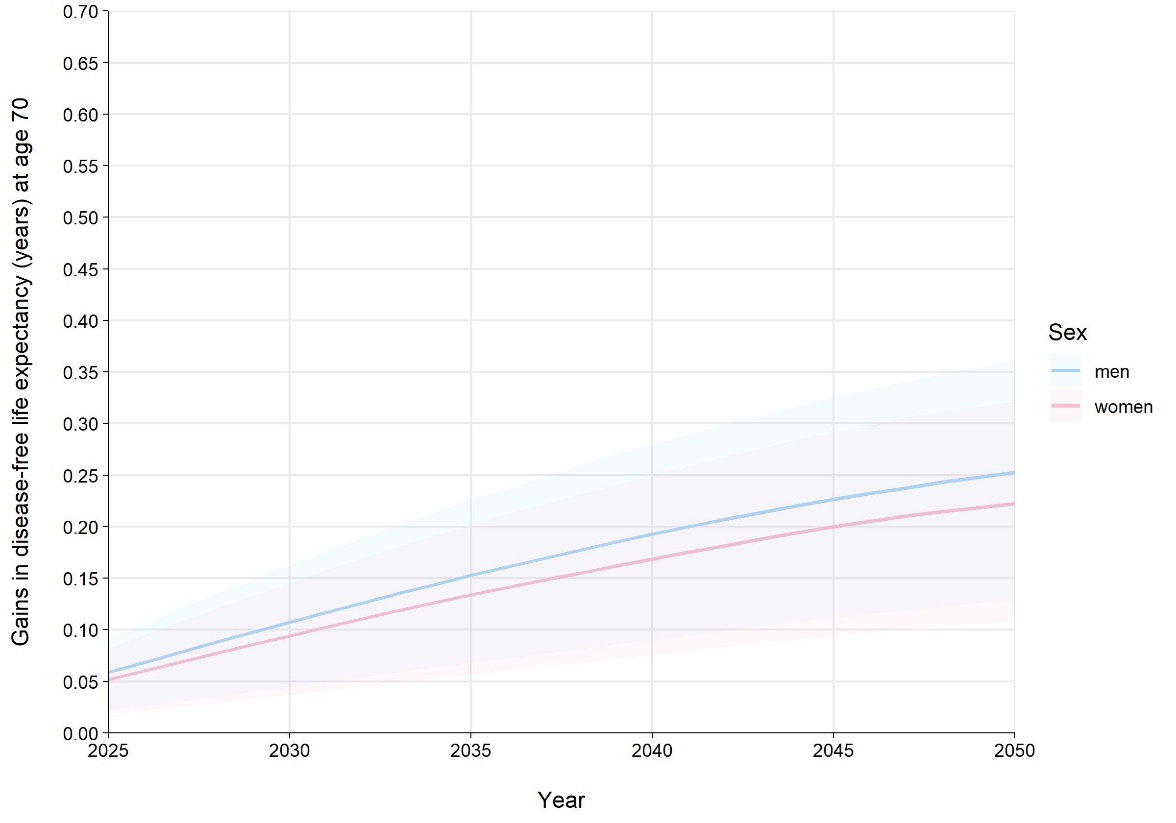


**(k1)** Sugary beverages, life expectancy


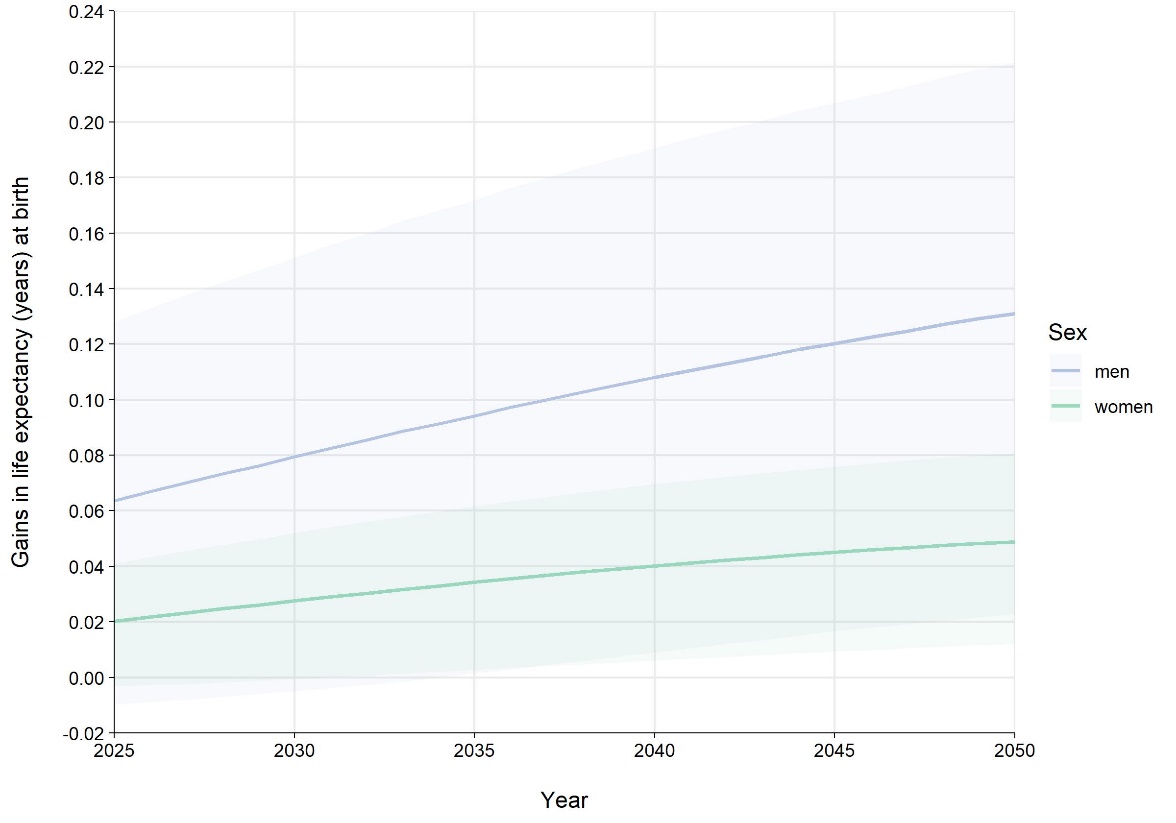


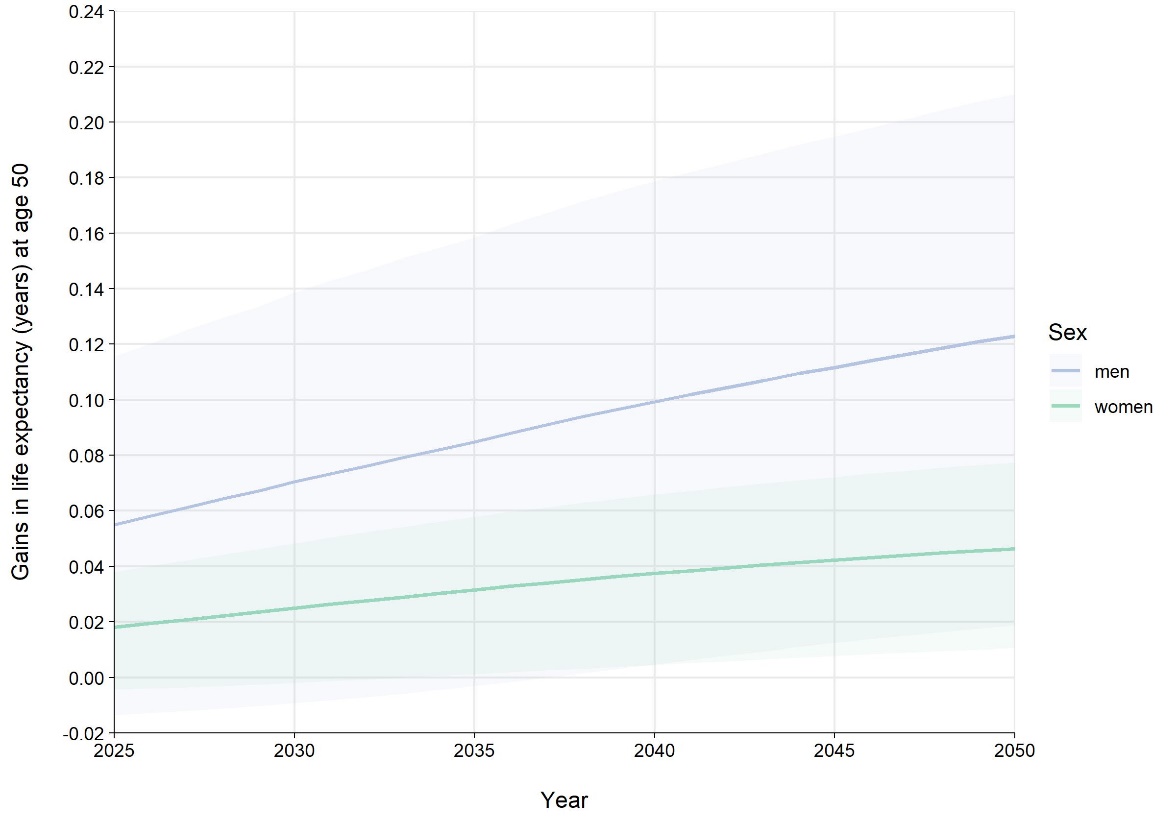


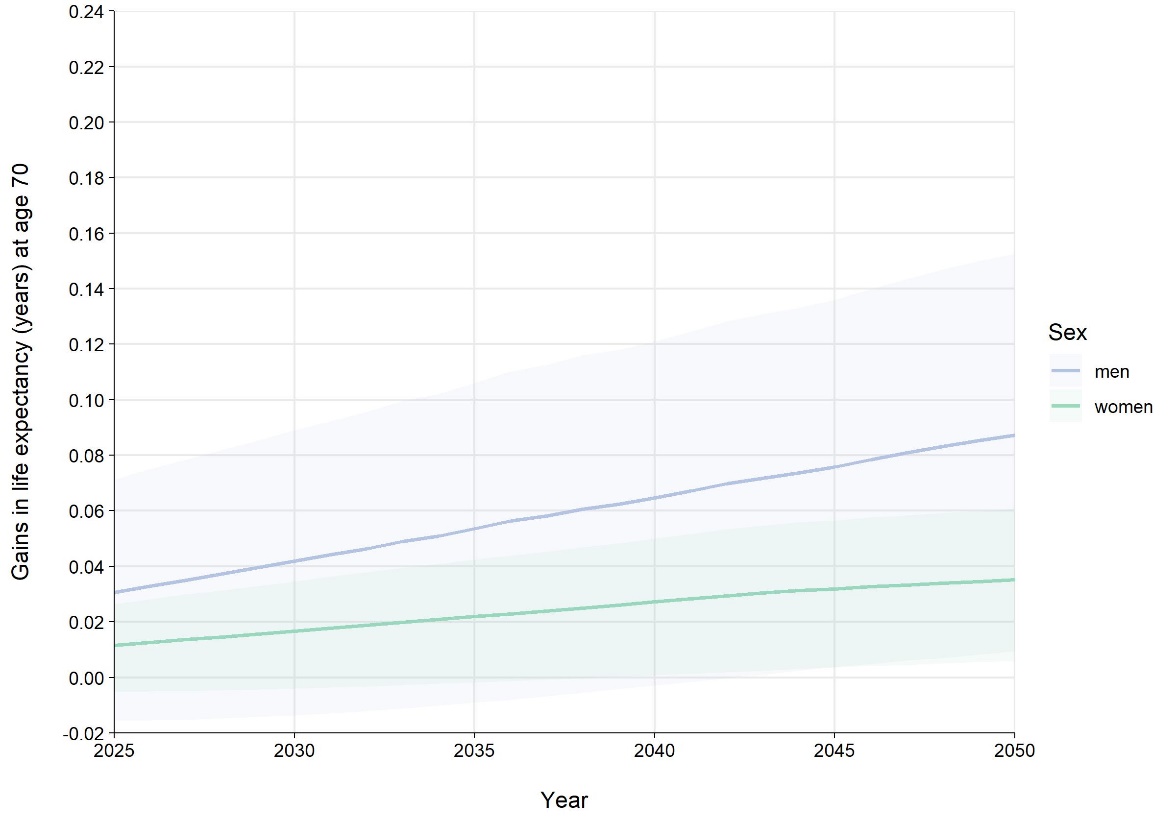


**(k2)** Sugary beverages, life expectancy


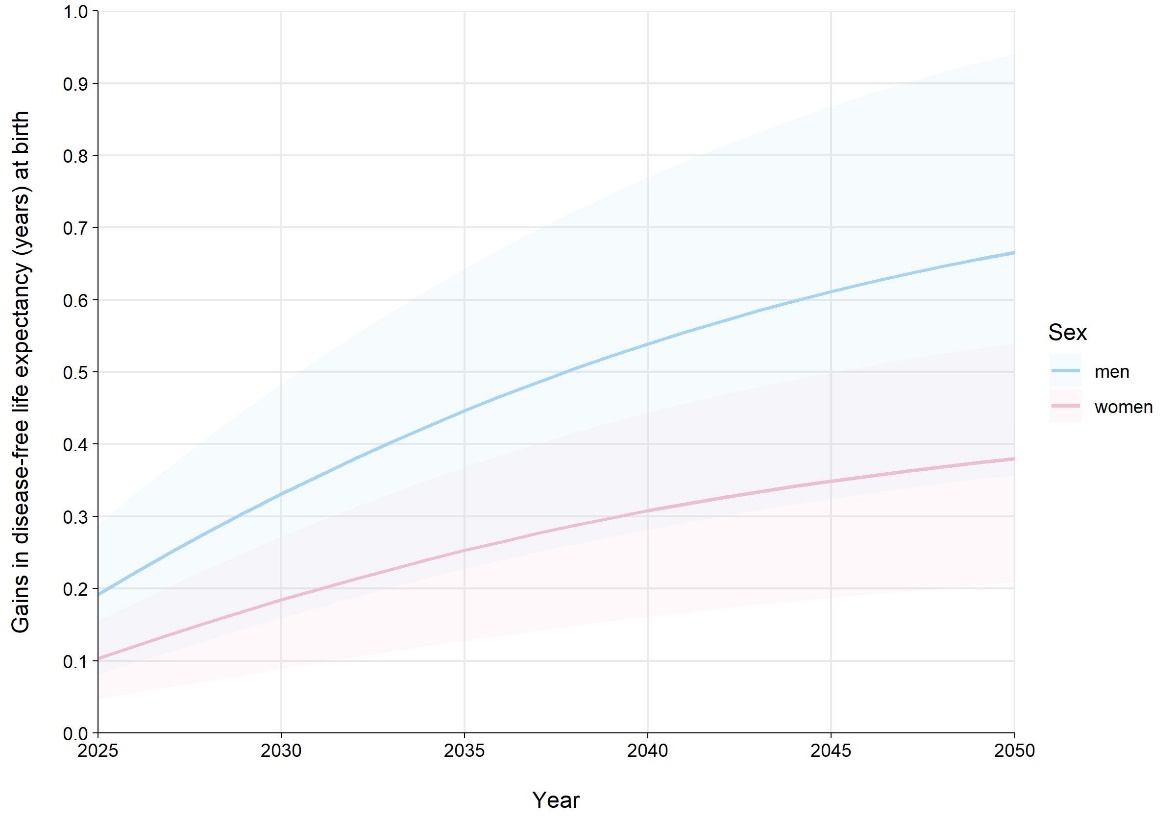


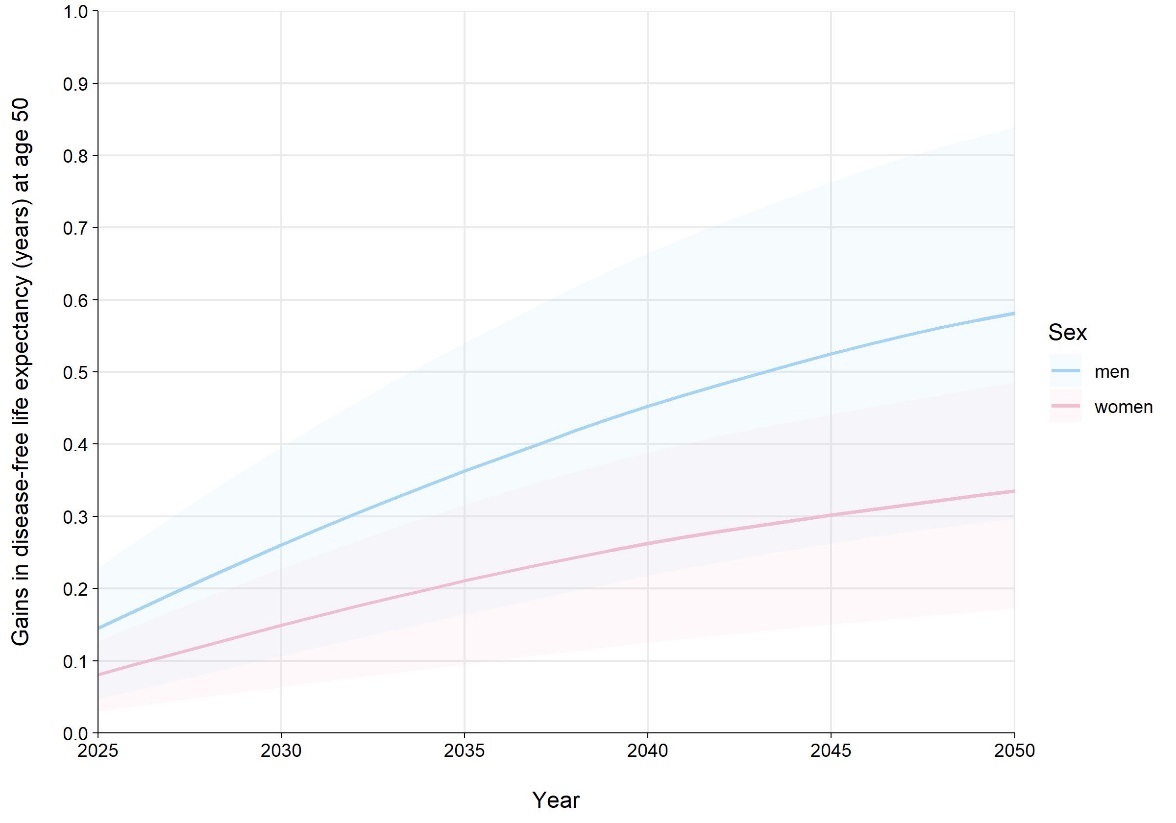


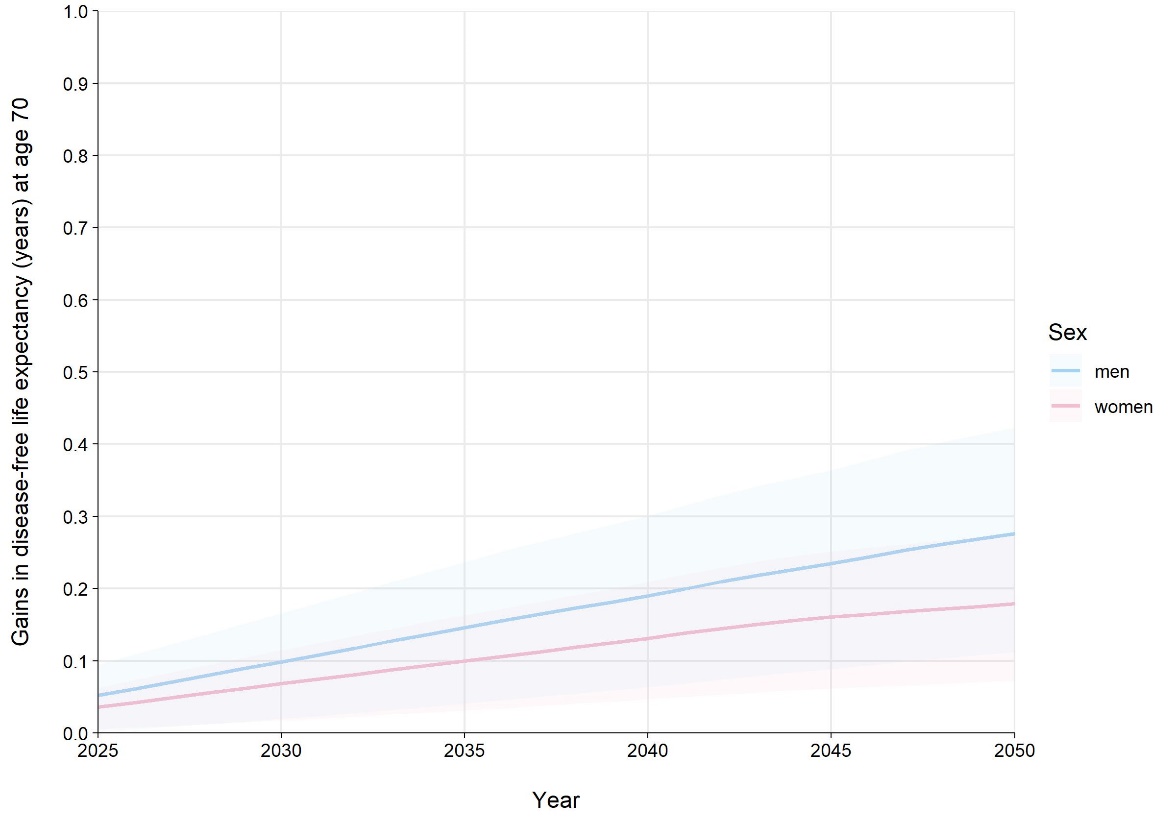

Supplement: Supplementary File [file mmc1.docx]
